# Population-Based *in Vitro* Hazard and Concentration–Response Assessment of Chemicals: The 1000 Genomes High-Throughput Screening Study

**DOI:** 10.1289/ehp.1408775

**Published:** 2015-01-13

**Authors:** Nour Abdo, Menghang Xia, Chad C. Brown, Oksana Kosyk, Ruili Huang, Srilatha Sakamuru, Yi-Hui Zhou, John R. Jack, Paul Gallins, Kai Xia, Yun Li, Weihsueh A. Chiu, Alison A. Motsinger-Reif, Christopher P. Austin, Raymond R. Tice, Ivan Rusyn, Fred A. Wright

**Affiliations:** 1 Department of Environmental Sciences and Engineering, University of North Carolina at Chapel Hill, Chapel Hill, North Carolina, USA; 2 National Center for Advancing Translational Sciences, National Institutes of Health (NIH), Department of Health and Human Services (DHHS), Bethesda, Maryland, USA; 3 Bioinformatics Research Center, and; 4 Department of Statistics, North Carolina State University, Raleigh, North Carolina, USA; 5 Department of Genetics,; 6 Department of Psychiatry, and; 7 Department of Biostatistics, University of North Carolina at Chapel Hill, Chapel Hill, North Carolina, USA; 8 National Center for Environmental Assessment, U.S. Environmental Protection Agency, Washington, DC, USA; 9 National Institute of Environmental Health Sciences, NIH, DHHS, Research Triangle Park, North Carolina, USA

## Abstract

Background: Understanding of human variation in toxicity to environmental chemicals remains limited, so human health risk assessments still largely rely on a generic 10-fold factor (10^½^ each for toxicokinetics and toxicodynamics) to account for sensitive individuals or subpopulations.

Objectives: We tested a hypothesis that population-wide *in vitro* cytotoxicity screening can rapidly inform both the magnitude of and molecular causes for interindividual toxicodynamic variability.

Methods: We used 1,086 lymphoblastoid cell lines from the 1000 Genomes Project, representing nine populations from five continents, to assess variation in cytotoxic response to 179 chemicals. Analysis included assessments of population variation and heritability, and genome-wide association mapping, with attention to phenotypic relevance to human exposures.

Results: For about half the tested compounds, cytotoxic response in the 1% most “sensitive” individual occurred at concentrations within a factor of 10^½^ (i.e., approximately 3) of that in the median individual; however, for some compounds, this factor was > 10. Genetic mapping suggested important roles for variation in membrane and transmembrane genes, with a number of chemicals showing association with SNP rs13120371 in the solute carrier *SLC7A11*, previously implicated in chemoresistance.

Conclusions: This experimental approach fills critical gaps unaddressed by recent large-scale toxicity testing programs, providing quantitative, experimentally based estimates of human toxicodynamic variability, and also testable hypotheses about mechanisms contributing to interindividual variation.

Citation: Abdo N, Xia M, Brown CC, Kosyk O, Huang R, Sakamuru S, Zhou YH, Jack JR, Gallins P, Xia K, Li Y, Chiu WA, Motsinger-Reif AA, Austin CP, Tice RR, Rusyn I, Wright FA. 2015. Population-based *in vitro* hazard and concentration–response assessment of chemicals: the 1000 Genomes high-throughput screening study. Environ Health Perspect 123:458–466; http://dx.doi.org/10.1289/ehp.1408775

## Introduction

During the past decade, considerable progress has been made in high-throughput approaches for toxicity testing to address challenges posed by *a*) expense and ethical constraints in animal testing, *b*) uncertainties in applicability of animal models to human susceptibility, and *c*) a large and increasing number of chemicals, many of which have never been subjected to adequate toxicity testing. A vision for screening by high-throughput biochemical and cell-based assays to improve understanding of toxicity response and modes of action was articulated by Collins et al. (2008). *In vitro* testing of human cell lines meets human relevance standards (Collins et al. 2008) and serves as a bridge to *in vivo* assessment. Beyond characterizing an “average” response to chemicals, next-generation toxicity testing may improve understanding of population variability, identify vulnerable subpopulations, and refine uncertainty factors used in risk assessment (Zeise et al. 2013).

The Tox21 initiative (Tice et al. 2013) is systematically screening thousands of chemicals against hundreds of molecular and cellular toxicity phenotypes. Cell-based viability assays are an established approach to prioritize chemicals or classify them into hypothesized modes of action (Huang et al. 2008). However, for environmental chemicals, the number of cell lines has typically been limited to dozens (Lock et al. 2012; O’Shea et al. 2011), sometimes representing multiple species (Xia et al. 2008). Thus, an understanding of human population variability and the role of constitutional genetic variation remains elusive. Epidemiological approaches have been limited to a few chemicals with high occupational or other exposure (Zeise et al. 2013), or have quantified polymorphic toxicokinetic variation mainly in drug-metabolizing enzymes (Ginsberg et al. 2009). Epidemiological studies provide little basis to compare chemicals, including new chemicals with little or no data, and risk assessments still typically assume that more sensitive individuals or subpopulations are adequately protected by applying an “uncertainty” factor of 10, the product of factors of 10^½^ each for toxicokinetics and toxicodynamics (Zeise et al. 2013).

Screening of lymphoblastoid cell lines (LCLs) is an established approach to identify genetic variants that influence cytotoxic response to pharmaceuticals, especially chemotherapeutic agents (Wheeler and Dolan 2012). Choy et al. (2008) challenged the value of these approaches, primarily because of the effects of growth rates and technical factors. However, enrichment of human blood expression quantitative trait loci has been established among weakly significant chemotherapeutic drug-susceptibility loci (Gamazon et al. 2010). With the advent of statistical methods that are purpose-built for cytotoxicity profiling, several robust associations have been identified (Brown et al. 2014).

For environmental chemicals, the extent of population variation in *in vitro* cytotoxicity may serve as a surrogate for cellular variation in the toxicodynamic relationship between systemically available concentrations and toxic responses (Zeise et al. 2013). Such data could inform a chemical-specific adjustment factor for human toxicodynamic variability, replacing the usual factor of 10^½^ [International Programme on Chemical Safety (IPCS) 2005]. Direct connections to human risk assessment must consider genetic variation at low concentrations relevant to human exposure. This goal may conflict somewhat with maximization of power to identify specific genotype–susceptibility associations because the effects of genetic variation may be apparent only at higher concentrations. Furthermore, for both these goals, the sample sizes in studies of environmental chemical cytotoxicity has often been inadequate to establish population variation or to assess genetic association for these complex traits with small effect.

Here, we describe profiling 1,086 LCLs for cytotoxic response to 179 chemicals, each assayed over a range of eight concentrations spanning six orders of magnitude. The compounds were primarily chemicals of environmental concern, cover a wide range of *in vivo* toxicity hazards, and were drawn from a larger set of 1,408 compounds used for high-throughput screening (Lock et al. 2012; O’Shea et al. 2011; Xia et al. 2008). We selected the LCLs from the 1000 Genomes Project (1000 Genomes Project Consortium et al. 2012), spanning a variety of ancestral populations. We assessed cytotoxic response using an EC_10_ (effective concentration, 10th percentile) and performed genome-wide association mapping using both EC_10_ and the entire eight-concentration profile as a multivariate vector.

## Materials and Methods

*Chemicals and cytotoxicity profiling*. The chemicals evaluated were a subset of the National Toxicology Program’s 1,408 chemical library as described by Xia et al. (2008). We dissolved chemicals with dimethyl sulfoxide (DMSO) into eight stock concentrations transferred into 1,536-well plate format via a pin tool station (Kalypsys Inc.). The final concentrations ranged from 0.33 nM to 92 μM. The negative control was DMSO at 0.46% vol/vol, and the positive control was tetra-octyl-ammonium bromide (46 μM). We used the CellTiter-Glo Luminescent Cell Viability (Promega) assay to assess intracellular ATP concentration, a marker for viability/cytotoxicity, 40 hr after treatment. We used a ViewLux plate reader (PerkinElmer) to detect luminescent intensity.

*Cell lines*. We acquired 1,104 immortalized lymphoblastoid cell lines from the Coriell Institute. We randomly divided cell lines into screening batches, equally distributed by population and sex in each batch without regard to family structure. We cultured cells at 37°C with 5% CO_2_ in RPMI 1640 media (Invitrogen) supplemented with 15% fetal bovine serum (HyClone) and 100 U/mL penicillin/100 mg/mL streptomycin (Invitrogen), replacing media every 3 days. We plated cells with viability of > 85% into tissue culture–treated 1,536-well white/solid bottom plates (Greiner Bio-One) at 2,000 cells/5 μL/well using a flying reagent dispenser (BioRAPTR, Beckman Coulter). We seeded each cell line on multiple plates (1–2 plates within or between batches). We fit all chemicals to a single plate.

*Genotypes*. The primary genotypes were the Illumina HumanOmni2.5 platform (ftp://ftp.1000genomes.ebi.ac.uk/vol1/ftp/technical/working/20120131_omni_genotypes_and_intensities) and available for 1,086 lines, excluding SNPs with call rate < 95%, minor allele frequency (MAF) < 0.01, or HWE *p*-value < 1 × 10^–6^. We chose a maximal subset of 884 samples to remove first-degree relatives (“unrelated” set) using genotypes and sample annotation. Of the 884 samples, genotyped SNPs from the platform were available for 761. The remaining 123 samples were genotyped by HapMap (http://hapmap.ncbi.nlm.nih.gov/downloads/genotypes/hapmap3_r3/plink_format), and we imputed for the filtered Illumina SNPs using MaCH (Li et al. 2010). We used a set of 875 samples from the 1000 Genomes set (not restricted to these cell lines) as an imputation reference, producing 1.3 million SNPs for primary analysis. A further subset of 690 unrelated individuals from 1000 Genomes Phase I had more complete sequencing data, with a total of 12 million filtered SNPs.

*Cytotoxicity EC_10_ estimation, outlier detection, and variability characterization*. We normalized cytotoxicity data (see Supplemental Material, Figure S1) relative to positive/negative controls. Although the primary association mapping method was a multivariate treatment of cytotoxocity response across all concentrations for each chemical, we also used a single cytotoxicity dose summary per chemical and cell line. We devised an EC_10_ using the logistic model

log[(η–θ_min_)/(θ_max_ *–* θ_min_)] = β_0_ + β_1_*d*, *y* = η + ε, [1]

where ε ~ *N*(0, σ^2^), *y* is the observed normalized signal representing the proportion of surviving cells (the “cytotoxicity value”), *d* is log(concentration), and θ_max_ is the response value at zero concentration. We set θ_min_ = 0 to avoid estimation difficulties for chemicals with low cytotoxicity. We made an exception for a very few chemicals for which the cytotoxicity value at the highest concentration was > 0.4, fixing θ_max_ using the observed cytotoxicity at the maximum concentration, and inspection revealed good fits in such instances. Although, in principle, θ_max_ should have been 1.0, several plates exhibited a drift from this value, and the parameter was estimated from the data.

We used maximum likelihood by numerical optimization in *R* v2.15 (R Core Team 2012) to fit [β_0_, β_1_, σ^2^, θ_max_]. We devised automatic outlier detection, dropping each concentration value in succession and flagging values for which the maximum likelihood improved by a factor of ≥ 10 (see Supplemental Material, Figure S2), refitting the model using non-outlying observations.

We characterized interindividual variability using the distribution of estimated EC_10_ across cell lines. Summary statistics, including the mean, SD, and selected quantiles (q_01,_ q_05,_ q_95,_ and q_99_), were calculated for log(EC_10_) (see Supplemental Material, Table S1). For risk assessment, the relevant variability measure is the ratio of EC_10_ for the median compared with a “sensitive” individual, because the uncertainty factor is intended to cover the more sensitive population “tail” (i.e., those for whom a lower concentration elicits effect). There is no standard definition for a sensitive population threshold, so we selected 1% as a nominal value that could be estimated reliably from a sample size of 1,000 individuals, and we defined a toxicodynamic variability factor as 10^q50–q01^ analogous to a chemical-specific adjustment factor for human toxicodynamic variability.

*Attenuated variability estimates to account for sampling variation*. To account for the inflationary effect of sampling variance, we considered the model logEC_10_ = μ + ε, where μ is the underlying true (unknown) logEC_10_ and ε represents sampling variation. We assumed each chemical has an underlying true sampling variability of σ*_s_*^2^ per observation; observed log EC_10_ values were, in many instances, averaged across multiple observations. For an individual measured *n*´ times, var(ε) = σ*_s_*^2^/*n*´. We conservatively estimated σ*_s_*^2^ by computing the sample variance for paired replicate instances for the chemical across different batches and averaging across pairs. Then we computed a variance inflation factor (VIF):

*VIF* = var(logEC_10_) ÷ [var(logEC_10_) – ^^^σ*_s_*^2^/mean(*n*´)], [2]

where mean(*n*´) is the average number of replicates per individual. Finally, we considered individual measurements to have been inflated by *VIF*^1/2^ so that, for example, the “shrunken” toxicodynamic variability factor is 10^(q50–q01)/^*^VIF^*^^1/2^^.

*Comparison with estimated* in vivo *toxicodynamic variability*. The World Health Organization recently reviewed available data on human *in vivo* toxicodynamic variability as part of a new harmonized framework (IPCS 2014). For each of the available data sets, variation in systemic concentration eliciting a toxic response was represented by a geometric SD (GSD) for population toxicodynamic variability based on fitting to a log-normal distribution [Tables A4.5 and A4.6 in IPCS (2014)]. We calculated an analogous toxicodynamic variability factor using our *in vitro* data as the ratio of the median to the 1% quantile, equal to GSD^2.326^, where the exponent is the 99% standard normal quantile, forming a basis for comparison with *in vivo* summaries.

*Multivariate association analysis*. We used MAGWAS multivariate analysis of covariance model (Brown et al. 2012b) for primary association mapping. The approach uses the full concentration–response profile instead of a univariate summary (such as EC_10_), with advantages in robustness and power under a variety of association patterns. The model for the *j*th individual and genotype *i* for the chemical/SNP is

*Y_ij_* = *X_ij_*β + μ*_i_ + e_ij_ ~ N*(0, Σ), [3]

where *Y_ij_* is the response vector (across eight concentrations) for the *j*th individual having genotype *i*; *X_ij_* is the design matrix of covariates, including sex, indicator variables for laboratory batch, and the first 10 genotype principal components, and μ*_i_* is the eight-vector of parameters modeling the effects of genotype *i*. The multivariate normal error model allows dependencies in the variance–covariance matrix Σ. We obtained *p*-values using Pillai’s trace (Pillai 1955). Because this method makes use of asymptotic theory, we removed markers with < 20 individuals representing any genotype, leaving 692,013 SNPs for analysis.

*Heritability*. We calculated the proportion of chemical response variation due to genetic variation (heritability) for each compound using the mean batch-adjusted EC_10_ value across the 401 related individuals belonging to nuclear family trios. We used the Multipoint Engine for Rapid Likelihood Inference (MERLIN) (Abecasis et al. 2002) package to estimate heritability. Consideration of covariates, including subpopulation by ethnicity [Utah residents with European ancestry (CEU), Mexican ancestry in Los Angeles (MXL), and Nigeria (YRI)] and population stratification (first three principal components) did not have a substantial effect (not shown). In addition, we performed variance component analysis and hypothesis testing with Sequential Oligogenic Linkage Analysis Routines (SOLAR) (Almasy and Blangero 1998) to evaluate the significance and standard error for each heritability.

Using the 884 unrelated individuals, we also ran genome-wide complex trait analysis (GCTA) (Yang et al. 2011) to estimate heritability, using default settings and the 1.3 million SNPs. To assess whether the concordance between MERLIN and GCTA was as expected, we used the 179-vector of MERLIN heritability estimates as a hypothetical true set of heritabilities. We used these “true” values and associated standard errors from both MERLIN and GCTA to simulate independent normal errors to create 10,000 paired vectors of MERLIN and GCTA estimates, which we then compared.

## Results

*Cell lines and genotypes*. An initial set of 1,104 LCLs was representative of nine geographically and ancestry-diverse populations: Utah residents with European ancestry (CEU); Han Chinese in Beijing, China (CHB); Japanese in Tokyo, Japan (JPT); Luhya in Webuye, Kenya (LWK); Mexican ancestry in Los Angeles, California (MXL); Tuscans in Italy (TSI); Yoruban in Ibadan, Nigeria (YRI); British from England and Scotland (GBR); and Colombian in Medellin, Colombia (CLM). A few cell lines (18; 1.6%) were not viable or grew very slowly, or they had insufficiently available genotypes; therefore, the final set consisted of 1,086 cell lines.

To reduce multiple comparisons, we initially focused on approximately 1.3 million markers typed on the Omni 2.5 platform and further filtered by MAF. Because 172 individuals had not been genotyped on the platform, dosage imputation was performed using the appropriate 1000 Genomes reference population. We performed separate analyses on 400 individuals belonging to parent–child trios (not all complete) in the CEU (164), MXL (83), and YRI (153) populations, and on a maximal set of 884 individuals in the remaining populations with no first-degree relationships (unrelateds). We also performed association analyses using a larger set (~ 12 million) of typed SNPs available from the sequencing data.

**Figure 1:**
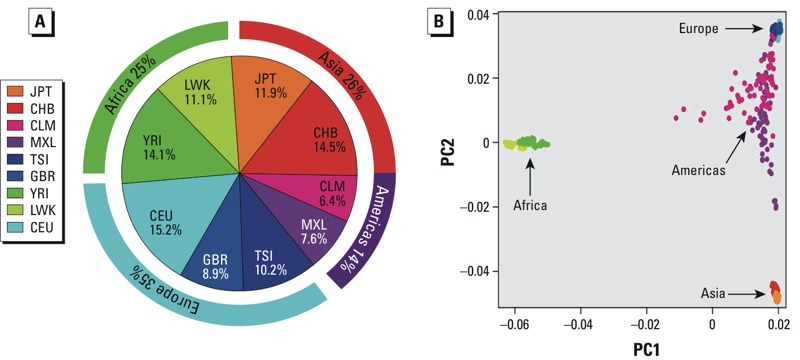
(*A*) Distribution of the lymphoblastoid cell lines (LCLs) used in this study among the nine populations. Outer boundaries show continental/ancestral origin. (*B*) Scatter plot for the 1st and 2nd principal components for genotypes across all cell lines; colors represent populations shown in (*A*).

Figure 1A shows the distribution of populations and continental ancestry. We randomly divided LCLs into screening batches with equal distribution of populations and sex in each batch, without regard to family structure. The major HapMap/1000 Genomes continental ancestry populations were represented, as well as admixed populations from the Americas (Figure 1B).

*Cytotoxicity profiling*. Supplemental Material, Figure S1, shows a flow chart of the data analysis from cytotoxicity profiling across eight concentrations ranging from 0.33 nM to 92 μM. We used logistic curve fitting with outlier detection (see Supplemental Material, Figure S2) to obtain EC_10_ values, which were batch-corrected and averaged across replicates for each cell line.

**Figure 2:**
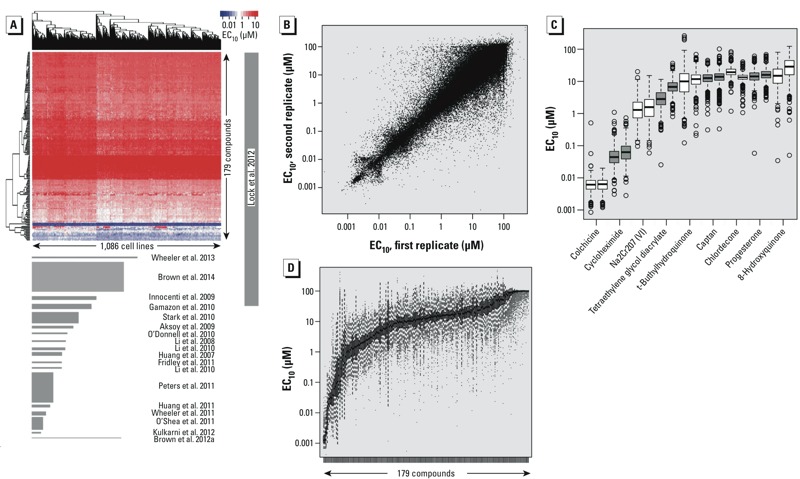
(*A*) Comparison of the present study with other comparable lymphoblastoid cell line (LCL) cell line/screening studies, in terms of the number of cell lines and chemicals screened. EC_10_ values are shown in the heat map (top), and the area of each report is shown in proportion to the present study (bottom); the numbers of cell lines and compounds used in the published studies are listed in Supplemental Material, Table S2. (*B*) Intraexperimental reproducibility of EC_10_ values for randomly selected pairs of within-batch replicate plates for all chemicals and cell lines. (*C*) EC_10_ values for nine compounds assayed in two independent sets of wells on each plate, shown as side-by-side box plots. Boxes represent interquartile range, lines within boxes are medians, whiskers represent values 1.5*(interquartile range) from the first and third quartiles, and circles indicate outliers. (*D*) Box plot showing variation of cytotoxicity EC_10_ values for the 179 chemicals (arranged by mean activity) across the 1,086 cell lines.

To place our study in context, we reviewed comparable studies, identifying 19 reports (see Supplemental Material, Table S2). These studies had more than one chemical, except for Brown et al. (2012a), and at least 50 cell lines. Figure 2A depicts a heat map of the cytotoxicity measurements across cell lines and chemicals, and shows, to scale, the size of the other studies in terms of cell lines and number of chemicals/drugs. In these terms, our study is an order of magnitude greater than any single previous study, and several times larger than the other reports combined.

For approximately 700 cell lines for which there was at least one replicate plate, Figure 2B depicts the EC_10_ values for replicates (*r* = 0.90). We assayed 9 of the chemicals in duplicate on each plate, and duplicate chemicals showed similar median EC_10_ values and ranges of variability (Figure 2C). The entire range of EC_10_ values across all chemicals exhibited remarkable cytotoxicity variation (Figure 2D). Only one other report has been of similar scale in number of chemicals [240 chemicals investigated by Lock et al. (2012)]. However, our comparisons are much more definitive in the ability to rank and prioritize compounds by cytotoxic activity because of the large number of cell lines [*n* = 1,086 used here vs. *n* = 81 described by Lock et al. (2012)].

**Figure 3:**
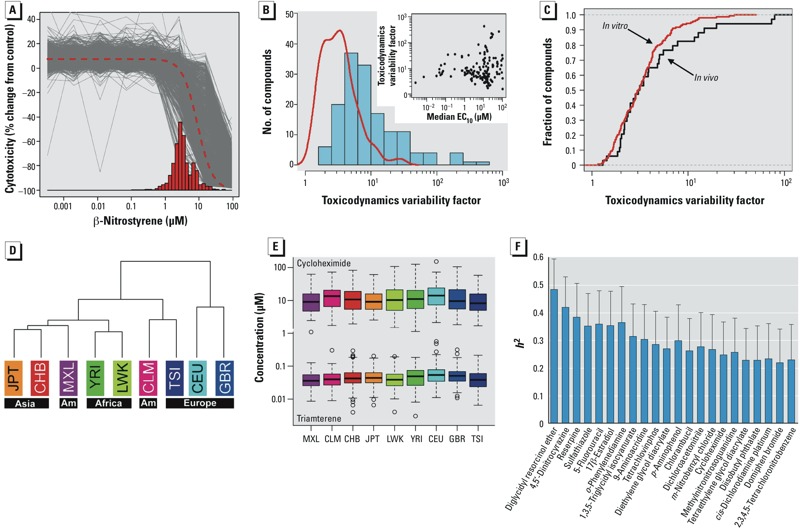
(*A*) Modeling *in vitro* quantitative high-throughput screening data, using β-nitrostyrene as an example chemical. Logistic dose–response modeling was performed for each individual (plate), as shown by thin gray lines. Bars represent individual EC_10_ values, and the dashed curve represents the fit of the logistic model to the pooled data. EC_10_ estimation based on this curve is similar to the average of the individual EC_10_ values. (*B*) Histogram (bars) of the toxicodynamic variability factor 10^(q50–q01)^ for 149 compounds across 1,086 cell lines. The curve shows the same distribution when values are shrunken to account for technical variability; for the 30 compounds not shown, estimated technical variability was too large to calculate a shrunken factor. The inset shows the relationship between range and median estimated EC_10_ for each chemical. (*C*) Cumulative distribution functions for the *in vitro* toxicodynamic variability factor shrunken to account for technical variability 10^(q50–q01)/VIF^^1/2^, across 149 compounds (present study) and the human *in vivo* toxicodynamic variability factors across 34 compounds (IPCS 2014). (*D*) Hierarchical clustering for the 179-length profiles of mean EC_10_, computed within each population, and shown by continental ancestral origin of the population. AM, Americas. (*E*) Box plot of EC_10_ values by population for two example chemicals with different potency levels, showing significant population differences by analysis of variance (top; cycloheximide, *p* = 6.0 × 10^–6^; bottom; triamterene, *p* = 3.6 × 10^–4^). Boxes represent interquartile range, lines within boxes are medians, whiskers represent values 1.5*(interquartile range) from the first and third quartiles, and circles indicate outliers. (*F*) Trio-based heritability estimates (*h^2^*) for compounds with evidence of additive heritability (22 chemicals with *p* < 0.05 are shown, the top 17 having *q* < 0.2).

Figure 3A shows EC_10_ estimation for all cell lines for an illustrative chemical β-nitrostyrene, as well as results from the logistic fit applied to the pooled data. The histogram depicts individual EC_10_ estimates, showing overall variation of more than an order of magnitude. To quantify sensitivity variation, we recorded the 1st and 50th percentiles of log EC_10_ values for each chemical, and refer to the natural-scale quantile difference 10^(q50–q01)^ as a “toxicodynamic variability factor.” Figure 3B shows the range in these factors across chemicals and as a function of median EC_10_ values. The figure also shows a shrunken estimate of the true underlying distribution after removing inflation due to pure sampling variation. For 30 chemicals a shrunken estimate could not be determined, so only 149 chemicals are shown. About half of these chemicals show a shrunken range < 10^½^; however, for some chemicals the estimated cytotoxicity range is > 10 (see Supplemental Material, Table S1). Figure 3C shows the cumulative distribution of *in vitro* toxicodynamic variability factors across 149 chemicals in comparison to *in vivo* toxicodynamic variability factors across 34 chemicals (IPCS 2014). The distributions are strikingly similar, with medians equal to 3.04 (90% confidence intervals of 1.48–10.3) *in vitro* and 3.10 (1.70–38.5) *in vivo,* and not significantly different (Kolmogorov-Smirnov *p* = 0.548).

Next, we profiled the EC_10_ for each chemical by averaging within each population. Hierarchical clustering of these averaged profiles (Figure 3D) shows general assortment by ancestry, although variation was generally greater within than across populations. Although a large number of chemicals showed significant EC_10_ variation across populations or by sex (false discovery *q* < 0.05; see Supplemental Material, Table S3), this variation was modest; two examples are shown in Figure 3E.

*Heritability and mapping*. Trio-based analysis provided evidence of additive heritability for 17 chemicals (*q* < 0.2), with significant trio-based heritability estimates (*h*^2^) ranging from approximately 0.25 to approximately 0.5 (Figure 3F; results for all chemicals shown in Supplemental Material, Table S4). We augmented this analysis by essentially independent heritability estimation using GCTA (Yang et al. 2011) performed using the maximal set of 884 unrelated individuals. GCTA-based *h*^2^ ranged from approximately 0.4 to 0.8 for 34 significant chemicals (see Supplemental Material, Figure S3A,B). Correlation of these two heritability estimates was modest (Spearman *r* = 0.22, *p* = 0.0026) but highly consistent with simulations (average *r* = 0.24) as described in “Materials and Methods.”

Our use of EC_10_ values was motivated by relevance to human health assessment practices; however, elucidation of the underlying genetic mechanisms may be more powerful without assumptions about the point of departure. Moreover, EC_10_ is not sensitive to genetic influences apparent only at high concentrations. We thus adopted a three-stage approach to mapping, using 10 genotype principal components and sex as covariates. For the primary analysis, using the unrelated individuals, we applied the multivariate MAGWAS approach (Brown et al. 2012b), sensitive to any pattern of variation of cytotoxicity measurements due to genotype. Second, for the same individuals, we used EC_10_ values as a quantitative phenotype in regression analysis for an additive SNP model, using the larger set of 1.3 million SNPs (chr1-X). For individual SNPs, this analysis identified associations that might have been missed by MAGWAS and allowed us to investigate pathway-based associations (Schaid et al. 2012). Finally, to capture a larger number of SNPs and variants with lower MAF (Gamazon et al. 2012), we applied the EC_10_ regression approach to 690 of the unrelated individuals who were among 1000 Genomes Phase I, and used approximately 12.4 million variants with MAF ≥ 0.01. Preliminary analysis indicated phenotype outlier effects causing spurious significant findings due to the lower MAF threshold; after applying an initial filter of association *p* < 5 × 10^–8^, we recomputed the chemical × SNP analyses after applying an inverse quantile normalization to EC_10_.

We deemed each chemical worthy of separate investigation and applied per-chemical false discovery control, following proposals that SNPs with false discovery rates *q* < 0.10 be declared significant (van den Oord and Sullivan 2003). Table 1 shows these 48 chemical–SNP associations, after removing redundant regional findings within ± 1 Mb. The nearest gene is reported, along with partial *R*^2^, the portion of variance explained by MAGWAS across the concentrations after considering covariates. The most significant MAGWAS findings tend to have larger partial *R*^2^ (see Supplemental Material, Figure S4).

**Table 1:** MAGWAS multivariate association results.

Chemical^*a*^	CAS No.	SNP	Position (bp)^*b*^	Chromosome	Gene	*p*-Value^*c*^	*q*-Value^*d*^	Explained *R*^2^^*e*^
2-Amino-4-methylphenol	95-84-1	rs13120371	139092719	4	*SLC7A11*	8.42 × 10^–10^	0.0006	0.0723
Methyl mercuric (II) chloride	115-09-3	rs13120371	139092719	4	*SLC7A11*	8.89 × 10^–8^	0.0632	0.0414
*N*-Methyl-*p*-aminophenol sulfate	55-55-0	rs13120371	139092719	4	*SLC7A11*	4.88 × 10^–8^	0.0347	0.0395
*N*-Isopropyl-*N*’-phenyl-*p*-phenylenediamine	101-72-4	rs1159874	19916619	7	*TMEM196*	2.71 × 10^–9^	0.0019	0.0264
		rs6430301	148953669	2	*MBD5*	2.84 × 10^–7^	0.0674	0.0262
		rs3935192	75878841	17	*FLJ45079*	5.44 × 10^–7^	0.0968	0.0281
2-Amino-4-methylphenol	95-84-1	rs57046479	99635548	9	*ZNF782*	3.25 × 10^–7^	0.0769	0.0181
		rs6446632	4355380	4	*ZBTB49*	6.15 × 10^–7^	0.0875	0.0340
*o*-Aminophenol	95-55-6	rs1800566	69745145	16	*NFAT5*	4.32 × 10^–9^	0.0031	0.0554
		rs4244032	142794725	5	*NR3C1*	3.79 × 10^–7^	0.0430	0.0210
		rs8073076	63454129	17	*AXIN2*	1.10 × 10^–6^	0.0784	0.0193
		rs11062381	2954423	12	*FKBP4*	1.46 × 10^–6^	0.0945	0.0337
Titanocene dichloride	1271-19-8	rs62009303	92805261	15	*SLCO3A1*	1.97 × 10^–8^	0.0140	0.0222
		rs62189869	162922728	2	*LOC151171*	1.82 × 10^–7^	0.0431	0.0197
		rs12902246	49274274	15	*SECISBP2L*	4.62 × 10^–7^	0.0657	0.0311
		rs1906308	104333651	11	*PDGFD*	7.90 × 10^–7^	0.0703	0.0261
13-*cis*-Retinal	472-86-6	rs541217	106564400	6	*PRDM1*	1.23 × 10^–8^	0.0087	0.0205
		rs4532252	12397379	4	*RAB28*	3.72 × 10^–7^	0.0715	0.0329
*N*,*N*-Diethyl-*p*-phenylenediamine	93-05-0	rs6691053	173868955	1	*DARS2*	2.82 × 10^–8^	0.0200	0.0194
		rs61879371	19852683	11	*NAV2*	1.39 × 10^–7^	0.0494	0.0181
2,4-Decadienal	25152-84-5	rs1194596	154238383	1	*C1orf43*	3.60 × 10^–8^	0.0207	0.0282
		rs4689451	6458552	4	*PPP2R2C*	9.97 × 10^–8^	0.0236	0.0211
Malachite green oxalate	2437-29-8	rs3742522	24906534	14	*KHNYN*	5.53 × 10^–8^	0.0062	0.0388
		rs10772306	10677140	12	*KLRAP1*	3.59 × 10^–7^	0.0283	0.0169
		rs717818	141830833	4	*RNF150*	1.40 × 10^–6^	0.0908	0.0180
Fumaronitrile	764-42-1	rs11048994	27530778	12	*ARNTL2*	7.08 × 10^–8^	0.0504	0.0136
		rs12962668	444687	18	*COLEC12*	2.64 × 10^–7^	0.0940	0.0262
Retinal	116-31-4	rs11590090	113313563	1	*FAM19A3*	9.91 × 10^–8^	0.0508	0.0198
		rs34835780	3842112	1	*LOC100133612*	2.14 × 10^–7^	0.0508	0.0143
Permethrin	52645-53-1	rs2408151	5912100	8	*MCPH1*	1.04 × 10^–7^	0.0740	0.0211
		rs2598	47241618	20	*PREX1*	2.26 × 10^–7^	0.0805	0.0197
1,3-Dicyclohexylcarbodiimide	538-75-0	rs28437300	22224506	8	*SLC39A14*	4.25 × 10^–9^	0.0030	0.0245
Dieldrin	60-57-1	rs504504	85420044	1	*MCOLN2*	1.64 × 10^–8^	0.0116	0.0517
Flutamide (pubertal study)	13311-84-7	rs17186961	103630028	8	*KLF10*	1.83 × 10^–8^	0.0130	0.0283
Aldrin	309-00-2	rs340251	158599864	3	*MFSD1*	2.37 × 10^–8^	0.0118	0.0271
2,3,4,5-Tetrachlorophenol	4901-51-3	rs7879360	88236251	X	*CPXCR1*	3.95 × 10^–8^	0.0281	0.0181
Colchicine	64-86-8	rs7777880	48275852	7	*ABCA13*	4.34 × 10^–8^	0.0308	0.0244
Sulfathiazole	72-14-0	rs1796415	121543011	12	*P2RX7*	4.94 × 10^–8^	0.0351	0.0416
Reserpine	50-55-5	rs13143102	131264117	4	*C4orf33*	5.05 × 10^–8^	0.0359	0.0388
Dichlorvos (Vapona)	62-73-7	rs1037353	83525588	11	*DLG2*	6.73 × 10^–8^	0.0479	0.0165
1,2-Epoxy-3-chloropropane	106-89-8	rs3130884	72228285	X	*PABPC1L2B*	6.97 × 10^–8^	0.0496	0.0182
Cycloheximide	66-81-9	rs8053118	79168698	16	*WWOX*	7.66 × 10^–8^	0.0545	0.0189
Benzethonium chloride	121-54-0	rs62496173	9309398	8	*TNKS*	9.07 × 10^–8^	0.0645	0.0220
Tetrachlorvinphos	961-11-5	rs7642013	32638632	3	*DYNC1LI1*	9.38 × 10^–8^	0.0667	0.0208
Mono(2-ethylhexyl)phthalate	4376-20-9	rs1204399	99886830	X	*TNMD*	9.79 × 10^–8^	0.0577	0.0285
7,12-Dimethylbenzanthracene	57-97-6	rs9932935	16247471	16	*ABCC1*	9.84 × 10^–8^	0.0700	0.0338
Phenylmercuric acetate	62-38-4	rs12899102	40495067	15	*BUB1B*	1.41 × 10^–7^	0.0522	0.0233
*o*-Phenanthroline	66-71-7	rs11716740	182831688	3	MCCC1	1.98 × 10^–7^	0.0740	0.0238
bp, base pair. ^***a***^The first three entries highlight that rs13120371 in *SLC7A11* was observed with false discovery rates *q* < 0.10 for these chemicals; remaining entries are sorted first by chemical, and then *p*-value. ^***b***^NCBI_build_37. ^***c***^MAGWAS *p*-value. ^***d***^False discovery rate *q*-value obtained per chemical using ~ 700K SNPs by MAGWAS. ^***e***^Partial *R*^2^ attributable to variation in genotype.								

Table 1 shows data for each chemical, but a re-ranking by *p*-values revealed that the top 10 significant associations includes three solute carriers (*SLC7A11* for 2-amino-4-methylphenol, *SLC39A14* for 1,3-dicyclohexylcarbodiimide, and *SLCO3A1* for titanocene dichloride), the transmembrane protein *TMEM196* for *N*-isopropyl-*N*´-phenyl-*p*-phenylenediamine, and *NFAT5,* which activates several solute carriers in response to osmotic stress, for *o*-aminophenol. Our findings suggest a major role for membrane proteins and solute carrier transporters in mediating cytotoxicity, as has been reported for the chemotherapeutic agent paclitaxel (Njiaju et al. 2012).

**Figure 4:**
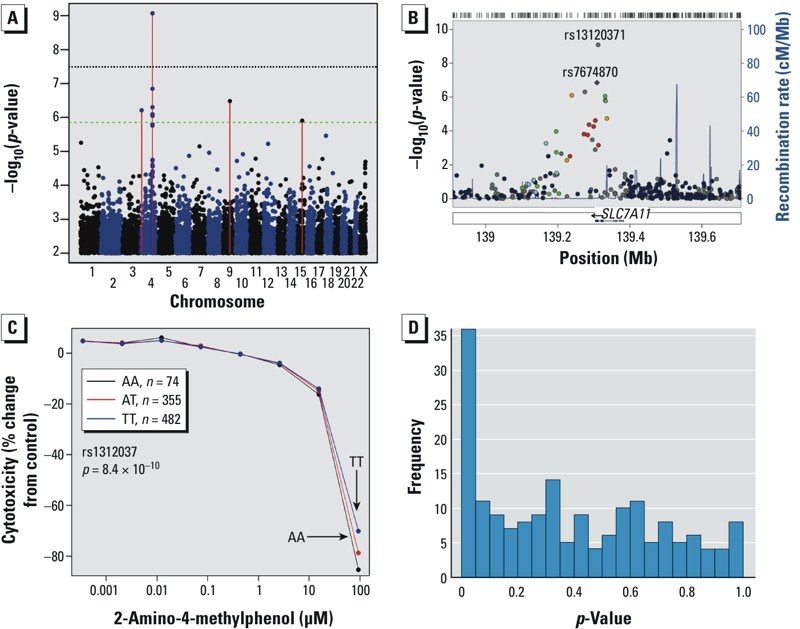
(*A*) Manhattan plot of MAGWAS –log_10_(*p*) versus genomic position for association of genotype and cytotoxicity to 2-amino-4-methylphenol. The green dashed line indicates the significance threshold for suggestive association (expected once per genome scan), and the black dotted line represents the Bonferroni-corrected significance for a single chemical. (*B*) LocusZoom plot of the most significant region. Abbreviations: cM, centimorgans; Mb, megabase pair. SNP rs13120371 was the most significant (*p* = 8.4 × 10^–10^), and the nearby rs7674870 was used for comparison of linkage disequilibrium patterns in the region. See Supplemental Material, Figure S5, for color heat maps of the significance association of the individual SNPs. (*C*) Average concentration–response profiles of cytotoxicity of 2-amino-4-methylphenol plotted separately for each rs13120371 genotype (AA, AT, and TT); genotype effects were observed only for the highest concentrations. (*D*) Histogram of EC_10_-based *p*-values for all 179 chemicals for rs13120371, showing an excess of small *p*-values.

The most significant MAGWAS association (*p* = 8.4 × 10^–10^) was 2-amino-4-methylphenol at rs13120371 in the 3´ UTR of *SLC7A11*, a cystine and glutamate transporter. The result was highly significant on a per-chemical basis (*q* = 0.0006), and at the significance threshold for the entire combined set of SNPs × chemicals (*q* = 0.10). Figure 4A,B shows the corresponding Manhattan and regional plots. Same exact SNP also appeared with *q* < 0.10 for methyl mercuric chloride and *N*-methyl-*p*-aminophenol sulfate (Table 1). Comparative curves show that the differences in cytotoxic response appear mainly at the highest concentration (Figure 4C). The plot illustrates the contrast between EC_10_, which did not differ significantly by genotype, and the multivariate MAGWAS finding, which is sensitive to concentration–response variation.

Supplemental Material, Table S5 shows results from the EC_10_ regression analyses, with all significant findings (per-chemical *q* < 0.10) shown after removing redundant regional findings (63 unique chemicals, 260 unique nearest gene assignments). For many chemicals, we observed the effects of genotype both for EC_10_ and across the multivariate response, and the two approaches provided similar evidence (see Supplemental Material, Figure S5). At the false discovery rate of < 0.1, only approximately 18 unique chemicals would be expected to appear in Supplemental Material, Table S5. SNPs in four genes appear for three or more chemicals: *GRIP1* (glutamate receptor interacting protein 1), which directs localization of transmembrane proteins; *FMN2*, a component of p21-based cell cycle arrest; *DNER*, a transmembrane protein associated with glioblastoma propagation; and the cell membrane cadherin *CDH13*, an epithelial tumor suppressor. As we observed with MAGWAS analysis, membrane-localized proteins appear to play an important role. Because EC_10_ values were available for 179 chemicals, we found that GCTA-based heritability estimates are largely reflected in a tendency toward small *p*-values, a phenomenon that is difficult to discern for single-trait GWAS studies (see Supplemental Material, Figure S3C). Supplemental Material, Table S6 shows the significant associations for the analysis of the larger number (12.4 million) of sequenced SNPs.

For rs13120371 in *SLC7A11*, we hypothesized that the SNP may modify resistance to a larger number of chemicals. We examined the EC_10_ *p*-values for rs13120371 across all 179 chemicals and observed a clear excess of small *p*-values (Figure 4D). Using a standard false-discovery computation, we estimated the proportion of true discoveries for the SNP across the chemicals as 0.25, a significant trend that remained even after removal of the three top MAGWAS-identified chemicals. The estimated number of true discoveries, corresponding to an estimated 44 chemicals showing true cytotoxicity association with rs13120371, is subject to considerable sampling variation. Nonetheless, the data indicate that *SLC7A11* may be a cytotoxicity mediator, and a role for *SLC7A11* has been proposed in glutathione-mediated chemotherapeutic resistance (Huang et al. 2005).

We performed “pathway” association analysis of gene sets/ontologies for EC_10_ phenotypes and the 1.3-million Omni 2.5 SNPs using *gene set scan* (Schaid et al. 2012) which computes significance of SNPs, genes, and ontologies. Eleven chemicals had significant pathways, and several chemicals showed significant associations with immune-response pathways and ontologies (see Supplemental Material, Table S7) at a family-wise error rate of < 0.05.

## Discussion

Despite early concerns over the ability to map meaningful response traits in LCLs and questions about this model’s relevance to toxicity studies of chemicals that require metabolism, our results suggest that large sample sizes—on the order necessary for mapping human complex traits (Goldstein 2009)—can overcome challenges. Importantly, we have demonstrated the feasibility of using an *in vitro* population-based model system for assessment of individual variability in next-generation risk assessment (Zeise et al. 2013). Although here we present our results as a survey, results for each chemical screened will be useful for future targeted investigations. Moreover, use of a common protocol enables valid comparisons across chemicals that are difficult to perform across individual studies.

Quantitative high-throughput screening of a large number of compounds affords detailed investigation of concentration response, which is critical for safety margins and informed decisions on relative hazard ranking/prioritization. Most similar *in vitro* studies have characterized the concentration effect through EC_50_ (Neubig et al. 2003); however, there are many limitations of this approach for screening data (Sand et al. 2012). Here, we derived EC_10_ or no-effect values to describe variability across cell lines and among chemicals, and for GWAS analyses. In addition, we used the full complement of the concentration–response values for multivariate analysis.

To date, high-throughput screening for chemical prioritization has been largely limited to small numbers of genetic variants, and to models that are limited in diversity. Although cytotoxicity in LCLs is just one among multiple measures of toxicity, the availability of > 1,000 samples from global populations allows for precise estimation of population response range, filling a critical need (Collins et al. 2008). Thus, prioritization may be based on central tendency (e.g., median) or sensitive subpopulation (population quantile) estimates of activity, depending on contextual suitability.

The data generated using this approach also may help refine risk assessment (Zeise et al. 2013), potentially providing the basis for chemical-specific factors for toxicodynamic variability, replacing the canonical 10^½^ uncertainty/assessment factor. Cytotoxicity is often considered a crude measure, but for most chemicals evaluated in the ToxCast program, it constitutes a large proportion of “signal” detected in various high-throughput assays. Therefore, cytotoxicity may often be an appropriate surrogate for systemic toxicity.

We also compared our results on interindividual variability to those collected from human studies (IPCS 2014). Although data on *in vivo* human toxicodynamic variability are limited, we found that they appear largely consistent with our *in vitro* estimates. Interestingly, both *in vivo* and *in vitro* data suggest that the usual 10^½^ factor is appropriate “on average,” but for roughly half of the chemicals the estimated factor would be greater. An estimate of the extent of overall human variability would also necessitate incorporating toxicokinetic variability (Judson et al. 2011).

Beyond immediate utility of our data in health assessments, we observed in GWAS analyses that genes with protein localization to cell membranes, including solute carriers, are enriched. Solute carrier transporters have been investigated as potential mediators of cytotoxicity for chemotherapeutics (DeGorter et al. 2012; Njiaju et al. 2012), controlling cellular influx and efflux of drugs/toxicants. Moreover, several families of solute carriers are important toxicity mediators in liver and kidney (DeGorter et al. 2012). To our knowledge, we are the first to highlight the role of membrane transporters in interindividual susceptibility to a wide range of environmental chemicals, beyond chemotherapeutic agents.

The results for rs13120371 in *SLC7A11* were striking, and are supported by growing literature on its importance in chemoresistance (Lo et al. 2008). Small interfering *SLC7A11* RNA increased sensitivity to various agents in cancer cell lines (Pham et al. 2010). Expression was altered in drug-resistant ovarian cancer cell lines (Januchowski et al. 2013), was downregulated in response to thymoquinone in breast cancer cells (Motaghed et al. 2014), and predicted poor survival *in vivo* (Kinoshita et al. 2013). In addition, *SLC7A11* was inversely correlated with clinical outcome in bladder cancer and negatively regulated by a microRNA for cisplatin-resistant cells (Drayton et al. 2014).

## Conclusions

Although the risk assessment process is shifting toward greater reliance on *in vitro* data, none of the *in vitro* assays in Tox21, ToxCast, or other large-scale screening programs is designed to address individual variability (Rusyn and Daston 2010). The present study demonstrates how a large-scale systems biology experiment (toxicity phenotyping and genetic mapping) can aid translation to public health protection, and provides novel information about global interindividual variability. The availability of genetically diverse, genetically defined renewable human cell lines opens an opportunity for *in vitro* toxicity testing at the population scale. Our heritability estimates show that genetic variation may have a profound effect on differences between cell lines and can be quantified and used to generate testable hypotheses about mechanisms of toxicity.

## Supplemental Material

(2.5 MB) PDF
