# Supplementary material for: Population-Based in Vitro Hazard and Concentration–Response Assessment of Chemicals: The 1000 Genomes High-Throughput Screening Study
Source: Environ Health Perspect. 2015 Jan 13;123(5):458–66. doi: 10.1289/ehp.1408775 (PMC4421772; doi:10.1289/ehp.1408775)
Supplement: (2.5 MB) PDF [file ehp.1408775.s001.508.pdf]

Environmental Health Perspectives doi:10.1289/ehp.1408775

Supplemental Material

Population-Based *in Vitro* Hazard and Concentration–Response Assessment of Chemicals: The 1000 Genomes High-Throughput Screening Study

Nour Abdo, Menghang Xia, Chad C. Brown, Oksana Kosyk, Ruili Huang, Srilatha Sakamuru, Yi-Hui Zhou, John Jack, Paul Gallins, Kai Xia, Yun Li, Weihsueh A. Chiu, Alison Motsinger-Reif, Christopher P. Austin, Raymond R. Tice, Ivan Rusyn, and Fred A. Wright

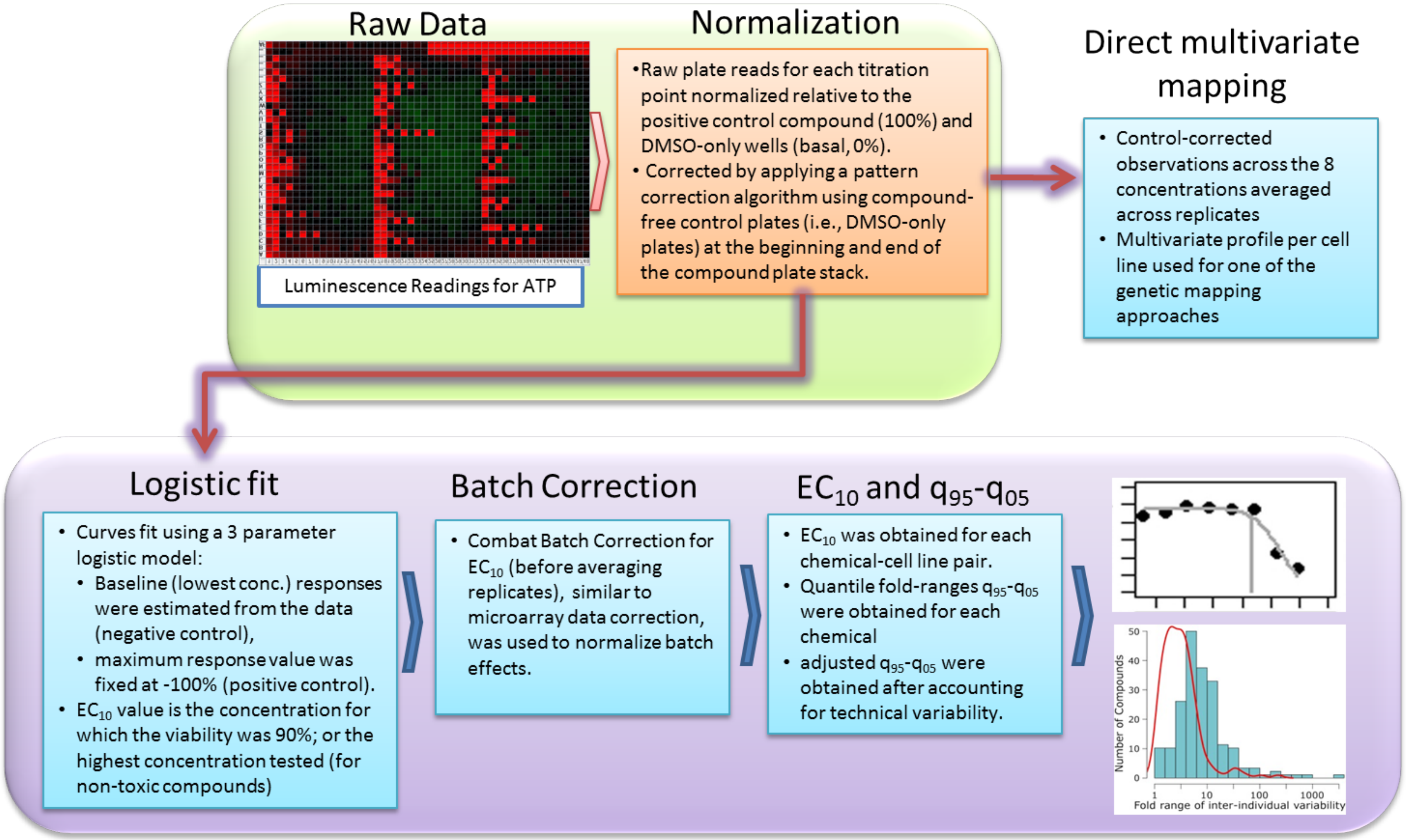

Figure S1. Flow chart of data processing to obtain cytotoxicity response values and EC_{10}.

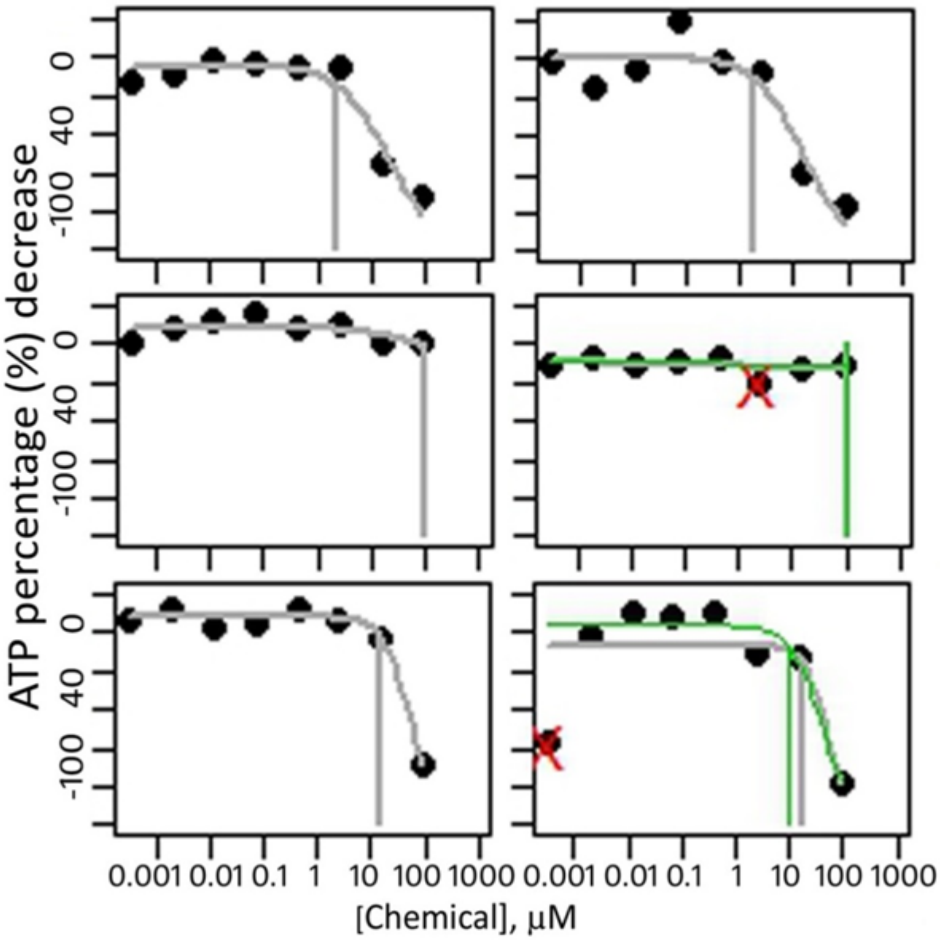

Figure S2. Illustrative fits for cytotoxicity estimation, with fits and EC_{10} point estimate (vertical lines) shown in grey. The top and bottom rows show instances of cytotoxic compounds. The middle panels show compounds that are non-cytotoxic for the range of concentrations, and EC_{10} was fixed at the maximum concentration. Points marked in "X" were excluded on the basis of the likelihood ratio criterion described in Online Methods, providing new fits and EC_{10} values shown in green.

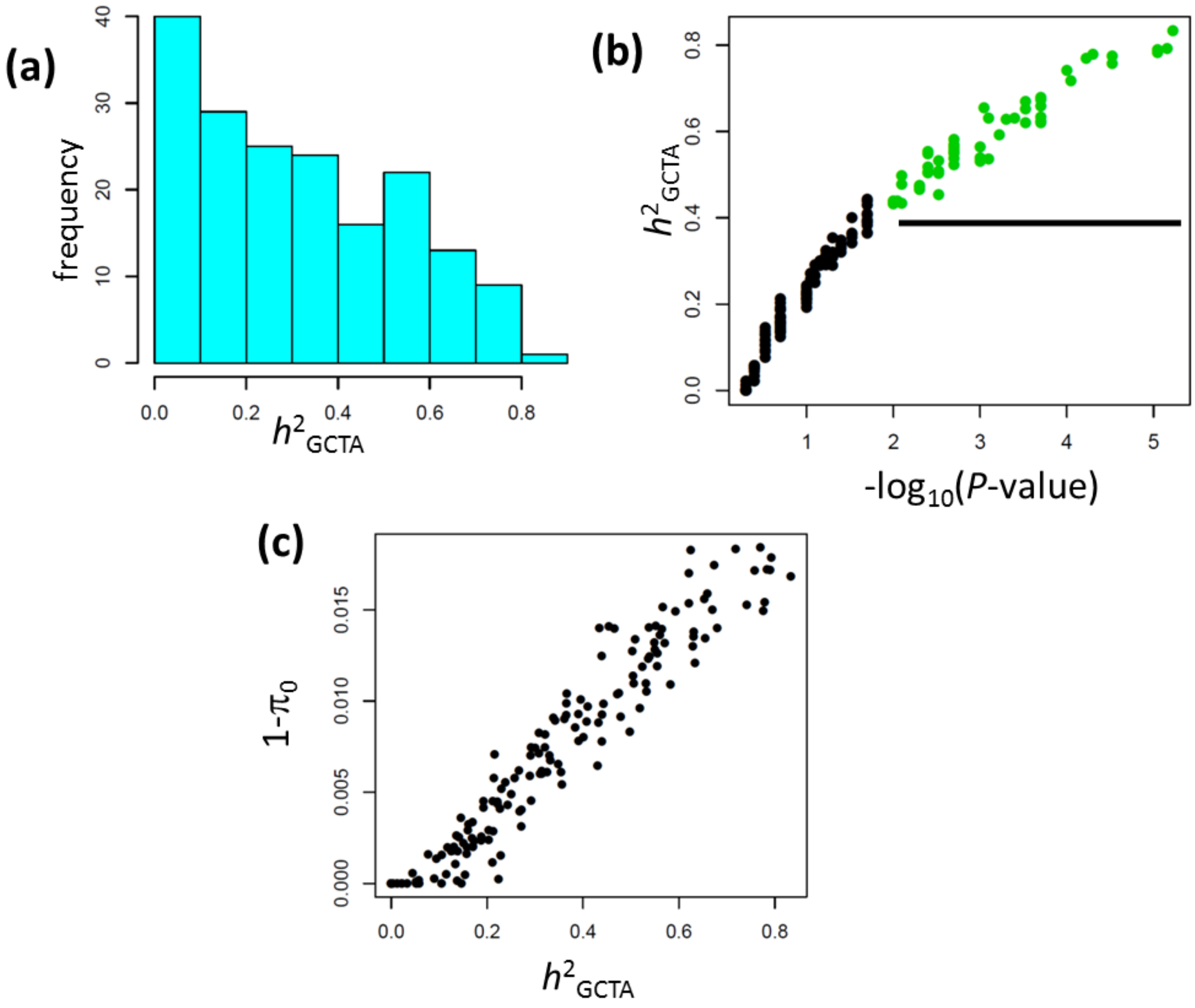

Figure S3. (a) Histogram of GCTA heritability (h2) estimates for EC10. **(b)** Heritability estimates for each chemical vs. GCTA -log10(P-values). For GCTA heritability, 34 chemicals with false discovery rate q<0.05 are shown in green and marked with a line. **(c)** The estimated proportion of true discoveries (among 1.3M SNPs) vs. GCTA heritability estimates for EC10 (r=0.96).

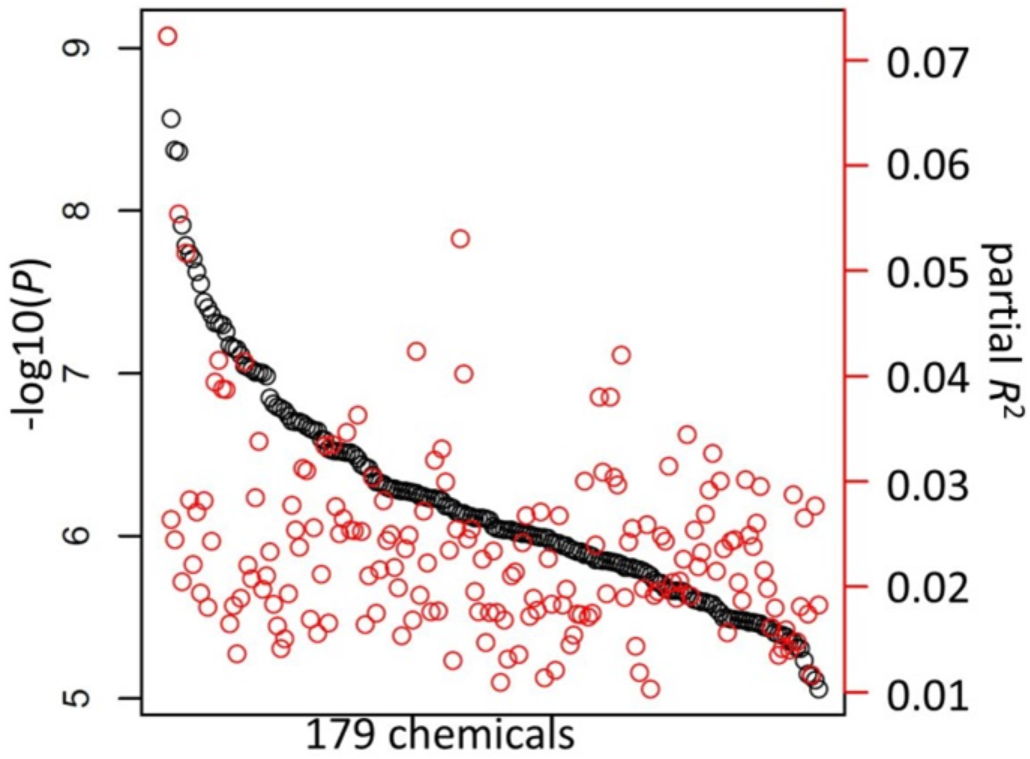

Figure S4. A plot of the most significant genetic variant for each chemical, with black dots depicting the -log10 p-values for the association, and red dots showing the maximum partial R-squared across all 8 concentrations.

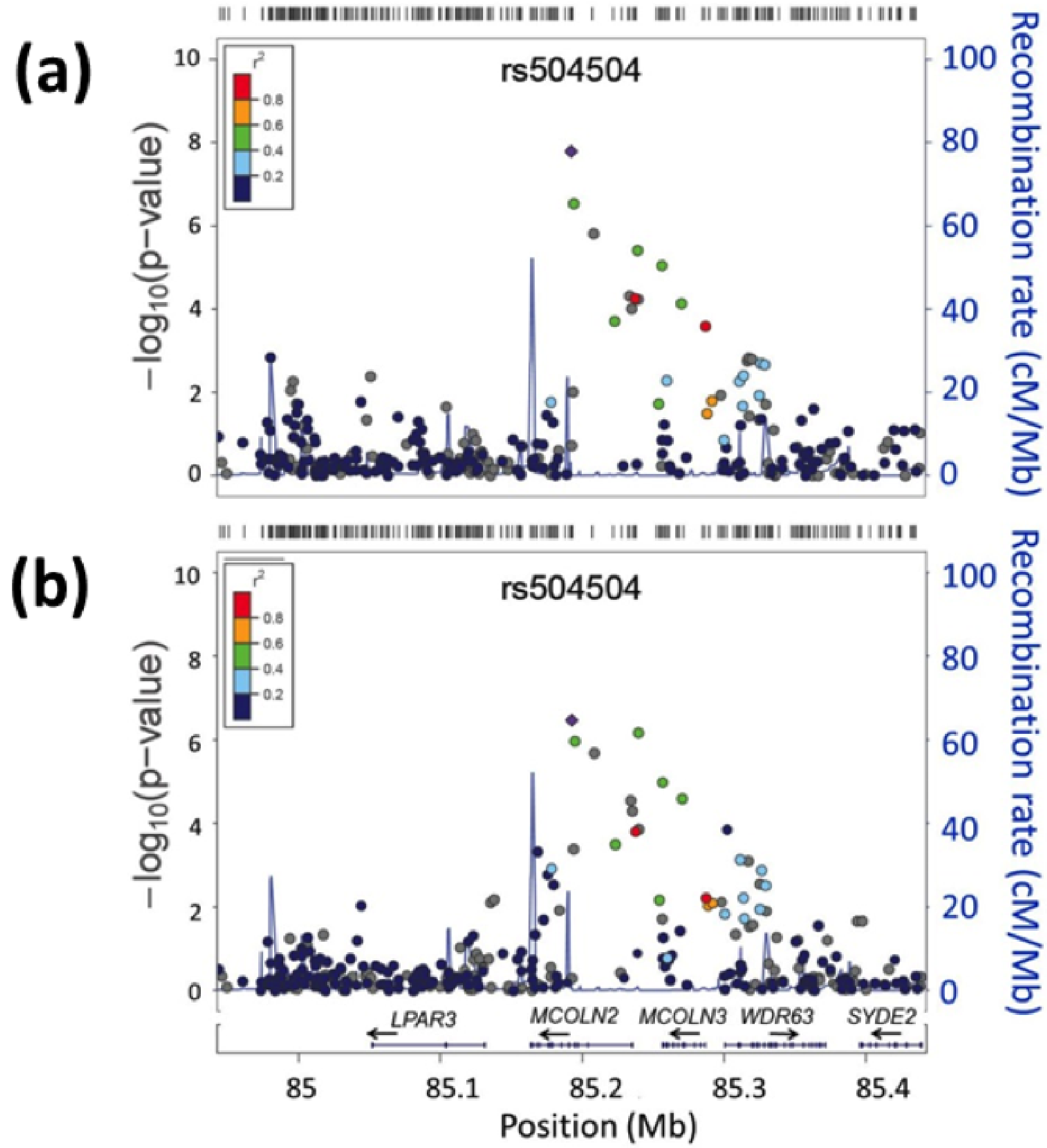

Figure S5. LocusZoom plots of the most significant SNP (rs504504, chr1p22) associated with cytotoxicity of dieldrin from either **(a)** MAGWAS, or **(b)** PLINK regression analyses, using EC_{10} as a quantitative phenotype. Color of each SNP signifies the linkage disequilibrium value depicted in a heat map inset in each figure.

Table S1. Population-wide cytotoxicity phenotypes for each screened compound.

Chemical_Name	CAS#	Mean[a]	Median[b]	SD	q01[c]	q05	q50	q95	q99	Fold Range q50-q01	Fold Range q50-q05	VIF[d]	Shrunken Fold Range q50-q01	Shrunken Fold Range q50-q05
Daunomycin HCL	23541-50-6	-2.47	-2.56	0.32	-3.07	-2.86	-2.61	-1.83	-1.69	2.88	1.76	4.14	1.68	1.32
Colchicine	64-86-8	-2.23	-2.21	0.21	-2.82	-2.66	-2.21	-1.92	-1.87	4.05	2.83	-25.1	N/A	N/A
Colchicine	64-86-8	-2.23	-2.20	0.21	-2.84	-2.71	-2.16	-1.91	-1.77	4.77	3.55	5.27	1.97	1.74
Malachite green oxalate	2437-29-8	-1.49	-1.47	0.24	-2.22	-1.91	-1.49	-1.10	-0.98	5.33	2.65	5.35	2.06	1.52
Cycloheximide	66-81-9	-1.35	-1.36	0.40	-2.19	-1.96	-1.35	-0.87	-0.65	7.02	4.12	6.45	2.15	1.75
Tetramethylthiouram disulfide	137-26-8	-1.36	-1.36	0.29	-2.32	-1.98	-1.34	-0.78	-0.23	9.50	4.29	2.39	4.29	2.56
Digoxin	20830-75-5	-1.30	-1.30	0.22	-2.04	-1.69	-1.27	-0.96	-0.59	5.80	2.58	5.08	2.18	1.52
Ziram	137-30-4	-1.26	-1.30	0.56	-2.40	-2.14	-1.27	-0.28	0.50	13.58	7.52	1.82	6.92	4.46
Cycloheximide	66-81-9	-1.21	-1.20	0.29	-2.01	-1.75	-1.21	-0.69	-0.51	6.26	3.44	3.10	2.83	2.02
Phenylmercuric acetate	62-38-4	-1.18	-1.20	0.35	-2.04	-1.76	-1.20	-0.61	-0.36	7.00	3.62	2.03	3.92	2.46
Zinc pyrithione	13463-41-7	-1.10	-1.14	0.46	-2.06	-1.82	-1.16	-0.31	0.14	8.12	4.57	1.91	4.56	3.00
Mercuric chloride	7487-94-7	-0.95	-1.02	0.68	-2.26	-1.98	-1.07	0.26	0.60	15.62	8.23	1.87	7.46	4.67
6-Thioguanine (6-TG)	154-42-7	-0.80	-0.86	0.79	-2.43	-2.00	-0.88	0.55	0.99	35.27	13.17	2.00	12.4	6.20
Hexamethyl-p-rosaniline chloride	548-62-9	-0.77	-0.75	0.26	-1.44	-1.29	-0.73	-0.41	-0.21	5.12	3.59	2.80	2.65	2.15
Methyl mercuric (II) chloride	115-09-3	-0.55	-0.54	0.37	-1.53	-1.25	-0.55	0.06	0.24	9.70	5.10	1.87	5.26	3.29
6-Mercaptopurine monohydrate	6112-76-1	-0.35	-0.52	0.97	-2.09	-1.70	-0.51	1.72	2.09	37.58	15.26	2.15	11.9	6.43
Chloranil	118-75-2	-0.15	-0.19	0.61	-1.08	-0.79	-0.16	0.75	1.16	8.45	4.32	3.68	3.04	2.14
Azathioprine	446-86-6	-0.02	-0.19	0.83	-1.64	-1.27	-0.15	1.40	1.69	30.62	13.05	1.79	12.9	6.83
5-Fluorouracil	51-21-8	-0.12	-0.14	0.44	-1.61	-1.14	-0.15	0.98	1.29	28.62	9.82	2.20	9.60	4.67
tetra-N-Octylammonium bromide	14866-33-2	-0.13	-0.11	0.29	-1.04	-0.66	-0.12	0.35	0.52	8.34	3.44	2.13	4.28	2.33
Ethidium bromide	1239-45-8	0.02	-0.01	0.32	-0.76	-0.54	-0.01	0.57	0.97	5.69	3.39	1.87	3.56	2.44
p-Nitrosodiphenylamine	156-10-5	0.02	0.05	0.34	-0.86	-0.58	0.00	0.53	0.74	7.22	3.78	1.70	4.56	2.78
N,N-Dimethyl-p-nitrosoaniline	138-89-6	0.02	0.06	0.33	-0.98	-0.65	0.04	0.52	0.78	10.71	4.90	2.66	4.28	2.65
Cetylpyridinium bromide	140-72-7	0.08	0.07	0.33	-0.73	-0.47	0.06	0.67	0.87	6.23	3.43	1.80	3.92	2.51
Nitrogen mustard hydrochloride	55-86-7	0.11	0.11	0.35	-0.93	-0.69	0.10	0.88	1.06	10.82	6.21	1.83	5.83	3.86
Sodium dichromate dihydrate (VI)	7789-12-0	0.11	0.11	0.51	-0.81	-0.51	0.11	0.69	0.98	8.37	4.14	2.74	3.61	2.36
Pentaerythritol triacrylate	3524-68-3	0.10	0.15	0.30	-0.68	-0.47	0.15	0.53	0.76	6.75	4.11	2.72	3.18	2.36
Potassium dichromate	7778-50-9	0.16	0.17	0.32	-0.68	-0.40	0.16	0.69	0.87	6.84	3.63	2.40	3.46	2.30
Pyrimethamine	58-14-0	0.19	0.17	0.57	-1.33	-0.78	0.19	1.23	1.52	33.41	9.28	3.52	6.49	3.28
Sodium dichromate dihydrate (VI)	7789-12-0	0.18	0.20	0.37	-0.72	-0.45	0.19	0.79	1.01	8.11	4.40	2.14	4.19	2.75
2-Octyl-3-isothiazolone	26530-20-1	0.20	0.23	0.29	-0.68	-0.35	0.22	0.64	0.92	7.98	3.77	2.73	3.51	2.23
Chlorambucil	305-03-3	0.27	0.28	0.28	-0.54	-0.25	0.28	0.72	0.92	6.68	3.40	3.25	2.87	1.97
Diglycidyl resorcinol ether	101-90-6	0.30	0.31	0.36	-0.65	-0.34	0.32	0.89	1.12	9.28	4.62	2.26	4.40	2.77
9-Aminoacridine, monohydrochloride	52417-22-8	0.33	0.33	0.25	-0.27	-0.10	0.32	0.75	1.08	3.92	2.65	2.18	2.52	1.94
1,3,5-Triglycidyl isocyanurate	2451-62-9	0.32	0.34	0.33	-0.59	-0.27	0.36	0.86	1.14	8.84	4.25	2.36	4.13	2.56
Fumaronitrile	764-42-1	0.41	0.37	0.33	-0.42	-0.11	0.36	1.08	1.53	6.09	2.95	4.65	2.31	1.65
o-Phenanthroline	66-71-7	0.37	0.37	0.20	-0.16	0.00	0.37	0.76	0.92	3.43	2.38	4.50	1.79	1.50
N,N'-Di-sec-butyl-p-phenyldiamine	101-96-2	0.38	0.39	0.21	-0.26	0.01	0.38	0.76	0.98	4.33	2.35	1.81	2.97	1.89

Chemical_Name	CAS#	Mean[a]	Median[b]	SD	q01[c]	q05	q50	q95	q99	Fold Range q50-q01	Fold Range q50-q05	VIF[d]	Shrunken Fold Range q50-q01	Shrunken Fold Range q50-q05
Hexachlorophene	70-30-4	0.39	0.40	0.29	-0.43	-0.12	0.40	0.85	1.03	6.76	3.35	2.54	3.31	2.14
Tetraethylene glycol diacrylate	17831-71-9	0.39	0.43	0.35	-0.42	-0.07	0.43	0.87	0.99	7.22	3.19	3.89	2.72	1.80
Chlorhexidine	55-56-1	0.44	0.45	0.28	-0.63	-0.21	0.45	0.97	1.18	11.86	4.58	2.91	4.26	2.44
Methylene bis(thiocyanate)	6317-18-6	0.49	0.47	0.26	-0.34	0.05	0.46	0.96	1.14	6.37	2.57	2.04	3.65	1.93
beta-Nitrostyrene	5153-67-3	0.48	0.49	0.30	-0.20	0.12	0.49	1.08	1.30	4.95	2.36	3.19	2.45	1.62
Catechol	120-80-9	0.54	0.51	0.27	-0.40	-0.05	0.50	0.97	1.15	8.10	3.62	2.20	4.09	2.38
Benzethonium chloride	121-54-0	0.55	0.54	0.17	0.14	0.26	0.54	0.86	0.98	2.53	1.93	3.32	1.66	1.44
N-Methyl-p-aminophenol sulfate	55-55-0	0.58	0.59	0.26	-0.14	0.12	0.58	0.99	1.19	5.32	2.95	2.08	3.19	2.12
Domiphen bromide	538-71-6	0.62	0.62	0.36	-0.04	0.20	0.63	0.96	1.08	4.66	2.70	2.47	2.66	1.88
Ergotamine tartrate	379-79-3	0.61	0.63	0.24	-0.58	-0.08	0.63	1.24	1.51	16.30	5.17	5.48	3.30	2.02
1,1,1,2-Tetrabromoethane	630-16-0	0.61	0.65	0.27	-0.22	0.07	0.64	0.98	1.14	7.35	3.75	2.10	3.96	2.49
2,2',4'-Trichloroacetophenone	4252-78-2	0.70	0.71	0.28	0.01	0.30	0.70	1.10	1.32	4.92	2.51	31.3	1.33	1.18
Nitazoxanide	55981-09-4	0.70	0.71	0.24	-0.10	0.22	0.71	1.16	1.35	6.44	3.05	2.68	3.12	1.98
2,4-Difluoronitrobenzene	446-35-5	0.70	0.72	0.68	-1.22	-0.67	0.72	1.87	2.06	86.49	24.27	3.40	11.2	5.64
Iodochlorohydroxyquinoline	130-26-7	0.74	0.76	0.25	-0.09	0.28	0.75	1.14	1.28	7.00	2.94	3.41	2.87	1.79
Diethylene glycol diacrylate	4074-88-8	0.74	0.77	0.28	-0.29	0.18	0.78	1.13	1.34	11.70	3.98	1.95	5.81	2.69
2-Amino-4-chlorophenol	95-85-2	0.75	0.78	0.53	-0.82	-0.30	0.80	1.63	1.87	40.89	12.36	3.89	6.56	3.58
Turmeric (>98% curcurmin)	458-37-7	0.79	0.80	0.21	0.08	0.40	0.80	1.10	1.29	5.34	2.53	4.73	2.16	1.53
Tetraethylene glycol diacrylate	17831-71-9	0.86	0.83	0.32	0.19	0.45	0.82	1.17	1.40	4.30	2.36	3.16	2.27	1.62
1-Methyl-3-nitro-1-nitroso-guanidine	70-25-7	0.82	0.83	0.21	-0.14	0.39	0.83	1.39	1.80	9.28	2.74	2.03	4.78	2.03
Dibromonitromethane	598-91-4	0.87	0.87	0.23	0.23	0.45	0.86	1.23	1.41	4.24	2.57	2.14	2.68	1.91
1,8-Dihydroxy-4,5-dinitroanthraquinone	81-55-0	0.83	0.87	0.22	0.25	0.42	0.87	1.18	1.28	4.16	2.79	2.35	2.54	1.95
1,6-Hexamethylene diacrylate	13048-33-4	0.79	0.90	0.97	0.26	0.56	0.92	1.13	1.27	4.58	2.31	12.3	1.54	1.27
4-Amino-4'-hydroxy-3-methyl-diphenylamine	6219-89-2	0.91	0.93	0.18	0.36	0.55	0.93	1.28	1.41	3.73	2.36	3.24	2.08	1.61
1,3-Diiminobenz (f)-isoindoline	65558-69-2	0.93	0.94	0.18	0.34	0.61	0.94	1.21	1.46	3.95	2.14	-8.49	N/A	N/A
1,3-Dicyclohexylcarbodiimide	538-75-0	0.93	0.94	0.21	0.28	0.52	0.95	1.44	1.67	4.71	2.70	2.30	2.78	1.92
Guggulsterones E	39025-24-6	0.96	0.95	0.26	-0.10	0.37	0.97	1.44	1.70	11.76	4.05	3.01	4.15	2.24
3-Chloro-4-(dichloromethyl)-5-hydroxy-2(5H)-furanone	77439-76-0	0.94	0.97	0.30	0.32	0.57	0.99	1.24	1.46	4.65	2.59	7.00	1.79	1.43
Dieldrin	60-57-1	0.96	0.98	0.32	0.17	0.48	0.99	1.54	1.77	6.58	3.20	2.09	3.68	2.23
17beta-Estradiol	50-28-2	0.98	0.98	0.33	-0.36	0.31	0.99	1.40	1.61	22.27	4.75	41.6	1.62	1.27
2,3,4,5-Tetrachloronitrobenzene	879-39-0	0.96	0.99	0.21	-0.35	0.33	1.00	1.47	1.68	22.39	4.68	8.70	2.87	1.69
Acetochlor	34256-82-1	0.95	0.99	0.33	0.22	0.63	1.01	1.36	1.61	6.10	2.38	-2.81	N/A	N/A
Aldrin	309-00-2	0.98	1.00	0.40	-0.30	0.27	1.01	1.66	1.96	20.55	5.44	4.39	4.23	2.24
p-Quinone	106-51-4	0.94	1.00	0.44	0.31	0.54	1.01	1.34	1.57	5.07	2.94	17.1	1.48	1.30
4,4-Thiobis(6-tert-butyl-m-cresol)	96-69-5	1.00	1.01	0.21	0.56	0.73	1.01	1.20	1.35	2.86	1.94	3.19	1.80	1.45
N-Isopropyl-N'-phenyl-p-phenylenediamine	101-72-4	1.00	1.01	0.14	0.25	0.63	1.02	1.25	1.38	5.81	2.43	11.7	1.67	1.30
Triamterene	396-01-0	0.99	1.01	0.36	0.22	0.46	1.02	1.73	1.87	6.26	3.64	1.81	3.91	2.61
2-Amino-4-methylphenol	95-84-1	0.97	1.02	0.29	-0.16	0.42	1.02	1.38	1.61	15.21	4.02	2.06	6.65	2.63
Dexamethazone	50-02-2	0.99	1.02	0.20	-1.63	-1.06	1.02	2.16	2.30	450.96	122.09	3.46	26.7	13.22

Chemical_Name	CAS#	Mean[a]	Median[b]	SD	q01[c]	q05	q50	q95	q99	Fold Range q50-q01	Fold Range q50-q05	VIF[d]	Shrunken Fold Range q50-q01	Shrunken Fold Range q50-q05
Hydroquinone	123-31-9	1.00	1.02	0.23	-0.22	0.31	1.03	1.60	1.87	18.05	5.25	1.85	8.41	3.39
Amiloride hydrochloride	2016-88-8	1.03	1.02	0.37	-0.59	0.25	1.05	1.66	1.89	43.86	6.25	20.7	2.30	1.50
t-Butylhydroquinone	1948-33-0	1.01	1.04	0.39	-0.51	-0.05	1.05	1.60	1.83	36.11	12.76	6.09	4.28	2.81
N,N,N',N'-Tetramethyl-p-phenylenediamine	100-22-1	1.06	1.06	0.16	-0.36	0.35	1.06	1.69	1.92	26.27	5.22	10.7	2.71	1.66
Tamoxifen citrate	54965-24-1	1.05	1.07	0.39	0.60	0.78	1.07	1.33	1.59	2.89	1.93	52.0	1.16	1.10
Hematoxylin	517-28-2	1.03	1.07	0.42	0.42	0.71	1.07	1.47	1.65	4.43	2.31	20.7	1.39	1.20
t-Butylhydroquinone	1948-33-0	1.05	1.07	0.25	0.17	0.61	1.07	1.46	1.72	7.97	2.90	2.51	3.70	1.96
Retinal	116-31-4	1.05	1.07	0.25	0.43	0.73	1.08	1.36	1.60	4.39	2.20	2.21	2.70	1.70
Aflatoxin B1 from Aspergillus flavus	1162-65-8	1.07	1.09	0.19	-0.77	0.30	1.09	1.76	1.91	72.47	6.18	3.25	10.7	2.74
p-tert-Butylcatechol	98-29-3	1.09	1.09	0.20	0.59	0.77	1.09	1.45	1.71	3.12	2.06	1.84	2.32	1.71
Reserpine	50-55-5	1.08	1.09	0.16	0.49	0.83	1.09	1.35	1.54	4.00	1.84	-15.1	N/A	N/A
Captan	133-06-2	1.06	1.09	0.51	0.39	0.73	1.09	1.40	1.62	4.98	2.32	4.04	2.22	1.52
o-Nitrobenzyl chloride	612-23-7	1.09	1.10	0.19	-0.78	0.02	1.10	1.92	2.12	75.75	11.77	5.18	6.69	2.95
Melatonin	73-31-4	1.11	1.12	0.33	0.03	0.56	1.12	1.63	1.84	12.31	3.63	1.70	6.86	2.69
Chlordecone (kepone)	143-50-0	1.12	1.13	0.12	0.68	0.92	1.13	1.29	1.41	2.79	1.59	-28.6	N/A	N/A
Captan	133-06-2	1.08	1.13	0.50	0.30	0.69	1.13	1.42	1.63	6.77	2.80	7.32	2.03	1.46
4-Chloro-o-phenylenediamine	95-83-0	1.13	1.14	0.17	-0.70	0.12	1.13	1.84	2.09	68.40	10.41	3.78	8.78	3.34
Saquinavir mesylate	149845-06-7	1.11	1.14	0.22	0.55	0.82	1.14	1.39	1.60	3.85	2.07	25.0	1.31	1.16
Progesterone	57-83-0	1.14	1.15	0.20	0.54	0.81	1.15	1.47	1.72	4.05	2.19	4.57	1.92	1.44
8-Hydroxyquinoline	148-24-3	1.12	1.16	0.38	0.32	0.57	1.16	1.63	1.73	6.88	3.85	1.94	4.00	2.63
7,12-Dimethylbenzanthracene	57-97-6	1.14	1.17	0.32	-0.57	0.35	1.17	1.70	1.89	54.35	6.54	57.5	1.69	1.28
N-(1,3-Dimethylbutyl)-N'-phenyl-p-phenylenediamine	793-24-8	1.17	1.17	0.13	0.69	0.97	1.17	1.36	1.57	3.04	1.58	4.49	1.69	1.24
Toxaphene	8001-35-2	1.14	1.17	0.29	0.84	0.97	1.17	1.44	1.61	2.14	1.59	2.88	1.56	1.31
Dazomet	533-74-4	1.19	1.18	0.13	0.50	0.70	1.18	1.70	1.89	4.74	3.02	1.60	3.42	2.40
HC blue 2	33229-34-4	1.16	1.18	0.26	-0.10	0.52	1.18	1.61	1.80	18.85	4.52	-13.3	N/A	N/A
Titanocene dichloride	1271-19-8	1.15	1.19	0.35	0.30	0.68	1.18	1.56	1.78	7.61	3.20	3.69	2.87	1.83
p-Aminophenol	123-30-8	1.20	1.20	0.17	0.71	0.97	1.19	1.55	1.75	3.03	1.69	-17.9	N/A	N/A
Ethacrynic acid	58-54-8	1.21	1.20	0.19	0.50	0.83	1.20	1.63	1.90	5.00	2.32	5.80	1.95	1.42
Dibromoacetonitrile	3252-43-5	1.20	1.20	0.30	0.68	0.86	1.20	1.64	1.79	3.30	2.17	3.41	1.91	1.52
Progesterone	57-83-0	1.19	1.20	0.29	0.61	0.89	1.20	1.51	1.74	3.89	2.03	4.84	1.85	1.38
Chlordane (technical grade)	12789-03-6	1.20	1.20	0.19	-0.48	0.52	1.20	1.62	1.83	47.66	4.82	10.5	3.30	1.63
Amitriptyline HCl	549-18-8	1.21	1.21	0.22	0.59	0.88	1.20	1.52	1.73	4.11	2.12	10.4	1.55	1.26
2,2'-Thiobis(4,6-dichlorophenol)	97-18-7	1.22	1.21	0.22	0.61	0.77	1.20	1.64	1.75	3.89	2.70	4.18	1.94	1.62
Dichloroacetonitrile	3018-12-0	1.21	1.21	0.24	-0.01	0.68	1.21	1.66	1.82	16.38	3.40	3.67	4.30	1.89
N-(1-Naphthyl)ethylenediamine dihydrochloride	1465-25-4	1.23	1.22	0.11	0.96	1.06	1.22	1.46	1.63	1.83	1.45	3.05	1.41	1.24
N,N'-Diphenyl-p-phenylenediamine	74-31-7	1.21	1.22	0.24	-0.32	0.49	1.22	1.78	1.94	35.03	5.36	-35.6	N/A	N/A
Endosulfan	115-29-7	1.20	1.22	0.35	0.26	0.85	1.23	1.59	1.70	9.22	2.40	3.11	3.52	1.64
Tetrachlorvinphos	961-11-5	1.22	1.23	0.28	0.19	0.72	1.23	1.73	1.88	11.00	3.30	19.0	1.73	1.31
13-cis-Retinal	472-86-6	1.27	1.24	0.23	0.82	1.04	1.24	1.63	1.72	2.61	1.59	6.09	1.48	1.21

Chemical_Name	CAS#	Mean[a]	Median[b]	SD	q01[c]	q05	q50	q95	q99	Fold Range q50-q01	Fold Range q50-q05	VIF[d]	Shrunken Fold Range q50-q01	Shrunken Fold Range q50-q05
Vitamin D3	67-97-0	1.26	1.25	0.17	0.80	1.01	1.25	1.85	1.96	2.80	1.71	1.57	2.28	1.54
N,N-Diethyl-p-phenylenediamine	93-05-0	1.23	1.25	0.29	0.80	0.98	1.25	1.66	1.86	2.78	1.85	2.14	2.01	1.52
Cadmium chloride	10108-64-2	1.27	1.26	0.23	0.47	0.93	1.25	1.69	1.91	5.96	2.10	2.29	3.25	1.63
2,4-Decadienal	25152-84-5	1.28	1.26	0.20	0.82	0.90	1.25	1.65	1.89	2.70	2.22	2.02	2.01	1.75
Bisphenol A diglycidyl ether	1675-54-3	1.34	1.26	0.25	0.20	0.74	1.25	1.73	1.88	11.23	3.21	-4.34	N/A	N/A
2,3,4,5-Tetrachlorophenol	4901-51-3	1.25	1.26	0.19	0.56	0.90	1.27	1.58	1.78	5.13	2.33	16.9	1.49	1.23
Bis(cyclopentadienyl)vanadium dichloride	12083-48-6	1.29	1.28	0.14	0.48	0.77	1.28	1.76	1.89	6.30	3.24	2.88	2.96	2.00
Ethyl linolenate	1191-41-9	1.31	1.29	0.16	0.91	1.05	1.28	1.66	1.84	2.32	1.68	-6.52	N/A	N/A
Chlordecone (kepone)	143-50-0	1.28	1.30	0.29	0.95	1.05	1.29	1.54	1.64	2.19	1.72	2.12	1.71	1.45
Retinol acetate	127-47-9	1.32	1.30	0.19	0.88	1.04	1.29	1.69	1.84	2.59	1.77	-29.3	N/A	N/A
4-(Chloroacetyl)acetanilide	140-49-8	1.31	1.32	0.30	-0.12	0.67	1.31	1.84	2.00	26.69	4.36	3.39	5.95	2.22
p-n-Nonylphenol	104-40-5	1.29	1.32	0.35	-0.02	0.58	1.31	1.87	2.05	21.33	5.36	5.35	3.76	2.07
p-Benzoquinone dioxime	105-11-3	1.24	1.32	0.50	0.19	0.77	1.33	1.78	1.93	14.01	3.63	-6.66	N/A	N/A
Glutaraldehyde	111-30-8	1.29	1.32	0.34	0.99	1.12	1.35	1.73	1.82	2.28	1.70	4.28	1.49	1.29
Chlorpheniramine maleate	113-92-8	1.32	1.36	0.37	-0.85	0.16	1.36	1.92	2.08	161.44	15.90	7.82	6.16	2.69
Oxymetholone	434-07-1	1.27	1.36	0.61	0.46	1.01	1.37	1.62	1.74	8.01	2.28	-5.22	N/A	N/A
Cadmium acetatedihydrate	4/4/5743	1.38	1.36	0.17	0.79	0.98	1.37	1.80	1.92	3.81	2.46	2.84	2.21	1.71
Flutamide	13311-84-7	1.29	1.37	0.55	-0.55	0.54	1.37	1.90	2.04	83.99	6.77	-2.33	N/A	N/A
2-Chloroacetophenone	532-27-4	1.36	1.37	0.20	0.88	1.09	1.41	1.78	1.91	3.40	2.08	3.11	2.00	1.51
1-Naphthylamine	134-32-7	1.39	1.38	0.25	-0.96	0.13	1.41	2.07	2.17	236.29	19.39	2.58	30.1	6.34
2-Pivalyl-1,3-indandione	83-26-1	1.32	1.41	0.59	0.53	0.96	1.42	1.86	2.05	7.81	2.90	-1.61	N/A	N/A
p-Nitrophenethyl alcohol	100-27-6	1.43	1.42	0.22	-0.81	-0.16	1.44	2.09	2.20	175.57	39.81	29.8	2.58	1.96
2,4-Hexadienal	142-83-6	1.43	1.43	0.26	0.96	1.14	1.45	1.86	1.95	3.13	2.05	3.55	1.83	1.46
8-Hydroxyquinoline	148-24-3	1.39	1.46	0.35	0.35	0.70	1.47	1.86	1.95	13.23	5.94	2.08	5.98	3.43
2-Biphenylamine	90-41-5	1.45	1.47	0.37	-0.86	-0.06	1.48	2.05	2.15	218.30	34.07	6.52	8.24	3.98
o-Phenylenediamine	95-54-5	1.48	1.47	0.21	0.33	0.78	1.48	1.97	2.13	14.16	4.99	3.13	4.48	2.48
3,4-Dinitrotoluene	610-39-9	1.42	1.48	0.43	0.41	0.80	1.49	1.99	2.16	12.03	4.88	4.59	3.19	2.10
3,4-Diaminotoluene	496-72-0	1.46	1.48	0.37	0.37	0.75	1.49	1.98	2.08	13.17	5.52	2.81	4.66	2.77
m-Nitrobenzyl chloride	619-23-8	1.47	1.49	0.34	-0.48	0.43	1.50	2.03	2.18	95.00	11.82	-6.41	N/A	N/A
2,3,5-Trichlorophenol	933-78-8	1.53	1.54	0.32	0.12	0.97	1.53	2.00	2.10	25.87	3.69	14.2	2.37	1.41
2',4',5'-Trihydroxybutyrophenone	1421-63-2	1.50	1.55	0.34	0.05	0.82	1.56	1.95	2.09	32.76	5.53	-2.54	N/A	N/A
Ninhydrin	485-47-2	1.55	1.57	0.23	0.92	1.16	1.58	1.88	1.95	4.54	2.65	1.99	2.92	1.99
cis-Dichlorodiamine platinum	15663-27-1	1.56	1.62	0.35	0.20	0.77	1.62	2.02	2.05	26.50	7.14	4.05	5.10	2.66
Propiconazole	60207-90-1	1.65	1.67	0.21	0.83	1.24	1.70	1.96	2.01	7.41	2.86	49.5	1.33	1.16
Rhein	478-43-3	1.64	1.69	0.32	0.50	1.07	1.71	2.02	2.22	16.02	4.34	2.14	6.66	2.73
Nifedipine	21829-25-4	1.63	1.70	0.35	0.92	1.18	1.72	1.96	2.09	6.29	3.43	4.67	2.34	1.77
Di(2-ethylhexyl) phthalate	117-81-7	1.68	1.72	0.22	0.62	1.20	1.72	2.04	2.22	12.57	3.31	2.25	5.40	2.22
o-Aminophenol	95-55-6	1.68	1.72	0.29	0.46	0.92	1.73	2.01	2.08	18.54	6.34	6.27	3.21	2.09
Verapamil HCl	152-11-4	1.64	1.72	0.35	0.55	1.08	1.74	1.96	2.17	15.35	4.57	26.5	1.70	1.34

Chemical_Name	CAS#	Mean[a]	Median[b]	SD	q01[c]	q05	q50	q95	q99	Fold Range q50-q01	Fold Range q50-q05	VIF[d]	Shrunken Fold Range q50-q01	Shrunken Fold Range q50-q05
Alizarin yellow R, free acid	2243-76-7	1.66	1.73	0.28	0.01	0.85	1.76	2.01	2.25	56.18	8.24	-14.3	N/A	N/A
Sulfathiazole	72-14-0	1.60	1.81	0.49	-0.63	0.36	1.81	2.24	2.28	274.74	28.12	3.55	19.72	5.88
Phenformin hydrochloride	834-28-6	1.77	1.82	0.22	1.03	1.33	1.82	2.06	2.15	6.26	3.13	2.03	3.63	2.23
Danthron	117-10-2	1.81	1.86	0.20	0.89	1.24	1.87	2.04	2.09	9.56	4.23	2.87	3.79	2.34
Dichlorvos (Vapona)	62-73-7	1.76	1.87	0.26	0.84	1.37	1.89	2.03	2.03	11.02	3.29	-2.84	N/A	N/A
Dimethyldipropylenetriamine	10563-29-8	1.81	1.90	0.24	0.80	1.25	1.94	2.04	2.04	13.73	4.91	22.2	1.74	1.40
Systhane	88671-89-0	1.87	1.94	0.22	0.63	1.42	1.94	2.08	2.08	20.54	3.30	3.10	5.56	1.97
1-(2,6,6-trimethylcyclohex-2-en-1-yl)pent-1-en-3-one	7779-30-8	1.83	1.94	0.26	1.10	1.63	1.95	2.04	2.11	7.19	2.09	3.49	2.88	1.49
Diisobutyl phthalate	84-69-5	1.91	1.95	0.14	1.08	1.59	1.96	2.06	2.06	7.48	2.33	-1.39	N/A	N/A
4-Methoxy-3-nitro-N-phenylbenzamide	97-32-5	1.94	1.96	0.17	1.33	1.75	1.97	2.04	2.04	4.37	1.68	-0.72	N/A	N/A
Azobenzene	103-33-3	1.96	1.97	0.12	0.60	1.14	1.97	2.05	2.05	23.67	6.79	-11.4	N/A	N/A
Ethoxyquin	91-53-2	1.87	1.97	0.28	0.45	1.39	1.98	2.08	2.08	33.89	3.84	4.35	5.42	1.91
Aldicarb	116-06-3	1.93	1.99	0.22	1.42	1.93	1.99	2.02	2.02	3.78	1.16	-4.24	N/A	N/A
Permethrin	52645-53-1	1.98	1.99	0.12	1.44	1.83	1.99	2.01	2.01	3.60	1.45	-3.71	N/A	N/A
4-Chloro-3,5-dinitro-a,a,a-trifluoride	393-75-9	1.97	1.99	0.09	0.60	1.94	2.00	2.01	2.01	25.02	1.15	6.55	3.52	1.06
3,4-Dichlorophenyl isocyanate	102-36-3	1.96	2.00	0.20	1.38	1.87	2.00	2.02	2.02	4.18	1.34	4.39	1.98	1.15
Styrene	100-42-5	1.98	2.00	0.11	1.78	2.00	2.00	2.00	2.00	1.64	1.01	2.15	1.40	1.00
5-(Hydroxymethyl)-2-furoic acid	6338-41-6	1.99	2.00	0.05	1.89	2.00	2.00	2.00	2.00	1.28	1.01	-0.54	N/A	N/A
trans-1,4-dichloro-2-butene	110-57-6	2.00	2.00	0.04	1.04	1.73	2.00	2.02	2.02	9.09	1.87	-9.26	N/A	N/A
Hexachloro-1,3-butadiene	87-68-3	1.97	2.00	0.14	1.79	2.00	2.00	2.00	2.00	1.64	1.00	4.34	1.27	1.00
Mono(2-ethylhexyl)phthalate	4376-20-9	1.99	2.00	0.08	1.48	1.98	2.00	2.00	2.00	3.32	1.05	-0.67	N/A	N/A
Methacrylonitrile	126-98-7	1.99	2.00	0.08	1.36	2.00	2.00	2.00	2.00	4.35	1.01	-7.21	N/A	N/A
1,2-Epoxy-3-chloropropane	106-89-8	1.98	2.00	0.15	1.35	1.99	2.00	2.01	2.01	4.47	1.02	-0.46	N/A	N/A
t-Butyl formate	762-75-4	1.98	2.00	0.09	0.48	1.50	2.00	2.02	2.02	33.57	3.19	-0.72	N/A	N/A

[a]Values shown are $\log_{10}$(molar concentration). [b]Table entries are sorted by the median value. [c]1st percentile. [d]See Methods for the shrinkage procedure, which estimates the fold-range after removing the effect of technical sampling variation.

Table S2. List of large-scale pharmacogenomics studies depicted in Figure 2a.

Author (Year)	Number of LCL Cell Lines Used	Number of Compounds Screened
Lock et al. 2012	81	240
Wheeler et al. 2013	608	2
Brown et al. 2014	520	29
Innocenti et al. 2009	372	4
Gamazon et al. 2010	343	5
Stark et al. 2010	270	11
Aksoy et al. 2009	240	3
O'Donnell et al. 2010	206	2
Li et al. 2008	197	2
Li et al. 2010	194	3
Huang et al. 2007	176	4
Fridley et al. 2011	175	2
Li et al. 2009	174	2
Peters et al. 2011	124	29
Huang et al. 2011	107	3
Wheeler et al. 2011	83	4
O'Shea et al. 2011	81	14
Kulkarni et al. 2012	55	2
Brown et al. 2012	516	1

References for Supplemental Material, Table S2.

Aksoy P, Zhu MJ, Kalari KR, Moon I, Pelleymounter LL, Eckloff BW, et al. 2009. Cytosolic 5'-nucleotidase iii (nt5c3): Gene sequence variation and functional genomics. Pharmacogenet Genomics 19:567-576.

Brown CC, Havener TM, Medina MW, Auman JT, Mangravite LM, Krauss RM, et al. 2012. A genome-wide association analysis of temozolomide response using lymphoblastoid cell lines shows a clinically relevant association with mgmt. Pharmacogenet Genomics 22:796-802.

Brown CC, Havener TM, Medina MW, Jack JR, Krauss RM, McLeod HL, et al. 2014. Genome-wide association and pharmacological profiling of 29 anticancer agents using lymphoblastoid cell lines. Pharmacogenomics 15:137-146.

Fridley BL, Batzler A, Li L, Li F, Matimba A, Jenkins GD, et al. 2011. Gene set analysis of purine and pyrimidine antimetabolites cancer therapies. Pharmacogenet Genomics 21:701-712.

Gamazon ER, Huang RS, Cox NJ, Dolan ME. 2010. Chemotherapeutic drug susceptibility associated snps are enriched in expression quantitative trait loci. Proc Natl Acad Sci USA 107:9287-9292.

Huang RS, Kistner EO, Bleibel WK, Shukla SJ, Dolan ME. 2007. Effect of population and gender on chemotherapeutic agent-induced cytotoxicity. Mol Cancer Ther 6:31-36.

Huang RS, Gamazon ER, Ziliak D, Wen Y, Im HK, Zhang W, et al. 2011. Population differences in microrna expression and biological implications. RNA Biol 8:692-701.

Innocenti F, Mirkov S, Nagasubramanian R, Ramirez J, Liu W, Bleibel WK, et al. 2009. The werner's syndrome 4330t>c (cys1367arg) gene variant does not affect the in vitro cytotoxicity of topoisomerase inhibitors and platinum compounds. Cancer Chemother Pharmacol 63:881-887.

Kulkarni H, Goring HH, Diego V, Cole S, Walder KR, Collier GR, et al. 2012. Association of differential gene expression with imatinib mesylate and omacetaxine mepesuccinate toxicity in lymphoblastoid cell lines. BMC Med Genomics 5:37.

Li F, Fridley BL, Matimba A, Kalari KR, Pelleymounter L, Moon I, et al. 2010. Ecto-5'-nucleotidase and thiopurine cellular circulation: Association with cytotoxicity. Drug Metab Dispos 38:2329-2338.

Li L, Fridley B, Kalari K, Jenkins G, Batzler A, Safgren S, et al. 2008. Gemcitabine and cytosine arabinoside cytotoxicity: Association with lymphoblastoid cell expression. Cancer Res 68:7050-7058.

Li L, Fridley BL, Kalari K, Jenkins G, Batzler A, Weinshilboum RM, et al. 2009. Gemcitabine and arabinosylcytosin pharmacogenomics: Genome-wide association and drug response biomarkers. PLoS One 4:e7765.

Lock EF, Abdo N, Huang R, Xia M, Kosyk O, O'Shea SH, et al. 2012. Quantitative high-throughput screening for chemical toxicity in a population-based in vitro model. Toxicol Sci 126:578-588.

O'Donnell PH, Gamazon E, Zhang W, Stark AL, Kistner-Griffin EO, Stephanie Huang R, et al. 2010. Population differences in platinum toxicity as a means to identify novel genetic susceptibility variants. Pharmacogenet Genomics 20:327-337.

O'Shea SH, Schwarz J, Kosyk O, Ross PK, Ha MJ, Wright FA, et al. 2011. In vitro screening for population variability in chemical toxicity. Toxicol Sci 119:398-407.

Peters EJ, Motsinger-Reif A, Havener TM, Everitt L, Hardison NE, Watson VG, et al. 2011. Pharmacogenomic characterization of us fda-approved cytotoxic drugs. Pharmacogenomics 12:1407-1415.

Stark AL, Zhang W, Mi S, Duan S, O'Donnell PH, Huang RS, et al. 2010. Heritable and non-genetic factors as variables of pharmacologic phenotypes in lymphoblastoid cell lines. Pharmacogenomics J 10:505-512.

Wheeler HE, Gorsic LK, Welsh M, Stark AL, Gamazon ER, Cox NJ, et al. 2011. Genome-wide local ancestry approach identifies genes and variants associated with chemotherapeutic susceptibility in african americans. PLoS One 6:e21920.

Wheeler HE, Gamazon ER, Stark AL, O'Donnell PH, Gorsic LK, Huang RS, et al. 2013. Genome-wide meta-analysis identifies variants associated with platinating agent susceptibility across populations. Pharmacogenomics J 13:35-43.

Table S3. Chemicals showing significant EC_{10} variation across populations or by sex.

Chemical_Name	CAS#	*P* population differences	*q* population differences	*P* sex differences	*q* sex differences
Azathioprine	446-86-6	3.8E-13	6.8E-11	0.228	0.506
5-Fluorouracil	51-21-8	9.3E-13	8.3E-11	0.003	0.078
1,3,5-Triglycidyl isocyanurate	2451-62-9	7.4E-10	4.4E-08	0.223	0.504
Diglycidyl resorcinol ether	101-90-6	1.6E-09	7.0E-08	0.042	0.217
6-Thioguanine	154-42-7	2.4E-09	8.4E-08	0.804	0.882
6-Mercaptopurine monohydrate	6112-76-1	8.1E-09	2.4E-07	0.898	0.918
Turmeric	458-37-7	2.1E-08	5.5E-07	0.031	0.196
Phenformin hydrochloride	834-28-6	8.1E-08	1.8E-06	0.289	0.556
Tetrachlorvinphos	961-11-5	3.4E-06	6.7E-05	0.517	0.735
Cycloheximide	66-81-9	6.0E-06	1.1E-04	0.778	0.882
N,N-Dimethyl-p-nitrosoaniline	138-89-6	8.9E-06	1.4E-04	0.018	0.162
Hematoxylin	517-28-2	1.1E-05	1.6E-04	0.643	0.822
Diethylene glycol diacrylate	4074-88-8	1.2E-05	1.6E-04	0.012	0.131
Cadmium acetate dihydrate	4--4--5743	1.4E-05	1.8E-04	0.757	0.882
Sodium dichromate dihydrate (VI)	7789-12-0	1.6E-05	1.9E-04	0.050	0.218
beta-Nitrostyrene	5153-67-3	1.8E-05	2.0E-04	0.229	0.506
N-Methyl-p-aminophenol sulfate	55-55-0	2.1E-05	2.2E-04	0.051	0.219
Tetraethylene glycol diacrylate	17831-71-9	3.8E-05	3.8E-04	0.023	0.180
Tetraethylene glycol diacrylate	17831-71-9	4.6E-05	4.4E-04	0.050	0.218
1,1,1,2-Tetrabromoethane	630-16-0	5.8E-05	0.001	0.368	0.609
Pyrimethamine	58-14-0	7.1E-05	0.001	0.650	0.826
2,2',4'-Trichloroacetophenone	4252-78-2	6.9E-05	0.001	0.746	0.879
Malachite green oxalate	2437-29-8	6.7E-05	0.001	0.567	0.774
Retinal	116-31-4	7.6E-05	0.001	0.880	0.906
t-Butylhydroquinone	1948-33-0	1.2E-04	0.001	0.070	0.249
Methyl mercuric (II) chloride	115-09-3	1.2E-04	0.001	0.067	0.247
Dieldrin	60-57-1	1.2E-04	0.001	0.007	0.121
Sodium dichromate dihydrate (VI)	7789-12-0	1.1E-04	0.001	0.027	0.196
2-Amino-4-chlorophenol	95-85-2	1.7E-04	0.001	0.808	0.882
Nitrogen mustard hydrochloride	55-86-7	2.0E-04	0.001	0.077	0.251
Verapamil HCl	152-11-4	2.4E-04	0.001	0.425	0.661
Triamterene	396-01-0	3.6E-04	0.002	0.486	0.722
N-Isopropyl-N'-phenyl-p-phenylenediamine	101-72-4	3.6E-04	0.002	0.359	0.601
Cycloheximide	66-81-9	3.8E-04	0.002	0.880	0.906
Alizarin Yellow R, free acid	2243-76-7	4.0E-04	0.002	0.061	0.239
Benzethonium chloride	121-54-0	0.001	0.003	0.076	0.251
9-Aminoacridine, monohydrochloride, monohydrate	52417-22-8	0.001	0.003	0.604	0.795
Dexamethazone	50-02-2	0.001	0.003	0.571	0.774
3,4-Dinitrotoluene	610-39-9	0.001	0.003	0.008	0.121
Retinol acetate	127-47-9	0.001	0.004	0.094	0.295
p-Nitrosodiphenylamine	156-10-5	0.001	0.004	0.022	0.180
Glutaraldehyde	111-30-8	0.001	0.004	0.131	0.392
trans-1,4-dichloro-2-butene	110-57-6	0.001	0.004	0.177	0.441
Hexachlorophene	70-30-4	0.001	0.005	0.856	0.902
Acetochlor	34256-82-1	0.001	0.005	0.257	0.548
Titanocene dichloride	1271-19-8	0.001	0.005	0.849	0.902
Chlordecone (kepone)	143-50-0	0.001	0.005	0.046	0.218
Nifedipine	21829-25-4	0.002	0.006	0.000	0.022
Saquinavir mesylate	149845-06-7	0.002	0.006	0.141	0.395
Chlordecone (kepone)	143-50-0	0.002	0.006	0.010	0.128
Dimethyldipropylenetriamine	10563-29-8	0.002	0.006	0.308	0.562

Chemical_Name	CAS#	*P* population differences	*q* population differences	*P* sex differences	*q* sex differences
Zinc pyrithione	13463-41-7	0.002	0.007	0.275	0.556
1-Methyl-3-nitro-1-nitroso-guanidine	70-25-7	0.003	0.009	0.015	0.150
Cetylpyridinium bromide	140-72-7	0.003	0.011	0.010	0.128
8-Hydroxyquinoline	148-24-3	0.004	0.012	0.193	0.474
Potassium dichromate	7778-50-9	0.004	0.013	0.067	0.247
2,4-Difluoronitrobenzene	446-35-5	0.004	0.013	0.163	0.418
p-tert-Butylcatechol	98-29-3	0.004	0.013	0.618	0.802
t-Butylhydroquinone	1948-33-0	0.004	0.013	0.702	0.861
p-n-Nonylphenol	104-40-5	0.005	0.015	0.143	0.395
Amiloride hydrochloride	2016-88-8	0.006	0.016	0.011	0.128
17beta-Estradiol	50-28-2	0.006	0.016	0.154	0.399
Ethyl linolenate	1191-41-9	0.006	0.016	0.045	0.218
Pentaerythritol triacrylate	3524-68-3	0.008	0.023	0.001	0.050
Di(2-ethylhexyl) phthalate	117-81-7	0.008	0.023	0.216	0.499
Bis(cyclopentadienyl)vanadium chloride	12083-48-6	0.008	0.023	0.720	0.871
Dichlorvos (Vapona)	62-73-7	0.009	0.025	0.282	0.556
1,6-Hexamethylene diacrylate	13048-33-4	0.010	0.025	0.002	0.073
N,N-Diethyl-p-phenylenediamine	93-05-0	0.010	0.027	0.303	0.562
8-Hydroxyquinoline	148-24-3	0.011	0.027	0.280	0.556
Ethidium bromide	1239-45-8	0.011	0.027	0.787	0.882
4-Methoxy-3-nitro-N-phenylbenzamide	97-32-5	0.013	0.033	0.558	0.768
Dibromoacetonitrile	3252-43-5	0.015	0.038	0.248	0.535
4-Amino-4'-hydroxy-3-methyl-diphenylamine	6219-89-2	0.017	0.042	0.786	0.882
Hexamethyl-p-rosaniline chloride	548-62-9	0.018	0.043	0.864	0.902
4,4-Thiobis(6-tert-butyl-m-cresol)	96-69-5	0.019	0.044	0.345	0.600
2-Chloroacetophenone (CN)	532-27-4	0.020	0.046	0.071	0.249
2-Amino-4-methylphenol	95-84-1	0.021	0.047	0.594	0.794
Ninhydrin	485-47-2	0.022	0.050	0.389	0.627
o-Phenanthroline	66-71-7	0.022	0.050	0.075	0.251
Phenylmercuric acetate	62-38-4	0.169	0.237	0.000	0.037

P-values were obtained by running analyses of variance on $\log_{10}(EC_{10})$ with subpopulation or sex as a categorical variable. *q*-values were obtained after Benjamini-Hochberg false discovery rate correction per chemical.

Table S4. Trio-based heritability (h^2) estimates.

Chemical_Name	CAS#	h^{2a}	h^2 standard error[a]	P^b	q-value[c]
Diglycidyl resorcinol ether	101-90-6	48.49	11.03	4.62E-05	6.47E-03
1,8-Dihydroxy-4,5-dinitroanthraquinone	81-55-0	42.02	11.07	2.41E-04	1.68E-02
Reserpine	50-55-5	38.40	12.24	1.82E-03	7.04E-02
o-Phenylenediamine	95-54-5	36.51	13.05	4.65E-03	9.25E-02
5-Fluorouracil	51-21-8	36.01	12.03	2.51E-03	7.04E-02
17beta-Estradiol	50-28-2	35.45	12.39	3.65E-03	8.52E-02
Sulfathiazole	72-14-0	35.24	11.80	2.47E-03	7.04E-02
1,3,5-Triglycidyl isocyanurate	2451-62-9	31.51	11.76	5.28E-03	9.25E-02
9-Aminoacridine, monohydrochloride, monohydrate	52417-22-8	30.35	12.74	1.15E-02	1.50E-01
p-Aminophenol	123-30-8	29.93	13.04	1.43E-02	1.64E-01
Tetrachlorvinphos	961-11-5	28.51	12.09	1.17E-02	1.50E-01
Dichloroacetonitrile	3018-12-0	27.66	12.48	1.64E-02	1.64E-01
Diethylene glycol diacrylate	4074-88-8	26.97	11.52	1.18E-02	1.50E-01
m-Nitrobenzyl chloride	619-23-8	26.75	12.74	2.13E-02	1.82E-01
Chlorambucil	305-03-3	26.21	11.76	1.54E-02	1.64E-01
1-Methyl-3-nitro-1-nitroso-guanidine	70-25-7	25.71	12.38	2.22E-02	1.82E-01
N-(1,3-Dimethylbutyl)-N'-phenyl-p-phenylenediamine	793-24-8	25.19	20.99	1.30E-01	3.91E-01
Cycloheximide	66-81-9	24.75	11.94	2.20E-02	1.82E-01
Progesterone	57-83-0	24.02	16.95	8.69E-02	3.91E-01
cis-Dichlorodiamine platinum	15663-27-1	23.32	12.87	3.89E-02	2.61E-01
2,3,4,5-Tetrachloronitrobenzene	879-39-0	22.98	12.88	4.11E-02	2.62E-01
Diisobutyl phthalate	84-69-5	22.83	12.52	3.77E-02	2.61E-01
Tetraethylene glycol diacrylate	17831-71-9	22.81	11.59	2.73E-02	2.12E-01
Domiphen bromide	538-71-6	21.96	12.19	3.92E-02	3.69E-01
Chlordecone (kepone)	143-50-0	21.88	14.70	7.39E-02	2.61E-01
Colchicine	64-86-8	20.96	13.11	5.89E-02	3.31E-01
Dimethyldipropylenetriamine	10563-29-8	20.72	12.45	5.14E-02	3.13E-01
Catechol	120-80-9	20.07	12.89	6.35E-02	3.31E-01
Ninhydrin	485-47-2	19.73	12.55	6.14E-02	3.31E-01
o-Aminophenol	95-55-6	19.41	12.51	6.38E-02	3.31E-01
Hexachlorophene	70-30-4	18.48	13.22	8.47E-02	3.91E-01
Retinal	116-31-4	17.32	12.03	7.78E-02	3.76E-01
Dieldrin	60-57-1	16.80	20.69	2.14E-01	3.91E-01
Guggulsterones E	39025-24-6	16.53	13.94	1.21E-01	3.91E-01
Pyrimethamine	58-14-0	16.16	12.46	1.00E-01	3.91E-01
2,3,4,5-Tetrachlorophenol	4901-51-3	16.14	12.68	1.04E-01	3.91E-01
1,3-Diiminobenz (f)-isoindoline	65558-69-2	15.67	12.67	1.11E-01	3.91E-01
3-Chloro-4-(dichloromethyl)-5-hydroxy-2(5H)-furanone(MX)	77439-76-0	15.58	13.28	1.23E-01	3.91E-01
2-Amino-4-chlorophenol	95-85-2	15.20	11.79	1.01E-01	3.91E-01
Turmeric	458-37-7	15.01	11.68	1.02E-01	3.91E-01
N,N'-Diphenyl-p-phenylenediamine	74-31-7	14.07	13.33	1.48E-01	3.91E-01
Ethyl linolenate	1191-41-9	13.68	11.05	1.10E-01	3.91E-01
6-Thioguanine	154-42-7	13.43	12.79	1.49E-01	3.91E-01
1,2-Epoxy-3-chloropropane	106-89-8	12.97	14.63	1.90E-01	3.91E-01
2,2',4'-Trichloroacetophenone	4252-78-2	12.69	12.22	1.51E-01	3.91E-01
Malachite green oxalate	2437-29-8	12.61	12.82	1.65E-01	3.91E-01
Aflatoxin B1 from Aspergillus flavus	1162-65-8	12.51	13.52	1.79E-01	3.91E-01
Amiloride hydrochloride	2016-88-8	12.45	13.10	1.73E-01	3.91E-01
Captan	133-06-2	12.43	13.33	1.77E-01	3.91E-01
Cetylpyridinium bromide	140-72-7	12.34	11.69	1.47E-01	3.91E-01
p-Benzoquinone dioxime	105-11-3	11.80	12.96	1.83E-01	3.91E-01
Nifedipine	21829-25-4	11.78	13.14	1.87E-01	3.91E-01

**Chemical_Name**	**CAS#**	***h*[2a]**	***h*² standard error[a]**	***P*[b]**	***q-value*[c]**
t-Butylhydroquinone	1948-33-0	11.66	12.33	1.74E-01	3.91E-01
Toxaphene	8001-35-2	11.01	12.60	1.92E-01	3.91E-01
Bisphenol A diglycidyl ether	1675-54-3	10.66	12.40	1.96E-01	3.91E-01
N-Isopropyl-N'-phenyl-p-phenylenediamine	101-72-4	10.55	12.58	2.02E-01	3.91E-01
3,4-Diaminotoluene	496-72-0	10.34	13.90	2.30E-01	3.91E-01
o-Nitrobenzyl chloride	612-23-7	10.05	12.31	2.08E-01	3.91E-01
Rhein	478-43-3	9.86	13.04	2.26E-01	3.91E-01
p-tert-Butylcatechol	98-29-3	9.71	12.73	2.24E-01	3.91E-01
2-Amino-4-methylphenol	95-84-1	8.64	12.64	2.48E-01	3.91E-01
N,N-Diethyl-p-phenylenediamine	93-05-0	8.63	11.96	2.36E-01	3.91E-01
Cadmium acetatedihydrate	4/4/5743	8.55	11.50	2.29E-01	3.91E-01
Chlorhexidine	55-56-1	8.04	13.48	2.76E-01	3.91E-01
4-(Chloroacetyl)acetanilide	140-49-8	7.90	12.82	2.69E-01	3.91E-01
Glutaraldehyde	111-30-8	7.83	12.80	2.71E-01	3.91E-01
Tamoxifen citrate	54965-24-1	7.48	12.08	2.69E-01	3.91E-01
Fumaronitrile	764-42-1	7.35	11.68	2.65E-01	3.91E-01
Titanocene dichloride	1271-19-8	7.31	11.62	2.65E-01	3.91E-01
Benzethonium chloride	121-54-0	7.17	11.87	2.73E-01	3.91E-01
Saquinavir mesylate	149845-06-7	7.17	15.00	3.17E-01	3.91E-01
3,4-Dinitrotoluene	610-39-9	7.16	12.48	2.83E-01	3.91E-01
4,4-Thiobis(6-tert-butyl-m-cresol)	96-69-5	7.14	12.30	2.81E-01	3.91E-01
Captan	133-06-2	7.11	15.37	3.22E-01	3.91E-01
Oxymetholone	434-07-1	6.99	12.12	2.83E-01	3.91E-01
2-Biphenylamine	90-41-5	6.83	12.64	2.95E-01	3.91E-01
Hexamethyl-p-rosaniline chloride	548-62-9	6.46	12.23	2.99E-01	3.91E-01
p-Quinone	106-51-4	6.28	12.40	3.07E-01	3.91E-01
Chlordecone (kepone)	143-50-0	6.03	13.55	3.29E-01	3.91E-01
Di(2-ethylhexyl) phthalate	117-81-7	5.72	15.48	3.56E-01	3.91E-01
Cycloheximide	66-81-9	5.21	11.49	3.25E-01	3.91E-01
Dichlorvos (Vapona)	62-73-7	4.51	11.06	3.42E-01	3.91E-01
Sodium dichromate dihydrate (VI)	7789-12-0	4.37	11.72	3.55E-01	3.91E-01
Ethoxyquin	91-53-2	3.82	11.61	3.71E-01	3.91E-01
Ethacrynic acid	58-54-8	3.59	12.04	3.83E-01	3.91E-01
Phenylmercuric acetate	62-38-4	3.56	12.32	3.86E-01	3.91E-01
Azathioprine	446-86-6	3.43	12.21	3.89E-01	3.91E-01
Ergotamine tartrate	379-79-3	3.35	13.77	4.04E-01	3.91E-01
Daunomycin HCL	23541-50-6	2.25	11.96	4.25E-01	3.91E-01
6-Mercaptopurine monohydrate	6112-76-1	2.20	13.95	4.37E-01	3.91E-01
1,3-Dicyclohexylcarbodiimide	538-75-0	2.15	12.60	4.32E-01	3.91E-01
2-Pivalyl-1,3-indandione	83-26-1	2.02	13.89	4.42E-01	3.91E-01
Dexamethazone	50-02-2	1.56	13.56	4.54E-01	3.91E-01
Melatonin	73-31-4	1.36	13.70	4.61E-01	3.91E-01
4-Chloro-3,5-dinitro-a,a,a-trifluorotoluene	393-75-9	1.36	17.13	4.68E-01	3.91E-01
Aldrin	309-00-2	1.21	13.73	4.65E-01	3.91E-01
Ethidium bromide	1239-45-8	1.19	12.24	4.61E-01	3.91E-01
Systhane	88671-89-0	1.12	14.90	4.70E-01	3.91E-01
2,2'-Thiobis(4,6-dichlorophenol)	97-18-7	1.00	13.04	4.69E-01	3.91E-01
Propiconazole	60207-90-1	0.93	12.81	4.71E-01	3.91E-01
1,6-Hexamethylene diacrylate	13048-33-4	0.90	10.20	4.65E-01	3.91E-01
p-Nitrosodiphenylamine	156-10-5	0.76	11.80	4.74E-01	3.91E-01
Acetochlor	34256-82-1	0.72	11.99	4.76E-01	3.91E-01
Nitazoxanide	55981-09-4	0.68	12.84	4.79E-01	3.91E-01
1,1,1,2-Tetrabromoethane	630-16-0	0.67	13.23	4.80E-01	3.91E-01
Sodium dichromate dihydrate (VI)	7789-12-0	0.63	12.00	4.79E-01	3.91E-01

Chemical_Name	CAS#	h^{2a}	h^2 standard error[a]	P^b	q-value[c]
N,N'-Di-sec-butyl-p-phenyldiamine	101-96-2	0.61	12.81	4.81E-01	3.91E-01
t-Butylhydroquinone	1948-33-0	0.61	13.47	4.82E-01	3.91E-01
2,4-Hexadienal	142-83-6	0.58	11.35	4.80E-01	3.91E-01
Iodochlorohydroxyquinoline	130-26-7	0.00	NA	5.00E-01	3.91E-01
Cadmium chloride	10108-64-2	0.00	NA	5.00E-01	3.91E-01
5-(Hydroxymethyl)-2-furoic acid	6338-41-6	0.00	NA	5.00E-01	3.91E-01
o-Phenanthroline	66-71-7	0.00	NA	5.00E-01	3.91E-01
Amitriptyline HCl	549-18-8	0.00	NA	5.00E-01	3.91E-01
Flutamide	13311-84-7	0.00	NA	5.00E-01	3.91E-01
Hydroquinone	123-31-9	0.00	NA	5.00E-01	3.91E-01
Progesterone	57-83-0	0.00	NA	5.00E-01	3.91E-01
Triamterene	396-01-0	0.00	NA	5.00E-01	3.91E-01
Verapamil HCl	152-11-4	0.00	NA	5.00E-01	3.91E-01
Phenformin hydrochloride	834-28-6	0.00	NA	5.00E-01	3.91E-01
Dazomet	533-74-4	0.00	NA	5.00E-01	3.91E-01
2,4-Decadienal	25152-84-5	0.00	NA	5.00E-01	3.91E-01
8-Hydroxyquinoline	148-24-3	0.00	NA	5.00E-01	3.91E-01
8-Hydroxyquinoline	148-24-3	0.00	NA	5.00E-01	3.91E-01
Methacrylonitrile	126-98-7	0.00	NA	5.00E-01	3.91E-01
Potassium dichromate	7778-50-9	0.00	NA	5.00E-01	3.91E-01
Retinol acetate	127-47-9	0.00	NA	5.00E-01	3.91E-01
Mono(2-ethylhexyl)phthalate	4376-20-9	0.00	NA	5.00E-01	3.91E-01
Aldicarb	116-06-3	0.00	NA	5.00E-01	3.91E-01
13-cis-Retinal	472-86-6	0.00	NA	5.00E-01	3.91E-01
Alizarin Yellow R, free acid	2243-76-7	0.00	NA	5.00E-01	3.91E-01
Methyl mercuric (II) chloride	115-09-3	0.00	NA	5.00E-01	3.91E-01
p-n-Nonylphenol	104-40-5	0.00	NA	5.00E-01	3.91E-01
3,4-Dichlorophenyl isocyanate	102-36-3	0.00	NA	5.00E-01	3.91E-01
2,4-Difluoronitrobenzene	446-35-5	0.00	NA	5.00E-01	3.91E-01
tetra-N-Octylammonium bromide	14866-33-2	0.00	NA	5.00E-01	3.91E-01
Tetraethylene glycol diacrylate	17831-71-9	0.00	NA	5.00E-01	3.91E-01
Styrene	100-42-5	0.00	NA	5.00E-01	3.91E-01
trans-1,4-dichloro-2-butene	110-57-6	0.00	NA	5.00E-01	3.91E-01
1-Naphthylamine	134-32-7	0.00	NA	5.00E-01	3.91E-01
beta-Nitrostyrene	5153-67-3	0.00	NA	5.00E-01	3.91E-01
N,N,N',N'-Tetramethyl-p-phenylenediamine	100-22-1	0.00	NA	5.00E-01	3.91E-01
2-Chloroacetophenone	532-27-4	0.00	NA	5.00E-01	3.91E-01
HC blue 2	33229-34-4	0.00	NA	5.00E-01	3.91E-01
Chloranil	118-75-2	0.00	NA	5.00E-01	3.91E-01
Hexachloro-1,3-butadiene	87-68-3	0.00	NA	5.00E-01	3.91E-01
Danthron	117-10-2	0.00	NA	5.00E-01	3.91E-01
N-Methyl-p-aminophenol sulfate	55-55-0	0.00	NA	5.00E-01	3.91E-01
Dibromoacetonitrile	3252-43-5	0.00	NA	5.00E-01	3.91E-01
Pentaerythritol triacrylate	3524-68-3	0.00	NA	5.00E-01	3.91E-01
Chlordane (technical grade)	12789-03-6	0.00	NA	5.00E-01	3.91E-01
Dibromonitromethane	598-91-4	0.00	NA	5.00E-01	3.91E-01
Endosulfan	115-29-7	0.00	NA	5.00E-01	3.91E-01
7,12-Dimethylbenzanthracene	57-97-6	0.00	NA	5.00E-01	3.91E-01
Methylene bis(thiocyanate)	6317-18-6	0.00	NA	5.00E-01	3.91E-01
Tetramethylthiouram disulfide	137-26-8	0.00	NA	5.00E-01	3.91E-01
Mercuric chloride	7487-94-7	0.00	NA	5.00E-01	3.91E-01
p-Nitrophenethyl alcohol	100-27-6	0.00	NA	5.00E-01	3.91E-01
4-Chloro-o-phenylenediamine	95-83-0	0.00	NA	5.00E-01	3.91E-01
t-Butyl formate	762-75-4	0.00	NA	5.00E-01	3.91E-01

Chemical_Name	CAS#	h^{2a}	h^2 standard error[a]	P^b	q-value[c]
N,N-Dimethyl-p-nitrosoaniline	138-89-6	0.00	NA	5.00E-01	3.91E-01
Bis(cyclopentadienyl)vanadium chloride	12083-48-6	0.00	NA	5.00E-01	3.91E-01
2,3,5-Trichlorophenol	933-78-8	0.00	NA	5.00E-01	3.91E-01
4-Amino-4'-hydroxy-3-methyl-diphenylamine	6219-89-2	0.00	NA	5.00E-01	3.91E-01
Nitrogen mustard hydrochloride	55-86-7	0.00	NA	5.00E-01	3.91E-01
4-Methoxy-3-nitro-N-phenylbenzamide	97-32-5	0.00	NA	5.00E-01	3.91E-01
2',4',5'-Trihydroxybutyrophenone	1421-63-2	0.00	NA	5.00E-01	3.91E-01
1-(2,6,6-Trimethyl-2-cyclohexene-1-yl)-1-penten-3-one	7779-30-8	0.00	NA	5.00E-01	3.91E-01
Azobenzene	103-33-3	0.00	NA	5.00E-01	3.91E-01
2-Octyl-3-isothiazolone	26530-20-1	0.00	NA	5.00E-01	3.91E-01
N-(1-Naphthyl)ethylenediamine dihydrochloride	1465-25-4	0.00	NA	5.00E-01	3.91E-01
Chlorpheniramine maleate	113-92-8	0.00	NA	5.00E-01	3.91E-01
Colchicine	64-86-8	0.00	NA	5.00E-01	3.91E-01
Hematoxylin	517-28-2	0.00	NA	5.00E-01	3.91E-01
Ziram	137-30-4	0.00	NA	5.00E-01	3.91E-01
permethrin	52645-53-1	0.00	NA	5.00E-01	3.91E-01
Digoxin	20830-75-5	0.00	NA	5.00E-01	3.91E-01
Vitamin D3	67-97-0	0.00	NA	5.00E-01	3.91E-01
Zinc pyrithione	13463-41-7	0.00	NA	5.00E-01	3.91E-01

[a]In percent, obtained from MERLIN package as described in Methods, table entries sorted by decreasing h^2. [b]Obtained from SOLAR package as described in Methods. [c]False-discovery *q*-values obtained using the *qvalue* function in *R*3.02.

Table S5. Significant EC_{10} –SNP associations among set of 1.3m SNPs.

**Chemical_Name**	**CAS #**	**SNP**	**bp[a]**	**Chrom**	**Gene**	***P*[b]**	***q*-value[c]**
Chlordecone (kepone)	143-50-0	rs28502033	71440392	7	*CALN1*	1.29E-10	2E-04
Tetramethylthiouram disulfide	137-26-8	rs74487456	87708146	15	*AGBL1*	3.13E-10	4E-04
2,2'-Thiobis(4,6-dichlorophenol)	97-18-7	rs78022668	220093749	1	*SLC30A10*	3.28E-10	4E-04
3-Chloro-4-(dichloromethyl)-5-hydroxy-2(5H)-furanone(MX)	77439-76-0	rs114147671	80699899	16	*CDYL2*	1.23E-09	0.002
2,4-Hexadienal	142-83-6	rs499384	33582090	6	*BAK1*	1.93E-09	0.003
1,3-Diiminobenz (f)-isoindoline	65558-69-2	rs11813930	21429432	10	*C10orf113*	2.13E-09	0.003
2,3,4,5-Tetrachlorophenol	4901-51-3	rs61732507	38128932	21	*HLCS*	2.35E-09	0.003
N-Methyl-p-aminophenol sulfate	55-55-0	rs13120371	139092719	4	*SLC7A11*	3.97E-09	0.005
4-Amino-4’-hydroxy-3-methyl-diphenylamineenylamine	6219-89-2	rs75523194	86316969	7	*GRM3*	6.78E-09	0.009
1,8-Dihydroxy-4,5-dinitroanthraquinoneuinone	81-55-0	rs75198473	24164198	10	*KIAA1217*	7.92E-09	0.011
cis-Dichlorodiamine platinum	15663-27-1	rs6461533	21082986	7	*SP8*	8.25E-09	0.011
Dichloroacetonitrile	3018-12-0	rs75207133	12319584	9	*TYRP1*	8.74E-09	0.012
Amiloride hydrochloride	2016-88-8	rs74631305	66772843	12	*GRIP1*	9.18E-09	0.012
Domiphen bromide	538-71-6	rs78468118	82700651	16	*CDH13*	9.24E-09	0.009
o-Phenylenediamine	95-54-5	rs28473317	100407305	15	*ADAMTS17*	9.34E-09	0.009
t-Butyl formate	762-75-4	rs17044941	23433191	2	*KLHL29*	9.43E-09	0.013
N,N,N’,N’-Tetramethyl-p-phenylenediamineediamine	100-22-1	rs9825285	171722475	3	*TMEM212*	9.50E-09	0.013
Cycloheximide	66-81-9	rs75192401	107761225	11	*RAB39*	1.02E-08	0.014
Progesterone	57-83-0	rs60732724	153798056	5	*GALNT10*	1.14E-08	0.015
Domiphen bromide	538-71-6	rs75611658	103934813	13	*SLC10A2*	1.29E-08	0.009
o-Phenylenediamine	95-54-5	rs113019120	29196546	13	*SLC46A3*	1.39E-08	0.009
1,2-Epoxy-3-chloropropane	106-89-8	rs74422342	128543398	7	*KCP*	1.53E-08	0.015
Alizarin Yellow R, free acid	2243-76-7	rs78618741	168250523	2	*XIRP2*	1.59E-08	0.021
17beta-Estradiol	50-28-2	rs76341199	73932870	6	*KHDC1L*	1.90E-08	0.025
Aflatoxin B1 from Aspergillus flavus	1162-65-8	rs73277691	35495326	20	*C20orf118*	1.96E-08	0.017
1,1,1,2-Tetrabromoethane	630-16-0	rs73737933	8015575	5	*MTRR*	2.07E-08	0.015
2',4',5'-Trihydroxybutyrophenone	1421-63-2	rs10213832	172302409	5	*ERGIC1*	2.10E-08	0.028
Diethylene glycol diacrylate	4074-88-8	rs75191956	51639820	15	*GLDN*	2.11E-08	0.028
8-Hydroxyquinoline	148-24-3	rs9477330	17135067	6	*RBM24*	2.20E-08	0.021
1,2-Epoxy-3-chloropropane	106-89-8	rs73109015	63790727	5	*RGS7BP*	2.28E-08	0.015
Mercuric chloride	7487-94-7	rs5963392	38144323	X	*RPGR*	2.30E-08	0.031
1,3-Diiminobenz (f)-isoindoline	65558-69-2	rs114448235	101178035	12	*ANO4*	2.43E-08	0.016
Melatonin	73-31-4	rs6473423	84134051	8	*SNX16*	2.55E-08	0.034
2-Octyl-3-isothiazolone	26530-20-1	rs57831318	154355658	3	*GPR149*	2.58E-08	0.020
Chlordane (technical grade)	12789-03-6	rs4531541	75668192	12	*CAPS2*	2.67E-08	0.035
N-(1,3-Dimethylbutyl)-N’-phenyl-p-phenylenediamine	793-24-8	rs17173040	144146779	7	*NOBOX*	2.67E-08	0.031
2-Chloroacetophenone	532-27-4	rs7262054	56621312	20	*ANKRD60*	2.86E-08	0.038
Iodochlorohydroxyquinoline	130-26-7	rs2122382	62326484	2	*COMMD1*	2.88E-08	0.019
1-Naphthylamine	134-32-7	rs114181757	34151515	14	*NPAS3*	3.01E-08	0.04
Dichloroacetonitrile	3018-12-0	rs78571529	54350474	2	*ACYP2*	3.11E-08	0.018
Guggulsterones E	39025-24-6	rs112499908	127825897	12	*TMEM132C*	3.13E-08	0.042
Zinc pyrithione	13463-41-7	rs531928	165901231	1	*UCK2*	3.14E-08	0.042
2,2'-Thiobis(4,6-dichlorophenol)	97-18-7	rs116580451	231391030	1	*GNPAT*	3.18E-08	0.021
8-Hydroxyquinoline	148-24-3	rs4721944	20790767	7	*ABCB5*	3.23E-08	0.021
1,1,1,2-Tetrabromoethane	630-16-0	rs4566162	51322720	16	*SALL1*	3.37E-08	0.015
N-(1-Naphthyl)ethylenediamine dihydrochloride	1465-25-4	rs74706294	91849217	6	*BACH2*	3.39E-08	0.025
Acetochlor	34256-82-1	rs115237618	80040682	2	*CTNNA2*	3.40E-08	0.045
Nitazoxanide	55981-09-4	rs182482	16071020	16	*ABCC1*	3.44E-08	0.026
Colchicine	64-86-8	rs9419276	134798249	10	*C10orf93*	3.53E-08	0.047
Cycloheximide	66-81-9	rs78272463	95513038	8	*KIAA1429*	3.74E-08	0.023
Dichloroacetonitrile	3018-12-0	rs72894316	74378660	3	*CNTN3*	3.96E-08	0.018
Bisphenol A diglycidyl ether	1675-54-3	rs7708761	118856707	5	*HSD17B4*	4.01E-08	0.032
N,N-Dimethyl-p-nitrosoaniline	138-89-6	rs115237618	80040682	2	*CTNNA2*	4.02E-08	0.041

Chemical_Name	CAS #	SNP	bp[a]	Chrom	Gene	P^b	q-value[c]
Aflatoxin B1 from Aspergillus flavus	1162-65-8	rs78373020	98673048	1	*DPYD*	4.09E-08	0.017
Captan	133-06-2	rs9533891	45099224	13	*TSC22D1*	4.11E-08	0.055
7,12-Dimethylbenzanthracene	57-97-6	rs7051976	78657892	X	*ITM2A*	4.26E-08	0.046
Aflatoxin B1 from Aspergillus flavus	1162-65-8	rs73892641	2675732	20	*EBF4*	4.31E-08	0.017
m-Nitrobenzyl chloride	619-23-8	rs116563027	107358795	4	*AIMP1*	4.37E-08	0.037
1,1,1,2-Tetrabromoethane	630-16-0	rs80050627	165228913	1	*LMX1A*	4.48E-08	0.015
1,2-Epoxy-3-chloropropane (Epichlorohydrin)	106-89-8	rs10175753	230503892	2	*DNER*	4.53E-08	0.020
t-Butylhydroquinone	1948-33-0	rs76069668	101975867	2	*CREG2*	4.78E-08	0.063
17beta-Estradiol	50-28-2	rs79788122	90759826	13	*GPC5*	4.78E-08	0.028
N-(1-Naphthyl)ethylenediamine dihydrochloride	1465-25-4	rs3736146	33457697	17	*NLE1*	4.81E-08	0.025
Bisphenol A diglycidyl ether	1675-54-3	rs7699080	72885899	4	*NPFFR2*	4.83E-08	0.032
Toxaphene	8001-35-2	rs60756373	24910534	22	*UPB1*	4.84E-08	0.036
Aflatoxin B1 from Aspergillus flavus	1162-65-8	rs190899326	24419775	21	*NCAM2*	5.04E-08	0.017
4-Amino-4’-hydroxy-3-methyl-diphenylamine	6219-89-2	rs78933262	86760340	16	*FOXL1*	5.06E-08	0.034
Cycloheximide	66-81-9	rs112219638	89458222	14	*EML5*	5.06E-08	0.023
Hexachloro-1,3-butadiene	87-68-3	rs72774515	294911	2	*ACP1*	5.22E-08	0.069
N,N,N’,N’-Tetramethyl-p-phenylenediamine	100-22-1	rs74706317	150228076	2	*LYPD6*	5.32E-08	0.035
Systhane	88671-89-0	rs74798209	77038327	15	*SCAPER*	5.36E-08	0.036
2,3,4,5-Tetrachlorophenol	4901-51-3	rs1904540	67045472	12	*GRIP1*	5.39E-08	0.036
1,8-Dihydroxy-4,5-dinitroanthraquinone	81-55-0	rs58733988	37400597	4	*KIAA1239*	5.60E-08	0.025
Cadmium chloride	10108-64-2	rs4751095	131322781	10	*MGMT*	5.61E-08	0.074
m-Nitrobenzyl chloride	619-23-8	rs2570981	121765728	5	*SNCAIP*	5.63E-08	0.037
N-(1-Naphthyl)ethylenediamine dihydrochloride	1465-25-4	rs55681361	47298854	21	*PCBP3*	5.71E-08	0.025
Cycloheximide	66-81-9	rs5974707	136862247	X	*GPR101*	5.72E-08	0.038
Chlordecone (kepone)	143-50-0	rs34098910	49720932	12	*TROAP*	5.97E-08	0.040
p-Nitrosodiphenylamine	156-10-5	rs236356	36801188	6	*CPNE5*	6.00E-08	0.059
Domiphen bromide	538-71-6	rs8057753	56175242	16	*LOC283856*	6.14E-08	0.027
Titanocene dichloride	1271-19-8	rs10521479	55583383	X	*FOXR2*	6.16E-08	0.082
N,N-Dimethyl-p-nitrosoaniline	138-89-6	rs113059975	194701443	3	*C3orf21*	6.23E-08	0.041
Saquinavir mesylate	149845-06-7	rs80233769	91742937	15	*SV2B*	6.24E-08	0.083
Nitazoxanide	55981-09-4	rs7522764	147001029	1	*BCL9*	6.41E-08	0.028
4-Chloro-o-phenylenediamine	95-83-0	rs11943865	14865093	4	*CC2D2A*	6.41E-08	0.059
3-Chloro-4-(dichloromethyl)-5-hydroxy-2(5H)-furanone	77439-76-0	rs113389511	14344117	16	*MKL2*	6.43E-08	0.020
17beta-Estradiol	50-28-2	rs78468118	82700651	16	*CDH13*	6.54E-08	0.028
1-Naphthylamine	134-32-7	rs116186499	66800464	3	*LRIG1*	6.55E-08	0.044
Acetochlor	34256-82-1	rs16839696	138609221	2	*HNMT*	6.72E-08	0.045
Dazomet	533-74-4	rs114811484	140314947	2	*LRP1B*	6.79E-08	0.085
N,N'-Diphenyl-p-phenylenediamine	74-31-7	rs9523298	91953523	13	*MIR17HG*	6.79E-08	0.045
Dichloroacetonitrile	3018-12-0	rs74598282	87115833	9	*KLF4*	6.97E-08	0.021
7,12-Dimethylbenzanthracene	57-97-6	rs6681688	81881212	1	*LPHN2*	7.00E-08	0.046
Ziram	137-30-4	rs6696727	214422475	1	*SMYD2*	7.16E-08	0.095
Styrene	100-42-5	rs73154933	6944862	2	*CMPK2*	7.33E-08	0.097
Aflatoxin B1 from Aspergillus flavus	1162-65-8	rs57916829	67278617	18	*DOK6*	7.44E-08	0.020
Permethrin	52645-53-1	rs12547834	103661811	8	*KLF10*	7.66E-08	0.067
N-(1,3-Dimethylbutyl)-N’-phenyl-p-phenylenediamine	793-24-8	rs11891305	3794738	2	*ALLC*	7.75E-08	0.031
Dichloroacetonitrile	3018-12-0	rs116202592	118722702	8	*EXT1*	8.06E-08	0.021
1,3-Diiminobenz (f)-isoindoline	65558-69-2	rs115797302	71579045	5	*MRPS27*	8.89E-08	0.034
3-Chloro-4-(dichloromethyl)-5-hydroxy-2(5H)-furanone	77439-76-0	rs113073458	88348209	3	*C3orf38*	9.27E-08	0.021
Amiloride hydrochloride	2016-88-8	rs9805629	110688370	13	*COL4A1*	9.30E-08	0.062
Captan	133-06-2	rs7144942	30699518	14	*PRKD1*	9.43E-08	0.042
2,3,4,5-Tetrachlorophenol	4901-51-3	rs28483005	88832741	7	*ZNF804B*	9.81E-08	0.043
2-Amino-4-chlorophenol	95-85-2	rs7871969	6673465	9	*GLDC*	9.95E-08	0.057
Systhane	88671-89-0	rs75774128	53505943	13	*PCDH8*	9.99E-08	0.044
17beta-Estradiol	50-28-2	rs36022476	76673785	15	*SCAPER*	1.03E-07	0.028
1,3-Diiminobenz (f)-isoindoline	65558-69-2	rs75108342	186491772	4	*SORBS2*	1.03E-07	0.034

Chemical_Name	CAS #	SNP	bp[a]	Chrom	Gene	P^b	q-value[c]
Triamterene	396-01-0	rs75524003	811778	9	*DMRT1*	1.04E-07	0.075
p-Nitrosodiphenylamine	156-10-5	rs112780847	99120981	7	*ZKSCAN5*	1.06E-07	0.059
2,4-Hexadienal	142-83-6	rs76209455	838786	17	*NXN*	1.07E-07	0.071
Toxaphene	8001-35-2	rs112168443	23341969	6	*HDGFL1*	1.07E-07	0.036
Colchicine	64-86-8	rs60118770	91443259	8	*TMEM64*	1.11E-07	0.074
Nitazoxanide	55981-09-4	rs10187279	228612202	2	*SLC19A3*	1.12E-07	0.037
3-Chloro-4-(dichloromethyl)-5-hydroxy-2(5H)-furanone	77439-76-0	rs112168443	23341969	6	*HDGFL1*	1.15E-07	0.022
Captan	133-06-2	rs1793170	118150205	11	*MPZL3*	1.17E-07	0.042
2-Amino-4-chlorophenol	95-85-2	rs75408210	67608838	2	*ETAA1*	1.18E-07	0.057
Chlordecone (kepone)	143-50-0	rs9933397	55396653	16	*IRX6*	1.20E-07	0.047
4-Amino-4'-hydroxy-3-methyl-diphenylamine	6219-89-2	rs76662197	142805555	4	*IL15*	1.20E-07	0.053
Captan	133-06-2	rs35333194	123412290	6	*CLVS2*	1.27E-07	0.042
2-Amino-4-chlorophenol	95-85-2	rs77766744	40023474	12	*C12orf40*	1.31E-07	0.057
17beta-Estradiol	50-28-2	rs146986118	96895044	X	*DIAPH2*	1.33E-07	0.028
p-Nitrosodiphenylamine	156-10-5	rs114621313	216531001	2	*LOC646324*	1.33E-07	0.059
Triamterene	396-01-0	rs74000350	239858614	2	*HDAC4*	1.41E-07	0.075
17beta-Estradiol	50-28-2	rs77203508	174412976	2	*ZAK*	1.42E-07	0.028
1,2-Epoxy-3-chloropropane	106-89-8	rs77143142	104926726	12	*CHST11*	1.43E-07	0.047
N-(1,3-Dimethylbutyl)-N'-phenyl-p-phenylenediamine	793-24-8	rs114872552	71654241	17	*SDK2*	1.45E-07	0.031
2,3,4,5-Tetrachlorophenol	4901-51-3	rs74444408	27796616	14	*NOVA1*	1.51E-07	0.050
o-Aminophenol	95-55-6	rs114765730	156894511	6	*ARID1B*	1.53E-07	0.088
N-(1,3-Dimethylbutyl)-N'-phenyl-p-phenylenediamine	793-24-8	rs74831443	54356852	2	*ACYP2*	1.53E-07	0.031
Hexachloro-1,3-butadiene	87-68-3	rs72661424	103823726	13	*SLC10A2*	1.61E-07	0.071
Dichloroacetonitrile	3018-12-0	rs11215989	116407490	11	*BUD13*	1.62E-07	0.036
8-Hydroxyquinoline	148-24-3	rs2705858	14897262	2	*NBAS*	1.63E-07	0.066
1,1,1,2-Tetrabromoethane	630-16-0	rs80184146	53967078	7	*POM121L12*	1.64E-07	0.043
17beta-Estradiol	50-28-2	rs77023555	41518715	8	*MIR486*	1.67E-07	0.028
1,3-Diiminobenz (f)-isoindoline	65558-69-2	rs79099303	178107512	5	*ZNF354A*	1.68E-07	0.045
4-Chloro-o-phenylenediamine	95-83-0	rs1863773	171861497	2	*TLK1*	1.68E-07	0.059
Triamterene	396-01-0	rs80124975	55171150	7	*EGFR*	1.69E-07	0.075
N-(1,3-Dimethylbutyl)-N'-phenyl-p-phenylenediamine	793-24-8	rs73077728	39666223	5	*DAB2*	1.70E-07	0.031
Cycloheximide	66-81-9	rs938729	127996472	9	*RABEPK*	1.70E-07	0.045
2-Amino-4-chlorophenol	95-85-2	rs74445911	99188202	3	*COL8A1*	1.71E-07	0.057
Acetochlor	34256-82-1	rs75608771	181458496	4	*ODZ3*	1.73E-07	0.051
N,N'-Diphenyl-p-phenylenediamine	74-31-7	rs852350	46828160	20	*PREX1*	1.73E-07	0.057
Systhane	88671-89-0	rs76341199	73932870	6	*KHDC1L*	1.73E-07	0.057
Aflatoxin B1 from Aspergillus flavus	1162-65-8	rs73946114	115126665	2	*ACTR3*	1.73E-07	0.038
Permethrin	52645-53-1	SNP2-19172082	19308601	2	*OSR1*	1.73E-07	0.067
4-Chloro-o-phenylenediamine	95-83-0	rs35972823	127572550	9	*OLFML2A*	1.76E-07	0.059
o-Aminophenol	95-55-6	rs73367814	144496350	8	*MAFA*	1.86E-07	0.088
Acetochlor	34256-82-1	rs115850579	108846714	12	*FICD*	1.91E-07	0.051
Dichloroacetonitrile	3018-12-0	rs35516880	49704023	19	*TRPM4*	1.97E-07	0.037
o-Aminophenol	95-55-6	rs116214814	145659037	1	*RNF115*	1.98E-07	0.088
Aflatoxin B1 from Aspergillus flavus	1162-65-8	rs16863051	222617754	2	*EPHA4*	1.99E-07	0.038
Permethrin	52645-53-1	rs11879047	48535012	19	*CABP5*	2.00E-07	0.067
4-Chloro-o-phenylenediamine	95-83-0	rs915258	99654001	X	*PCDH19*	2.01E-07	0.059
1,3-Diiminobenz (f)-isoindoline	65558-69-2	rs78119276	40879472	13	*FOXO1*	2.05E-07	0.045
Chlordecone (kepone)	143-50-0	rs73490735	7839616	16	*A2BP1*	2.12E-07	0.047
p-Nitrosodiphenylamine	156-10-5	rs2697819	14073705	11	*SPON1*	2.12E-07	0.07
7,12-Dimethylbenzanthracene	57-97-6	rs77953084	130557966	3	*ATP2C1*	2.22E-07	0.074
Domiphen bromide	538-71-6	rs11110500	101123449	12	*GAS2L3*	2.23E-07	0.054
4-Chloro-o-phenylenediamine	95-83-0	rs16886866	12267803	4	*HS3ST1*	2.23E-07	0.059
Toxaphene	8001-35-2	rs74822199	92015924	1	*CDC7*	2.25E-07	0.047
Dichloroacetonitrile	3018-12-0	rs78443892	101205305	12	*ANO4*	2.34E-07	0.039
17beta-Estradiol	50-28-2	rs76272767	31321204	3	*STT3B*	2.39E-07	0.035

Chemical_Name	CAS #	SNP	bp[a]	Chrom	Gene	*P*[b]	*q*-value[c]
Domiphen bromide	538-71-6	rs77606067	12357201	2	*LPIN1*	2.41E-07	0.054
8-Hydroxyquinoline	148-24-3	rs78767283	135001510	5	*CXCL14*	2.42E-07	0.066
Domiphen bromide	538-71-6	rs77635386	97941484	14	*VRK1*	2.43E-07	0.054
1,8-Dihydroxy-4,5-dinitroanthraquinone	81-55-0	rs61749065	37921424	1	*LOC728431*	2.48E-07	0.064
2-Amino-4-chlorophenol	95-85-2	rs10980588	113609826	9	*MUSK*	2.49E-07	0.066
Chlordecone (kepone)	143-50-0	rs13229	41165878	17	*IFI35*	2.52E-07	0.047
N-Methyl-p-aminophenol sulfate	55-55-0	rs523638	234658886	1	*TARBP1*	2.54E-07	0.067
Cycloheximide	66-81-9	rs114561024	8886997	6	*SLC35B3*	2.61E-07	0.058
2,3,4,5-Tetrachlorophenol	4901-51-3	rs16930149	29601508	10	*LYZL1*	2.67E-07	0.057
p-Nitrosodiphenylamine	156-10-5	rs72884793	59742833	3	*FHIT*	2.76E-07	0.073
Toxaphene	8001-35-2	rs116409713	167134776	5	*ODZ2*	2.76E-07	0.047
Toxaphene	8001-35-2	rs9660010	240588502	1	*FMN2*	2.82E-07	0.047
Acetochlor	34256-82-1	rs114752023	49897238	2	*FSHR*	2.86E-07	0.063
2,3,4,5-Tetrachlorophenol	4901-51-3	SNP5-131756739	131728840	5	*SLC22A5*	2.95E-07	0.057
17beta-Estradiol	50-28-2	rs58049330	90611155	9	*CDK20*	2.96E-07	0.039
2,3,4,5-Tetrachlorophenol	4901-51-3	rs1052179	71267010	10	*TSPAN15*	3.01E-07	0.057
3-Chloro-4-(dichloromethyl)-5-hydroxy-2(5H)-furanone	77439-76-0	rs114377156	230451029	2	*DNER*	3.05E-07	0.038
3-Chloro-4-(dichloromethyl)-5-hydroxy-2(5H)-furanone	77439-76-0	rs72984722	140332059	2	*LRP1B*	3.10E-07	0.038
1-Naphthylamine	134-32-7	rs73742862	54763462	6	*FAM83B*	3.14E-07	0.095
Nitazoxanide	55981-09-4	rs74060294	70178204	14	*KIAA0247*	3.15E-07	0.084
Chlordecone (kepone)	143-50-0	rs1154879	42814417	14	*LRFN5*	3.16E-07	0.047
Chlordecone (kepone)	143-50-0	rs74424738	194716032	3	*C3orf21*	3.17E-07	0.047
8-Hydroxyquinoline	148-24-3	rs45507700	30782357	13	*KATNAL1*	3.18E-07	0.066
Domiphen bromide	538-71-6	rs11568403	86955535	9	*SLC28A3*	3.20E-07	0.061
Dichloroacetonitrile	3018-12-0	rs115567386	84537793	1	*TTLL7*	3.25E-07	0.043
o-Phenylenediamine	95-54-5	rs114557691	16158386	6	*MYLIP*	3.34E-07	0.087
1,3-Diiminobenz (f)-isoindoline	65558-69-2	rs79150690	23147311	9	*ELAVL2*	3.39E-07	0.064
Cycloheximide	66-81-9	rs28679586	31188401	17	*MYO1D*	3.39E-07	0.064
8-Hydroxyquinoline	148-24-3	rs12311965	131268403	12	*STX2*	3.49E-07	0.066
Progesterone	57-83-0	rs77458047	98005227	8	*PGCP*	3.54E-07	0.060
Progesterone	57-83-0	rs11214105	112037653	11	*TEX12*	3.64E-07	0.060
3-Chloro-4-(dichloromethyl)-5-hydroxy-2(5H)-furanone	77439-76-0	rs73460084	80173664	9	*GNA14*	3.67E-07	0.038
Chlordecone (kepone)	143-50-0	rs150873633	14579941	20	*MACROD2*	3.70E-07	0.049
2,3,4,5-Tetrachlorophenol	4901-51-3	rs7734259	1205226	5	*SLC6A19*	3.78E-07	0.063
Cycloheximide	66-81-9	rs7263958	12484678	20	*SPTLC3*	3.86E-07	0.064
Domiphen bromide	538-71-6	rs114377156	230451029	2	*DNER*	3.87E-07	0.062
17beta-Estradiol	50-28-2	rs16893110	24092918	5	*PRDM9*	3.89E-07	0.047
4-Chloro-o-phenylenediamine	95-83-0	rs75858454	78200496	9	*MIR548H3*	4.42E-07	0.093
1,1,1,2-Tetrabromoethane	630-16-0	rs74053705	9171488	1	*GPR157*	4.48E-07	0.085
p-Nitrosodiphenylamine	156-10-5	rs13390320	6316492	2	*SOX11*	4.49E-07	0.099
Domiphen bromide	538-71-6	rs1587323	25159133	15	*SNRPN*	4.54E-07	0.062
Progesterone	57-83-0	rs11702590	42524628	21	*BACE2*	4.55E-07	0.06
o-Phenylenediamine	95-54-5	rs76475950	54056615	2	*GPR75-ASB3*	4.56E-07	0.087
o-Phenylenediamine	95-54-5	rs16925298	7081674	9	*KDM4C*	4.58E-07	0.087
Cycloheximide	66-81-9	rs78767283	135001510	5	*CXCL14*	4.59E-07	0.068
Nitazoxanide	55981-09-4	rs12346608	280620	9	*DOCK8*	4.63E-07	0.088
Domiphen bromide	538-71-6	rs56708270	27925485	X	*DCAF8L1*	4.66E-07	0.062
1,8-Dihydroxy-4,5-dinitroanthraquinone	81-55-0	rs11860817	55388644	16	*IRX6*	4.76E-07	0.079
17beta-Estradiol	50-28-2	rs10076309	92770638	5	*FLJ42709*	4.80E-07	0.052
4-Chloro-o-phenylenediamine	95-83-0	rs3211770	113793849	13	*F10*	4.93E-07	0.093
1-Naphthylamine	134-32-7	rs114818118	79143399	7	*MAGI2*	5.02E-07	0.095
1-Naphthylamine	134-32-7	rs59993949	129924957	12	*TMEM132D*	5.07E-07	0.095
Acetochlor	34256-82-1	rs73235161	20789738	2	*HS1BP3*	5.16E-07	0.078
1-Naphthylamine	134-32-7	rs6468813	103139683	8	*NCALD*	5.29E-07	0.095
3-Chloro-4-(dichloromethyl)-5-hydroxy-2(5H)-furanone	77439-76-0	rs78748755	66503451	11	*C11orf80*	5.32E-07	0.047

Chemical_Name	CAS #	SNP	bp[a]	Chrom	Gene	*P*[b]	*q*-value[c]
Dichloroacetonitrile	3018-12-0	rs77088325	8985132	20	*PLCB1*	5.33E-07	0.059
Progesterone	57-83-0	rs7059570	26421697	X	*MAGEB6*	5.43E-07	0.060
1-Naphthylamine	134-32-7	SNP14-50902158	51832408	14	*TMX1*	5.49E-07	0.095
Acetochlor	34256-82-1	rs74088312	96597917	14	*BDKRB2*	5.51E-07	0.078
3-Chloro-4-(dichloromethyl)-5-hydroxy-2(5H)-furanone	77439-76-0	rs6540459	208369736	1	*PLXNA2*	5.64E-07	0.047
Domiphen bromide	538-71-6	rs4237673	39367004	11	*LRRC4C*	5.66E-07	0.068
Chlordecone (kepone)	143-50-0	rs73833731	56839649	3	*ARHGEF3*	5.79E-07	0.064
1,8-Dihydroxy-4,5-dinitroanthraquinone	81-55-0	rs9567646	46763028	13	*LCP1*	5.93E-07	0.087
Cycloheximide	66-81-9	rs111782938	26528764	1	*CATSPER4*	6.04E-07	0.076
Aflatoxin B1 from Aspergillus flavus	1162-65-8	rs76487712	35245296	2	*CRIM1*	6.11E-07	0.075
Acetochlor	34256-82-1	rs11110500	101123449	12	*GAS2L3*	6.47E-07	0.078
Acetochlor	34256-82-1	rs74059988	8880606	12	*RIMKLB*	6.48E-07	0.078
Nitazoxanide	55981-09-4	rs9313437	168395192	5	*SLIT3*	6.55E-07	0.097
Domiphen bromide	538-71-6	rs6028580	38282421	20	*DHX35*	6.56E-07	0.073
17beta-Estradiol	50-28-2	rs75555706	96015792	8	*C8orf38*	6.57E-07	0.058
Chlordecone (kepone)	143-50-0	rs114816993	63423482	8	*NKAIN3*	6.63E-07	0.064
Chlordecone (kepone)	143-50-0	rs73126926	53696607	5	*HSPB3*	6.73E-07	0.064
1,3-Diiminobenz (f)-isoindoline	65558-69-2	rs12813380	118296931	12	*KSR2*	6.95E-07	0.097
1-Naphthylamine	134-32-7	rs73200328	14878222	2	*NBAS*	7.05E-07	0.095
N-(1,3-Dimethylbutyl)-N'-phenyl-p-phenylenediamine	793-24-8	rs724997	173708117	2	*RAPGEF4*	7.08E-07	0.083
Cycloheximide	66-81-9	rs11130253	50681158	3	*MAPKAPK3*	7.08E-07	0.076
1-Naphthylamine	134-32-7	rs76046261	16577365	11	*C11orf58*	7.10E-07	0.095
Aflatoxin B1 from Aspergillus flavus	1162-65-8	rs115521847	149101485	3	*TM4SF1*	7.12E-07	0.075
1-Naphthylamine	134-32-7	rs55711976	42300980	19	*CEACAM3*	7.16E-07	0.095
N-(1,3-Dimethylbutyl)-N'-phenyl-p-phenylenediamine	793-24-8	rs4427625	40182606	12	*SLC2A13*	7.17E-07	0.083
1,3-Diiminobenz (f)-isoindoline	65558-69-2	rs4077144	127622908	2	*LOC339760*	7.22E-07	0.097
Aflatoxin B1 from Aspergillus flavus	1162-65-8	rs62016193	6339309	16	*RBFOX1*	7.31E-07	0.075
N-(1,3-Dimethylbutyl)-N'-phenyl-p-phenylenediamine	793-24-8	SNP16-62118696	63561195	16	*CDH11*	7.38E-07	0.083
N-(1,3-Dimethylbutyl)-N'-phenyl-p-phenylenediamine	793-24-8	rs17015753	25111128	3	*TOP2B*	7.46E-07	0.083
Chlordecone (kepone)	143-50-0	rs35972823	127572550	9	*OLFML2A*	7.90E-07	0.07
Cycloheximide	66-81-9	rs77828608	29542970	22	*KREMEN1*	8.32E-07	0.076
17beta-Estradiol	50-28-2	rs74631305	66772843	12	*GRIP1*	8.51E-07	0.071
Progesterone	57-83-0	rs11009601	34237166	10	*PARD3*	8.57E-07	0.071
Aflatoxin B1 from Aspergillus flavus	1162-65-8	rs281886	131785132	7	*PLXNA4*	8.65E-07	0.082
Cycloheximide	66-81-9	rs114442034	16159615	7	*ISPD*	8.90E-07	0.076
Cycloheximide	66-81-9	rs115317550	2476936	17	*KIAA0664*	9.15E-07	0.076
Chlordecone (kepone)	143-50-0	rs9540605	66486429	13	*PCDH9*	9.26E-07	0.070
N-(1,3-Dimethylbutyl)-N'-phenyl-p-phenylenediamine	793-24-8	rs599023	60172344	13	*DIAPH3*	9.86E-07	0.094
3-Chloro-4-(dichloromethyl)-5-hydroxy-2(5H)-furanone	77439-76-0	rs74444408	27796616	14	*NOVA1*	9.96E-07	0.075
Acetochlor	34256-82-1	rs947213	105682496	6	*PREP*	1.01E-06	0.100
Chlordecone (kepone)	143-50-0	rs9660010	240588502	1	*FMN2*	1.10E-06	0.070
Chlordecone (kepone)	143-50-0	rs11836196	16391999	12	*SLC15A5*	1.10E-06	0.070
Chlordecone (kepone)	143-50-0	rs6076084	23528536	20	*CST9L*	1.10E-06	0.070
Chlordecone (kepone)	143-50-0	rs78571529	54350474	2	*ACYP2*	1.11E-06	0.07
N-(1,3-Dimethylbutyl)-N'-phenyl-p-phenylenediamine	793-24-8	rs77953084	130557966	3	*ATP2C1*	1.13E-06	0.094
Acetochlor	34256-82-1	rs76289940	94900317	12	*TMCC3*	1.15E-06	0.100
Progesterone	57-83-0	rs77697536	19922499	2	*TTC32*	1.16E-06	0.071
3-Chloro-4-(dichloromethyl)-5-hydroxy-2(5H)-furanone	77439-76-0	rs77746073	11791200	12	*ETV6*	1.17E-06	0.080
Chlordecone (kepone)	143-50-0	rs2697819	14073705	11	*SPON1*	1.18E-06	0.071
Acetochlor	34256-82-1	rs56922398	55218460	6	*GFRAL*	1.19E-06	0.100
Chlordecone (kepone)	143-50-0	rs79304881	164845251	4	*1-Mar*	1.23E-06	0.071
Progesterone	57-83-0	rs292582	134882680	7	*WDR91*	1.25E-06	0.071
Acetochlor	34256-82-1	rs79450652	180881757	2	*CWC22*	1.25E-06	0.100
3-Chloro-4-(dichloromethyl)-5-hydroxy-2(5H)-furanone	77439-76-0	rs74631305	66772843	12	*GRIP1*	1.31E-06	0.083
Acetochlor	34256-82-1	rs58612427	68786370	2	*APLF*	1.35E-06	0.100

**Chemical_Name**	**CAS #**	**SNP**	**bp**[a]	**Chrom**	**Gene**	***P***[b]	***q*-value**[c]
Acetochlor	34256-82-1	rs73432502	22426949	13	*FGF9*	1.35E-06	0.100
3-Chloro-4-(dichloromethyl)-5-hydroxy-2(5H)-furanone	77439-76-0	rs1545116	58993226	16	*GOT2*	1.44E-06	0.083
Chlordecone (kepone)	143-50-0	rs35315427	87179	17	*RPH3AL*	1.56E-06	0.084
3-Chloro-4-(dichloromethyl)-5-hydroxy-2(5H)-furanone	77439-76-0	rs77635386	97941484	14	*VRK1*	1.69E-06	0.090
Progesterone	57-83-0	rs4758204	7619041	11	*PPFIBP2*	1.73E-06	0.084
Chlordecone (kepone)	143-50-0	rs28539080	59944606	20	*CDH4*	1.75E-06	0.087
Progesterone	57-83-0	rs8044956	70497086	16	*FUK*	1.84E-06	0.084
3-Chloro-4-(dichloromethyl)-5-hydroxy-2(5H)-furanone	77439-76-0	rs78468118	82700651	16	*CDH13*	1.90E-06	0.093
3-Chloro-4-(dichloromethyl)-5-hydroxy-2(5H)-furanone	77439-76-0	rs78297143	43604313	14	*LRFN5*	2.14E-06	0.098
Chlordecone (kepone)	143-50-0	rs9931421	77926021	16	*VAT1L*	2.19E-06	0.098
Chlordecone (kepone)	143-50-0	rs7868175	6658080	9	*GLDC*	2.56E-06	0.098
Chlordecone (kepone)	143-50-0	rs2012691	105453745	7	*ATXN7L1*	2.62E-06	0.098
Chlordecone (kepone)	143-50-0	rs74059988	8880606	12	*RIMKLB*	2.71E-06	0.098
Chlordecone (kepone)	143-50-0	rs73808311	3349809	3	*CRBN*	2.75E-06	0.098
Chlordecone (kepone)	143-50-0	rs61214761	114039909	7	*FOXP2*	2.75E-06	0.098
Chlordecone (kepone)	143-50-0	rs56381502	25845922	5	*CDH9*	2.97E-06	0.098
Chlordecone (kepone)	143-50-0	rs79494514	34122346	13	*STARD13*	2.97E-06	0.098
Chlordecone (kepone)	143-50-0	rs79843432	239514826	1	*FMN2*	2.99E-06	0.098
Chlordecone (kepone)	143-50-0	rs73145960	5192099	2	*SOX11*	3.02E-06	0.098

[a]NCBI build 37. [b]*P*-value. [c]FDRc2dw fv *q*-value was obtained per chemical.

Table S6. Significant EC_{10} –SNP associations among larger set of 12m SNPs.

Chemical_Name	CAS #	SNP	bp[a]	Chrom	Gene[b]	*P*[c]	*q* value
Vitamin D3	67-97-0	rs75591162	179520663	3	*PEX5L*	2.79E-14	3.47E-07
Amiloride hydrochloride	2016-88-8	rs149885464	114388490	X	*LRCH2*	9.75E-14	1.21E-06
Systhane	88671-89-0	rs114097262	57530631	20	*TH1L*	2.22E-12	2.76E-05
Cycloheximide	66-81-9	rs112895158	143565653	3	*C3orf58*	3.19E-12	3.96E-05
Hexachlorophene	70-30-4	rs149041648	4081594	7	*SDK1*	3.88E-12	4.82E-05
17beta-Estradiol	50-28-2	rs148557261	85025463	X	*CHM*	4.44E-12	2.76E-05
1,8-Dihydroxy-4,5-dinitroanthraquinone	81-55-0	rs115918434	70712674	6	*COL19A1*	5.56E-12	6.90E-05
o-Nitrobenzyl chloride	612-23-7	rs9966852	20953234	18	*C18orf45*	9.72E-12	1.00E-04
Cycloheximide	66-81-9	rs116560380	53916956	16	*FTO*	1.70E-11	1.00E-04
1,3-Diiminobenz (f)-isoindoline	65558-69-2	rs150407211	204994131	1	*NFASC*	1.82E-11	2.00E-04
N-Methyl-p-aminophenol sulfate	55-55-0	rs139684082	55659431	20	*BMP7*	1.88E-11	2.00E-04
2,2'-Thiobis(4,6-dichlorophenol)	97-18-7	rs78022668	220093749	1	*EPRS*	2.19E-11	9.06E-05
17beta-Estradiol	50-28-2	rs114097262	57530631	20	*TH1L*	2.90E-11	6.86E-05
1,1,1,2-Tetrabromoethane	630-16-0	rs116253534	7734317	3	*GRM7*	3.24E-11	4.00E-04
Saquinavir mesylate	149845-06-7	rs77326389	27959745	15	*OCA2*	3.77E-11	5.00E-04
Nitazoxanide	55981-09-4	rs116345917	38690860	5	*LIFR*	4.41E-11	3.91E-05
Dichlorvos	62-73-7	rs5915692	4004014	X	*PRKX*	5.19E-11	6.00E-04
17beta-Estradiol	50-28-2	rs77919593	178511146	2	*PDE11A*	5.52E-11	6.86E-05
Ziram	137-30-4	rs114456684	89021170	13	*SLITRK5*	6.92E-11	9.00E-04
cis-Dichlorodiamine platinum	15663-27-1	rs13236745	21072550	7	*SP8*	7.19E-11	3.00E-04
Nitazoxanide	55981-09-4	rs114256919	106718263	14	*LOC10028856[illegible]*	7.81E-11	6.06E-05
1,6-Hexamethylene diacrylate	13048-33-4	rs62231930	24939620	22	*C22orf13*	7.91E-11	0.001
N,N,N',N'-Tetramethyl-p-phenylenediamine	100-22-1	rs111412077	92398645	11	*FAT3*	9.36E-11	5.00E-04
p-Nitrosodiphenylamine	156-10-5	rs78789459	188016223	4	*FAT1*	1.04E-10	7.00E-04
Hexachlorophene	70-30-4	rs185960291	107817291	11	*RAB39*	1.11E-10	7.00E-04
p-Nitrosodiphenylamine	156-10-5	rs185480677	214584272	1	*PTPN14*	1.14E-10	7.00E-04
17beta-Estradiol	50-28-2	rs77023555	41518715	8	*ANK1*	1.20E-10	1.00E-04
1-Methyl-3-nitro-1-nitroso-guanidine	81-55-0	rs139598440	89777354	X	*TGIF2LX*	1.22E-10	5.00E-04
1,8-Dihydroxy-4,5-dinitroanthraquinone	70-25-7	rs115531561	69465188	2	*ANTXR1*	1.22E-10	0.002
o-Aminophenol	95-55-6	rs182028010	230236320	1	*GALNT2*	1.26E-10	0.002
1,3-Diiminobenz (f)-isoindoline	65558-69-2	rs146270840	194601175	3	*FAM43A*	1.56E-10	6.00E-04
Glutaraldehyde	111-30-8	rs7992282	31601586	13	*C13orf26*	1.58E-10	0.002
Nitazoxanide	55981-09-4	rs182761810	47129104	X	*USP11*	1.59E-10	1.00E-04
4-Chloro-o-phenylenediamine	95-83-0	rs146160738	103390555	X	*MCART6*	1.68E-10	0.002
Systhane	88671-89-0	rs111763750	53503234	13	*PCDH8*	1.72E-10	3.00E-04
6-Thioguanine	154-42-7	rs72484656	73259654	9	*TRPM3*	1.99E-10	0.003
1,3-Diiminobenz (f)-isoindoline	65558-69-2	rs148557261	85025463	X	*CHM*	2.02E-10	6.00E-04
Vitamin D3	67-97-0	rs79427953	155449132	5	*SGCD*	2.31E-10	9.00E-04
p-Quinone	106-51-4	rs140356758	96472953	13	*UGGT2*	2.35E-10	0.003
Vitamin D3	67-97-0	rs73001915	180641510	4	*LOC285501*	2.47E-10	9.00E-04
4-Chloro-o-phenylenediamine	95-83-0	rs61790870	34693156	4	*ARAP2*	2.53E-10	0.002
17beta-Estradiol	50-28-2	rs138873795	93861771	15	*RGMA*	2.59E-10	3.00E-04
Retinal	116-31-4	rs61741388	114156579	9	*KIAA0368*	3.11E-10	0.002
17beta-Estradiol	50-28-2	rs9957628	57478543	18	*PMAIP1*	3.18E-10	3.00E-04
1,2-Epoxy-3-chloropropane	106-89-8	rs114097262	57530631	20	*TH1L*	3.35E-10	0.004
Vitamin D3	67-97-0	rs77571603	29323478	10	*LYZL1*	3.37E-10	9.00E-04
Chlordecone (kepone)	143-50-0	rs6834412	4731136	4	*MSX1*	3.58E-10	0.003
Cycloheximide	66-81-9	rs4075856	127996778	9	*RABEPK*	3.66E-10	8.00E-04
N,N,N',N'-Tetramethyl-p-phenylenediamine	100-22-1	rs139345780	35242366	2	*CRIM1*	3.73E-10	0.001

**Chemical_Name**	**CAS #**	**SNP**	**bp[a]**	**Chrom**	**Gene[b]**	***P*[c]**	***q* value**
Vitamin D3	67-97-0	rs140124941	58119464	12	*AGAP2*	3.75E-10	9.00E-04
Sodium dichromate dihydrate (VI)	7789-12-0	rs144044660	175439270	4	*HPGD*	3.76E-10	0.005
Systhane	88671-89-0	rs182761810	47129104	X	*USP11*	3.79E-10	5.00E-04
Azathioprine	446-86-6	rs72625563	30966462	X	*TAB3*	3.90E-10	0.004
2',4',5'-Trihydroxybutyrophenone	1421-63-2	rs77671255	18038964	7	*PRPS1L1*	4.15E-10	0.005
Retinol acetate	127-47-9	rs115240733	83143062	8	*SNX16*	4.28E-10	0.005
N,N'-Diphenyl-p-phenylenediamine	74-31-7	rs150553218	180241673	4	*LOC285501*	4.57E-10	5.00E-04
1,3-Diiminobenz (f)-isoindoline	65558-69-2	rs79373644	68284871	17	*KCNJ2*	4.60E-10	0.001
N,N-Diethyl-p-phenylenediamine	93-05-0	rs184971028	23937090	9	*ELAVL2*	4.63E-10	0.006
Nifedipine	21829-25-4	rs74383932	211608006	2	*CPS1*	4.76E-10	0.002
o-Nitrobenzyl chloride	612-23-7	rs75820586	88320786	14	*GALC*	4.89E-10	0.003
17beta-Estradiol	50-28-2	rs114750717	143943362	X	*SPANXN1*	4.99E-10	4.00E-04
Guggulsterones E	39025-24-6	rs74929760	146457160	4	*MMAA*	5.00E-10	0.006
Hydroquinone	123-31-9	rs78268501	7128396	4	*SORCS2*	5.03E-10	0.006
Endosulfan	115-29-7	rs75512317	132143171	X	*USP26*	5.16E-10	0.006
2,2'-Thiobis(4,6-dichlorophenol)	97-18-7	rs76656117	24217993	10	*KIAA1217*	5.29E-10	0.002
Nifedipine	21829-25-4	rs187575156	33840771	16	*ZNF267*	5.51E-10	0.002
Hexachlorophene	70-30-4	rs188272213	38447642	5	*EGFLAM*	5.59E-10	0.002
Nitazoxanide	55981-09-4	rs115715858	88034425	15	*AGBL1*	6.03E-10	3.00E-04
Rhein (1,8-dihydroxy-3-carboxyl anthraquinone)	478-43-3	rs181686881	28901928	15	*GOLGA8F*	6.04E-10	0.008
Mercuric chloride	7487-94-7	rs115872305	65584758	5	*SFRS12*	6.05E-10	0.008
Saquinavir mesylate	50-28-2	rs115129751	102066268	14	*DIO3*	6.16E-10	5.00E-04
17beta-Estradiol	149845-06-7	rs28712763	38664057	5	*LIFR*	6.16E-10	0.002
Chlordecone (kepone)	143-50-0	rs28502033	71440392	7	*WBSCR17*	6.28E-10	0.003
1,8-Dihydroxy-4,5-dinitroanthraquinone	81-55-0	rs186751172	137938171	X	*FGF13*	7.36E-10	0.001
1-Naphthylamine	134-32-7	rs56166709	26718141	16	*JMJD5*	7.81E-10	0.002
Oxymetholone	434-07-1	rs118085300	72050372	10	*NPFFR1*	7.90E-10	0.010
1-Naphthylamine	134-32-7	rs148976999	89292543	2	*EIF2AK3*	7.96E-10	0.002
Fumaronitrile	764-42-1	rs112841261	95693291	8	*ESRP1*	8.09E-10	0.010
Nitazoxanide	55981-09-4	rs139729800	176909149	1	*ASTN1*	8.33E-10	3.00E-04
Pentaerythritol triacrylate	3524-68-3	rs12263759	127748420	10	*ADAM12*	8.37E-10	0.007
Cycloheximide	66-81-9	rs2774024	21573881	1	*ECE1*	8.42E-10	9.00E-04
Saquinavir mesylate	149845-06-7	rs115362446	59318384	10	*IPMK*	8.50E-10	0.002
9-Aminoacridine, monohydrochloride, monohydrate	52417-22-8	rs148846460	115474911	X	*SLC6A14*	8.52E-10	0.011
Saquinavir mesylate	149845-06-7	rs140911187	32806643	11	*CCDC73*	8.61E-10	0.002
p-Nitrosodiphenylamine	156-10-5	rs115484855	116269294	10	*ABLIM1*	8.63E-10	0.002
2,2',4'-Trichloroacetophenone	4252-78-2	rs73371185	3098435	18	*MYOM1*	8.84E-10	0.011
Sodium dichromate dihydrate (VI)	7789-12-0	rs117147263	3894712	12	*PARP11*	9.07E-10	0.006
1,1,1,2-Tetrabromoethane	630-16-0	rs73561088	87780267	13	*SLITRK5*	9.11E-10	0.002
Azathioprine	446-86-6	rs191755607	40475255	X	*ATP6AP2*	9.13E-10	0.004
Retinal	116-31-4	rs113516752	106955309	14	*LOC100288568*	9.20E-10	0.002
Retinal	116-31-4	rs115605961	36196063	3	*STAC*	9.46E-10	0.002
o-Phenylenediamine	95-54-5	rs78961197	55949853	19	*SHISA7*	9.46E-10	0.006
p-Aminophenol	123-30-8	rs113103160	107838962	11	*RAB39*	9.50E-10	0.007
2,4-Hexadienal	142-83-6	rs79177535	114025195	11	*ZBTB16*	9.74E-10	0.012
Ziram	137-30-4	rs116855907	21587330	16	*METTL9*	1.00E-09	0.006
Diethylene glycol diacrylate	4074-88-8	rs78140462	110470359	12	*ANKRD13A*	1.02E-09	0.013
Cetylpyridinium bromide	140-72-7	rs80257852	23561114	9	*ELAVL2*	1.03E-09	0.013
Azathioprine	3018-12-0	rs28365025	183643255	3	*ABCC5*	1.06E-09	0.003
Dichloroacetonitrile	446-86-6	rs117910051	164141498	6	*PACRG*	1.06E-09	0.004
1,1,1,2-Tetrabromoethane	630-16-0	rs142817312	110092992	10	*SORCS1*	1.09E-09	0.002

Chemical_Name	CAS #	SNP	bp[a]	Chrom	Gene[b]	*P*[c]	*q* value
Saquinavir mesylate	149845-06-7	rs145544371	6472066	X	*VCX3A*	1.10E-09	0.002
Colchicine	64-86-8	rs80328096	19803987	3	*EFHB*	1.11E-09	0.002
Pentaerythritol triacrylate	3524-68-3	rs192972674	72721640	X	*PABPC1L2A*	1.11E-09	0.007
2,3,4,5-Tetrachloronitrobenzene	879-39-0	rs115782131	85339219	2	*TCF7L1*	1.13E-09	0.014
p-Nitrosodiphenylamine	50-28-2	rs12087685	23727348	1	*TCEA3*	1.15E-09	8.00E-04
17beta-Estradiol	156-10-5	rs193048198	243945733	1	*ZNF238*	1.15E-09	0.002
Phenformin hydrochloride	834-28-6	rs140883532	53503005	20	*CYP24A1*	1.16E-09	0.014
Azathioprine	446-86-6	rs146213067	136068920	X	*GPR101*	1.17E-09	0.004
p-Aminophenol	123-30-8	rs57325800	10214905	1	*UBE4B*	1.19E-09	0.007
1,3,5-Triglycidyl isocyanurate	2451-62-9	rs150927141	11429085	1	*UBIAD1*	1.19E-09	0.007
Tetramethylthiouram disulfide	100-27-6	rs140556470	48843069	X	*GRIPAP1*	1.20E-09	0.004
p-Nitrophenethyl alcohol	137-26-8	rs142624309	58608899	1	*OMA1*	1.20E-09	0.012
Progesterone	143-50-0	rs185665429	129727410	8	*MYC*	1.22E-09	0.003
Chlordecone (kepone)	57-83-0	rs73285294	153826706	5	*SAP30L*	1.22E-09	0.015
17beta-Estradiol	50-28-2	rs76191860	116013224	12	*MED13L*	1.24E-09	8.00E-04
Retinol acetate	127-47-9	rs7838214	118761053	8	*EXT1*	1.29E-09	0.008
1,2-Epoxy-3-chloropropane	106-89-8	rs77143142	104926726	12	*TXNRD1*	1.32E-09	0.005
o-Phenylenediamine	88671-89-0	rs11917950	8725408	3	*C3orf32*	1.37E-09	0.002
Systhane	95-54-5	rs138701165	33431943	X	*DMD*	1.37E-09	0.006
1,1,1,2-Tetrabromoethane	630-16-0	rs113917611	145680395	5	*RBM27*	1.41E-09	0.002
Ergotamine tartrate	379-79-3	rs78908092	91139027	8	*CALB1*	1.42E-09	0.002
Nitrogen mustard hydrochloride	55-86-7	rs185629858	72754141	9	*MAMDC2*	1.44E-09	0.009
Nitrogen mustard hydrochloride	55-86-7	rs150079115	229475297	1	*C1orf96*	1.46E-09	0.009
Triamterene	396-01-0	rs116253534	7734317	3	*GRM7*	1.47E-09	0.018
t-Butylhydroquinone	1948-33-0	rs78839292	4302598	6	*PECI*	1.48E-09	0.018
Pyrimethamine	58-14-0	rs150650924	19223048	16	*SYT17*	1.53E-09	0.019
1,2-Epoxy-3-chloropropane	106-89-8	rs73380687	33444259	15	*FMN1*	1.56E-09	0.005
Dichloroacetonitrile	3018-12-0	rs116561096	173810265	4	*GALNT7*	1.57E-09	0.004
Chlordecone (kepone)	143-50-0	rs189105020	53254392	X	*IQSEC2*	1.60E-09	0.003
1,1,1,2-Tetrabromoethane	630-16-0	rs113557926	37267705	2	*HEATR5B*	1.61E-09	0.002
2-Chloroacetophenone	532-27-4	rs138236656	17144946	17	*FLCN*	1.63E-09	0.020
p-Nitrophenethyl alcohol	100-27-6	rs74821785	180732042	4	*LOC285501*	1.65E-09	0.004
Diisobutyl phthalate	84-69-5	rs59163027	3618869	8	*CSMD1*	1.71E-09	0.021
Systhane	88671-89-0	rs114062278	67964362	5	*PIK3R1*	1.75E-09	0.002
Chlordecone (kepone)	143-50-0	rs147841840	121413130	12	*HNF1A*	1.76E-09	0.003
Toxaphene	8001-35-2	rs2232859	24890981	22	*UPB1*	1.77E-09	0.021
Dexamethazone	50-02-2	rs148291248	94417682	1	*ABCA4*	1.77E-09	0.022
2-Octyl-3-isothiazolone	26530-20-1	rs114113654	104765328	2	*POU3F3*	1.83E-09	0.023
Vitamin D3	67-97-0	rs115033986	35362684	2	*CRIM1*	1.87E-09	0.002
Vitamin D3	67-97-0	rs76371158	87850932	11	*RAB38*	1.90E-09	0.002
Amiloride hydrochloride	2016-88-8	rs138475390	144438110	X	*SPANXN1*	1.94E-09	0.008
3,4-Diaminotoluene	496-72-0	rs187884265	30037137	X	*MAGEB2*	1.94E-09	0.024
Endosulfan	115-29-7	rs141860023	147034359	X	*FMR1*	1.98E-09	0.012
Tetramethylthiouram disulfide	137-26-8	rs189210853	100196239	1	*FRRS1*	1.98E-09	0.012
2',4',5'-Trihydroxybutyrophenone	1421-63-2	rs10213832	172302409	5	*ERGIC1*	1.98E-09	0.012
Diethylene glycol diacrylate	149845-06-7	rs76663109	219066443	1	*LYPLAL1*	2.01E-09	0.004
Saquinavir mesylate	4074-88-8	rs34600565	51549528	15	*CYP19A1*	2.01E-09	0.013
1,3-Diiminobenz (f)-isoindoline	65558-69-2	rs184897828	23101302	7	*KLHL7*	2.02E-09	0.003
Hydroquinone	123-31-9	rs259613	238432583	1	*ZP4*	2.05E-09	0.013
Systhane	88671-89-0	rs115987646	17518759	21	*USP25*	2.08E-09	0.002
Vitamin D3	67-97-0	rs114742274	78274036	9	*PCSK5*	2.11E-09	0.002
Hexachlorophene	70-30-4	rs4599696	72213159	7	*TYW1B*	2.16E-09	0.007
p-Nitrosodiphenylamine	156-10-5	rs76022962	57713812	16	*GPR97*	2.19E-09	0.003
1-Naphthylamine	143-50-0	rs79365910	127437542	5	*SLC12A2*	2.28E-09	0.003

**Chemical_Name**	**CAS #**	**SNP**	**bp**[a]	**Chrom**	**Gene**[b]	***P***[c]	***q* value**
Chlordecone (kepone)	134-32-7	rs73982303	20719652	17	*CCDC144NL*	2.28E-09	0.003
Fumaronitrile	764-42-1	rs150469316	38260872	15	*TMCO5A*	2.33E-09	0.015
13-cis-Retinal	472-86-6	rs141310758	57507867	20	*GNAS*	2.35E-09	0.004
N,N-Diethyl-p-phenylenediamine	93-05-0	rs141821443	78500126	16	*WWOX*	2.36E-09	0.015
Sulfathiazole	72-14-0	rs192596112	18253562	17	*SHMT1*	2.37E-09	0.023
Malachite green oxalate	2437-29-8	rs77904586	3698213	3	*LRRN1*	2.38E-09	0.018
Dibromonitromethane	598-91-4	rs139080195	33110833	6	*COL11A2*	2.51E-09	0.005
Pentaerythritol triacrylate	3524-68-3	rs114097262	57530631	20	*TH1L*	2.56E-09	0.011
Aldicarb	116-06-3	rs117120562	76067201	7	*ZP3*	2.59E-09	0.032
Melatonin	472-86-6	rs5030075	186456754	3	*KNG1*	2.63E-09	0.004
13-cis-Retinal	73-31-4	rs146412275	190261780	3	*TMEM207*	2.63E-09	0.033
Retinol acetate	127-47-9	rs112136721	76923060	1	*ST6GALNAC3*	2.71E-09	0.008
Captan	133-06-2	rs80260839	55452081	11	*OR4C6*	2.72E-09	0.018
Nitazoxanide	55981-09-4	rs112047343	25684936	6	*SCGN*	2.75E-09	9.00E-04
Vitamin D3	67-97-0	rs16908781	122453569	9	*DBC1*	2.76E-09	0.002
Chlorhexidine	55-56-1	rs2069493	99967186	14	*CCNK*	2.78E-09	0.035
2-Amino-4-chlorophenol	95-85-2	rs149669986	188717980	4	*TRIML2*	2.82E-09	0.032
Flutamide	13311-84-7	rs147788105	36895798	14	*SFTA3*	2.92E-09	0.036
8-Hydroxyquinoline	148-24-3	rs190163116	14927274	2	*NBAS*	2.93E-09	0.036
p-Nitrosodiphenylamine	156-10-5	rs142249979	22127356	3	*ZNF385D*	2.96E-09	0.003
p-Nitrosodiphenylamine	156-10-5	rs139756912	53520212	19	*ZNF160*	3.02E-09	0.003
13-cis-Retinal	472-86-6	rs112109406	41635317	19	*CYP2F1*	3.06E-09	0.004
Mono(2-ethylhexyl)phthalate	4376-20-9	rs142849625	87323965	X	*KLHL4*	3.08E-09	0.038
o-Phenylenediamine	95-54-5	rs78673081	159546875	6	*FNDC1*	3.14E-09	0.006
2-Chloroacetophenone	532-27-4	rs7885074	79470548	X	*TBX22*	3.15E-09	0.020
Nitazoxanide	55981-09-4	rs147784707	39731980	19	*IL28B*	3.22E-09	0.001
1,2-Epoxy-3-chloropropane (Epichlorohydrin)	106-89-8	rs113130251	16684250	8	*FGF20*	3.24E-09	0.008
Dichloroacetonitrile	3018-12-0	rs17517181	132048205	9	*C9orf106*	3.25E-09	0.004
Dichloroacetonitrile	3018-12-0	rs116202592	118722702	8	*EXT1*	3.25E-09	0.004
Retinal	116-31-4	rs75551402	144924136	6	*UTRN*	3.25E-09	0.005
Hydroquinone	123-31-9	rs141081361	12301484	6	*EDN1*	3.36E-09	0.014
Mercuric chloride	7487-94-7	rs114980796	146855180	7	*CNTNAP2*	3.44E-09	0.012
1,1,1,2-Tetrabromoethane	630-16-0	rs76616047	165230707	1	*LMX1A*	3.49E-09	0.003
Chlordecone (kepone)	143-50-0	rs117340306	159702181	6	*FNDC1*	3.49E-09	0.041
Cycloheximide	74-31-7	rs140838976	42607192	2	*EML4*	3.50E-09	0.001
N,N'-Diphenyl-p-phenylenediamine	66-81-9	rs141347833	95509996	8	*KIAA1429*	3.50E-09	0.003
1,3-Diiminobenz (f)-isoindoline	65558-69-2	rs115797302	71579045	5	*MRPS27*	3.56E-09	0.003
Nitazoxanide	55981-09-4	rs9587366	108038145	13	*FAM155A*	3.58E-09	0.001
Nitazoxanide	55981-09-4	rs112507751	13672142	7	*ETV1*	3.59E-09	0.001
1,2-Epoxy-3-chloropropane	106-89-8	rs143124581	127508299	2	*GYPC*	3.66E-09	0.008
Chlordecone (kepone)	143-50-0	rs9305010	23959373	19	*ZNF681*	3.69E-09	0.003
Chlorambucil	100-22-1	rs73477286	10512356	X	*CLCN4*	3.73E-09	0.008
N,N,N',N'-Tetramethyl-p-phenylenediamine	305-03-3	rs148476652	230219445	2	*DNER*	3.73E-09	0.024
HC blue 2	33229-34-4	rs150501661	80565923	2	*CTNNA2*	3.78E-09	0.047
Cycloheximide	66-81-9	rs77426543	23327356	14	*MMP14*	3.86E-09	0.003
p-Nitrophenethyl alcohol	100-27-6	rs7235815	42824095	18	*SETBP1*	3.91E-09	0.008
Methylene bis(thiocyanate)	6317-18-6	rs111371582	200201474	1	*FAM58B*	3.94E-09	0.049
Azathioprine	446-86-6	rs145504708	104046255	14	*C14orf153*	3.97E-09	0.008
1,8-Dihydroxy-4,5-dinitroanthraquinone	81-55-0	rs58965029	55366235	7	*LANCL2*	4.02E-09	0.002
2,2',4'-Trichloroacetophenone	4252-78-2	rs149411402	24732313	X	*POLA1*	4.05E-09	0.025
Retinol acetate	127-47-9	rs7220141	8596496	17	*CCDC42*	4.18E-09	0.008

Chemical_Name	CAS #	SNP	bp[a]	Chrom	Gene[b]	*P*[c]	*q* value
Glutaraldehyde	111-30-8	rs17080050	179587743	5	*RASGEF1C*	4.22E-09	0.005
9-Aminoacridine, monohydrochloride, monohydrate	52417-22-8	rs143574721	71726068	4	*GRSF1*	4.23E-09	0.019
Dichloroacetonitrile	3018-12-0	rs116306510	9045458	20	*PLCB1*	4.25E-09	0.004
Saquinavir mesylate	149845-06-7	rs148557261	85025463	X	*CHM*	4.30E-09	0.005
1,3-Diiminobenz (f)-isoindoline	65558-69-2	rs1937167	121239310	X	*GLUD2*	4.32E-09	0.003
4-Chloro-3,5-dinitro-a,a,a-trifluorotoluene	393-75-9	rs6784365	16264220	3	*GALNTL2*	4.37E-09	0.009
Phenylmercuric acetate	62-38-4	rs183322569	144144565	X	*SPANXN1*	4.38E-09	0.054
1,3-Dicyclohexylcarbodiimide	538-75-0	rs185941966	48916163	X	*CCDC120*	4.42E-09	0.055
Ergotamine tartrate	379-79-3	rs7060812	143883587	X	*SPANXN1*	4.45E-09	0.002
1-Naphthylamine	134-32-7	rs76224072	66811251	3	*KBTBD8*	4.45E-09	0.005
Captan	133-06-2	rs117766494	134786971	10	*C10orf93*	4.55E-09	0.029
Vitamin D3	67-97-0	rs852471	5679853	7	*FSCN1*	4.61E-09	0.003
1,1,1,2-Tetrabromoethane	630-16-0	rs75201540	107697197	3	*CD47*	4.63E-09	0.003
9-Aminoacridine, monohydrochloride, monohydrate	52417-22-8	rs150993793	107634677	6	*PDSS2*	4.64E-09	0.019
Captan	133-06-2	rs143629216	108864949	X	*KCNE1L*	4.69E-09	0.029
Saquinavir mesylate	149845-06-7	rs77216777	7737628	16	*A2BP1*	4.74E-09	0.005
Sodium dichromate dihydrate (VI)	7789-12-0	rs76202242	77000874	15	*SCAPER*	4.80E-09	0.020
p-Nitrosodiphenylamine	156-10-5	rs13390320	6316492	2	*SOX11*	4.87E-09	0.004
2-Amino-4-chlorophenol	95-85-2	rs114488597	6692895	9	*GLDC*	5.09E-09	0.032
Tamoxifen citrate	54965-24-1	rs73418488	39876321	22	*MGAT3*	5.11E-09	0.017
Chlordecone (kepone)	143-50-0	rs7604210	220464122	2	*STK11IP*	5.14E-09	0.004
17beta-Estradiol	50-28-2	rs73515607	12081555	16	*TNFRSF17*	5.16E-09	0.002
Nitazoxanide	55981-09-4	rs142350989	11163875	18	*FAM38B*	5.18E-09	0.002
Glutaraldehyde	111-30-8	rs145788790	76706431	3	*ZNF717*	5.26E-09	0.005
o-Phenylenediamine	95-54-5	rs10487606	24377991	7	*NPY*	5.27E-09	0.008
Tetrachlorvinphos	961-11-5	rs17037561	108142257	4	*DKK2*	5.40E-09	0.065
Endosulfan	115-29-7	rs77954654	28003512	7	*JAZF1*	5.42E-09	0.022
Turmeric	458-37-7	rs151050814	3717523	2	*ALLC*	5.47E-09	0.068
Chlordecone (kepone)	143-50-0	rs114366607	9548336	11	*ZNF143*	5.48E-09	0.004
17beta-Estradiol	50-28-2	rs78044298	13282331	17	*HS3ST3A1*	5.61E-09	0.002
17beta-Estradiol	50-28-2	rs73400780	100613871	7	*MUC12*	5.62E-09	0.002
p-Nitrosodiphenylamine	156-10-5	rs8056610	80414263	16	*DYNLRB2*	5.69E-09	0.004
1,1,1,2-Tetrabromoethane	630-16-0	rs114472896	51352245	16	*SALL1*	5.70E-09	0.004
Azobenzene	103-33-3	rs8034597	50564358	15	*HDC*	5.81E-09	0.022
Catechol	120-80-9	rs5970515	150169694	X	*HMGB3*	6.01E-09	0.044
Dichlorvos	62-73-7	rs147257714	119289133	4	*PRSS12*	6.07E-09	0.025
Alizarin Yellow R, free acid	2243-76-7	rs9484620	142617993	6	*GPR126*	6.08E-09	0.076
p-Benzoquinone dioxime	105-11-3	rs116199788	14629043	9	*ZDHHC21*	6.09E-09	0.025
Digoxin	20830-75-5	rs4668546	7802776	2	*RNF144A*	6.17E-09	0.033
Retinol acetate	127-47-9	rs77518266	16948889	9	*CNTLN*	6.18E-09	0.008
Vitamin D3	67-97-0	rs147712607	131033194	12	*RIMBP2*	6.21E-09	0.003
o-Phenylenediamine	95-54-5	rs10197500	6314210	2	*SOX11*	6.24E-09	0.009
Tamoxifen citrate	54965-24-1	rs140206324	162533710	3	*OTOL1*	6.24E-09	0.017
1,3-Diiminobenz (f)-isoindoline	65558-69-2	rs111425337	45991437	20	*ZMYND8*	6.25E-09	0.003
4-Chloro-3,5-dinitro-a,a,a-trifluorotoluene	393-75-9	rs73317253	111956225	8	*KCNV1*	6.27E-09	0.009
Vitamin D3	67-97-0	rs114278053	41810548	14	*LRFN5*	6.28E-09	0.003
p-Nitrophenethyl alcohol	100-27-6	rs114585626	6905318	16	*A2BP1*	6.28E-09	0.009
1-Naphthylamine	134-32-7	rs116257974	83616924	7	*SEMA3A*	6.31E-09	0.005
Nitazoxanide	55981-09-4	rs16887170	55652860	6	*BMP5*	6.34E-09	0.002
Mono(2-ethylhexyl)phthalate	4376-20-9	rs140233380	150658923	X	*PASD1*	6.35E-09	0.039
1,1,1,2-Tetrabromoethane	630-16-0	rs112605190	74299348	17	*QRICH2*	6.48E-09	0.004

Chemical_Name	CAS #	SNP	bp[a]	Chrom	Gene[b]	*P*[c]	*q* value
Bisphenol A diglycidyl ether	1675-54-3	rs189546210	89544224	2	*EIF2AK3*	6.52E-09	0.077
Cycloheximide	66-81-9	rs59128236	187624402	3	*BCL6*	6.60E-09	0.003
1,1,1,2-Tetrabromoethane	630-16-0	rs77326389	27959745	15	*OCA2*	6.70E-09	0.004
Rhein (1,8-dihydroxy-3-carboxyl anthraquinone)	478-43-3	rs80120215	119009775	1	*SPAG17*	6.70E-09	0.042
Nifedipine	21829-25-4	rs148079596	74646587	13	*KLF12*	6.73E-09	0.017
Cetylpyridinium bromide	140-72-7	rs192455865	77187063	5	*AP3B1*	6.73E-09	0.030
1,8-Dihydroxy-4,5-dinitroanthraquinone	81-55-0	rs190416563	40716108	20	*PTPRT*	6.74E-09	0.003
1,3-Diiminobenz (f)-isoindoline	65558-69-2	rs7214324	75241094	17	*SEC14L1*	6.74E-09	0.003
Methyl mercuric (II) chloride	115-09-3	rs140638640	3157831	X	*MXRA5*	6.96E-09	0.059
1,3-Diiminobenz (f)-isoindoline	65558-69-2	rs114723141	71915908	18	*CYB5A*	7.06E-09	0.003
1,2-Epoxy-3-chloropropane	3018-12-0	rs16958628	11860133	16	*ZC3H7A*	7.08E-09	0.004
Dichloroacetonitrile	106-89-8	rs148108647	388033	7	*FAM20C*	7.08E-09	0.011
Captan	133-06-2	rs143336822	79139911	15	*MORF4L1*	7.10E-09	0.018
Catechol	120-80-9	rs75909882	186181894	3	*CRYGS*	7.16E-09	0.044
Saquinavir mesylate	149845-06-7	rs139792645	69525002	14	*DCAF5*	7.20E-09	0.006
Flutamide	13311-84-7	rs75591162	179520663	3	*PEX5L*	7.30E-09	0.039
4-Chloro-3,5-dinitro-a,a,a-trifluorotoluene	393-75-9	rs140966476	1185683	19	*STK11*	7.32E-09	0.009
4-Chloro-o-phenylenediamine	95-83-0	rs114170015	8036026	3	*GRM7*	7.42E-09	0.023
Aldicarb	116-06-3	rs77630313	67951530	2	*C1D*	7.43E-09	0.046
2,3,5-Trichlorophenol	933-78-8	rs72743494	210497480	1	*HHAT*	7.50E-09	0.093
Retinol acetate	127-47-9	rs75201540	107697197	3	*CD47*	7.51E-09	0.008
Triamterene	396-01-0	rs61880875	7924360	11	*OR10A6*	7.54E-09	0.047
4-Chloro-3,5-dinitro-a,a,a-trifluorotoluene	393-75-9	rs146080764	49534816	11	*FOLH1*	7.58E-09	0.009
o-Nitrobenzyl chloride	612-23-7	rs113423597	53897175	4	*SCFD2*	7.59E-09	0.030
Vitamin D3	67-97-0	rs75048966	28220491	1	*C1orf38*	7.70E-09	0.004
1,1,1,2-Tetrabromoethane	630-16-0	rs190646915	1410097	X	*CSF2RA*	7.71E-09	0.004
Fumaronitrile	764-42-1	rs6575909	102802858	14	*ZNF839*	7.72E-09	0.022
t-Butyl formate	66-81-9	rs62389351	163302180	5	*MAT2B*	7.92E-09	0.003
Cycloheximide	762-75-4	rs116857952	69222338	6	*BAI3*	7.92E-09	0.098
Amiloride hydrochloride	2016-88-8	rs45477793	88470954	12	*CEP290*	7.98E-09	0.017
Dichloroacetonitrile	3018-12-0	rs114922911	186024997	4	*SLC25A4*	8.06E-09	0.004
p-Benzoquinone dioxime	105-11-3	rs116331855	182768941	2	*SSFA2*	8.08E-09	0.025
Vitamin D3	67-97-0	rs78073324	63616070	1	*FOXD3*	8.16E-09	0.004
1,8-Dihydroxy-4,5-dinitroanthraquinone	81-55-0	rs113434813	143835690	X	*SPANXN1*	8.24E-09	0.003
Nitazoxanide	55981-09-4	rs112997343	74356986	17	*SPHK1*	8.26E-09	0.002
Ergotamine tartrate	55981-09-4	rs4263901	50758525	X	*BMP15*	8.39E-09	0.002

[a]NCBI build 37. [b]Nearest gene. [c]Regression on inverse quantile normalized EC_{10} with covariates sex + 10 genotype principal components.

Table S7. SNP set pathway results.

Chemical set	Name	No. genes	Zscore[a]	FWER-controlled P^b
**1,1,1,2-Tetrabromoethane, CAS#630-16-0**				
KEGG	Allograft rejection	30	4.31	0.002
KEGG	Graft-versus-host disease	31	4.06	0.003
KEGG	Asthma	25	3.31	0.019
**2,2',4'-Trichloroacetophenone, CAS#4252-78-2**				
KEGG	Autoimmune thyroid disease	45	3.71	0.007
**HC blue, CAS# 233229-34-4**				
KEGG	Butirosin and neomycin biosynthesis	5	3.51	0.008
**13-cis-Retinal, CAS#472-86-6**				
KEGG	Butirosin and neomycin biosynthesis	5	3.67	0.010
**(1,3-Dimethylbutyl)-N'-phenyl-p-phenylenediamine, CAS#793-24-8**				
KEGG	Asthma	25	3.49	0.010
KEGG	Allograft rejection	30	3.47	0.011
**Azathioprine, CAS#446-86-6**				
GO.MF	Transforming growth factor beta receptor, pathway-specific cytoplasmic mediator activity	5	5.19	0.015
**2,3,4,5-Tetrachlorophenol, CAS#4901-51-3**				
KEGG	Natural killer cell mediated cytotoxicity	131	3.36	0.016
KEGG	Antigen processing and presentation	63	3.15	0.022
**Ziram, CAS#137-30-4**				
GO.BP	Regulation of chronic inflammatory response	7	5.26	0.017
**8-Hydroxyquinoline, CAS#148-24-3**				
KEGG	Mismatch repair	23	3.26	0.018
KEGG	Steroid hormone biosynthesis	52	3.24	0.020
KEGG	Porphyrin and chlorophyll metabolism	39	3.04	0.038
**Chlordane (technical grade), CAS#12789-03-6**				
GO.CC	Central element	5	4.34	0.034
**Permethrin, CAS#52645-53-1**				
GO.BP	Regulation of interleukin-2 production	34	5.08	0.037

[a]Z-score was computed by the gene_set_scan software. [b]Family-wise error controlled by resampling per chemical for each pathway type investigated.
